# Anomalous supply shortages from dynamic pricing in on-demand mobility

**DOI:** 10.1038/s41467-020-18370-3

**Published:** 2020-09-24

**Authors:** Malte Schröder, David-Maximilian Storch, Philip Marszal, Marc Timme

**Affiliations:** 1 Center for Advancing Electronics Dresden (cfaed), Technical University of Dresden, 01062 Dresden, Germany; 2 Institute for Theoretical Physics, Technical University of Dresden, 01062 Dresden, Germany; 3 Lakeside Labs, Lakeside B04b, Klagenfurt, 9020 Austria

**Keywords:** Nonlinear phenomena, Statistical physics, Economics

## Abstract

Dynamic pricing schemes are increasingly employed across industries to maintain a self-organized balance of demand and supply. However, throughout complex dynamical systems, unintended collective states exist that may compromise their function. Here we reveal how dynamic pricing may induce demand-supply imbalances instead of preventing them. Combining game theory and time series analysis of dynamic pricing data from on-demand ride-hailing services, we explain this apparent contradiction. We derive a phase diagram demonstrating how and under which conditions dynamic pricing incentivizes collective action of ride-hailing drivers to induce anomalous supply shortages. We identify characteristic patterns in the price dynamics reflecting these supply anomalies by disentangling different timescales in price time series of ride-hailing services at 137 locations across the globe. Our results provide systemic insights for the regulation of dynamic pricing, in particular in publicly accessible mobility systems, by unraveling under which conditions dynamic pricing schemes promote anomalous supply shortages.

## Abstract (web summary)

Dynamic pricing schemes are increasingly employed in on-demand mobility. Here the authors show that ride-hailing services across the globe exhibit anomalous price surges induced by collective action of drivers, uncovered from price time-series at 137 locations, and explain under which conditions they emerge.

## Introduction

**Fig. 1:**
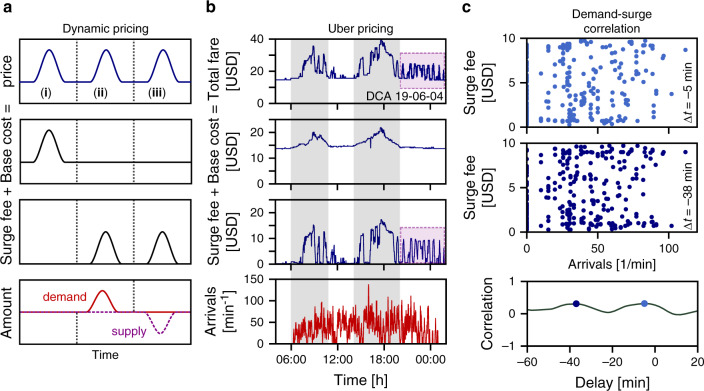
Dynamic pricing in on-demand mobility. **a** Schematic illustration of dynamic pricing. The total price separates into the base cost of the product or service and a supply and demand-dependent surge fee. Three fundamental mechanisms underlying price changes are (i) changes of the base cost, (ii) demand exceeding current supply levels, and (iii) supply shortage compared to current demand. Price adaptations (ii) and (iii) are intended to drive the system back to a supply–demand equilibrium. **b** The total fare for Uber ride-hailing services similarly decomposes into base cost and surge fee. Base cost depend on trip duration and reflect current traffic conditions while surge fees result from supply–demand imbalances. Both effects are illustrated here for trips from Reagan National Airport (DCA) to Washington Union Station in Washington, DC, USA. During commuting hours base cost increase because of longer expected trip duration during rush-hour (gray shading). The slower speed effectively reduces the supply of available drivers as they spend more time in traffic and naturally causes accompanying surge fees. During late evening and nighttime, the total fare exhibits repeated price surges triggered by supply–demand imbalances (dashed box) not reflected in the demand dynamics (passenger capacity of airplanes landing in DCA), consistent with recent reports on supply-driven price surges^17^. The identical magnitude of the price surges is the result of Uber limiting the maximum surge fee in response to these reports^29^. **c** Supporting the previous observation, no apparent correlation exists between the surge fee and the demand dynamics during the evening hours (20:00–02:00), even at 5 and 38 min delays, the two local maxima of the correlation function (see Supplementary Note 1 for a more detailed analysis).

Complex engineered systems are known to exhibit unintended states in their collective dynamics that often disrupt their function^1–5^. In complex mobility systems, examples include the emergence of congestion^6,7^, anomalous random walks in human travel patterns^8^, and cascading failures of mobility networks^9–11^. As urban mobility becomes more and more self-organized and digitized, mobility services increasingly employ dynamic pricing^12–16^, in general serving two main purposes (Fig. 1a). First, dynamic pricing adjusts the price of a product or service to compensate for changes in its intrinsic base cost. Second, it creates incentives for all market participants to equilibrate demand–supply imbalances by increasing the price if demand exceeds supply and vice versa. A higher price both imposes higher costs to customers incentivizing them to decrease their demand and, at the same time, offers higher profit for identical service to suppliers, in turn motivating them to increase their supply. However, recent reports on on-demand ride-hailing^17–19^ indicate that dynamic pricing may have the opposite effect and instead cause demand–supply imbalances.

Here, we quantitatively demonstrate the existence of these imbalances by comparing price time series and demand estimates for ride-hailing services. In a game theoretic analysis we reveal the incentive structure for drivers to induce anomalous supply shortages as a generic feature of dynamic pricing. This observation suggests that similar dynamics should emerge independent of the location or industry. Comparing price time series for 137 locations in 59 urban areas across six continents we find price dynamics reflecting anomalous supply shortages in several cities around the world.

## Results

### Dynamic pricing in on-demand mobility

Dynamic pricing schemes are commonly applied by on-demand mobility service providers, such as Lyft and Uber^15,16^. For Uber, the price *p* of the service (the total fare for a ride) decomposes into two parts^16^, base cost *p*_base_ and surge fee *p*_surge_,

1 $$p={p}_{{\rm{base}}}+{p}_{{\rm{surge}}}(D,S)\ ,$$

as illustrated in Fig. 1b for trips from Reagan National Airport (DCA) to Union Station in Washington, DC (see “Methods” section and Supplementary Table 1 for more details).

The first component (base cost) consists of regular fees for a ride

2 $${p}_{{\rm{base}}}={p}_{0}+{p}_{t}\ \Delta t+{p}_{l}\ \Delta l\ ,$$

including one-off fees *p*_0_ as well as trip fees *p*_*t*_ and *p*_*l*_ proportional to the duration Δ*t* and distance Δ*l* of the trip, similar to the fare for a typical taxi cab. These base cost increase, for example, during times of heavy traffic, such as morning and evening commuting hours (gray shading in Fig. 1b) when the trip duration Δ*t* increases due to congestion.

The second component (surge fee *p*_surge_) implements Uber’s surge pricing algorithm^16,20^ and reflects the time evolution of supply–demand imbalances. The surge fee increases due to persistent supply–demand imbalance during commuting hours. Longer trip duration means that drivers spend more time in traffic serving the same number of customers, which effectively reduces the supply of available drivers compared to the demand, and causes an increase of the surge fee. These price surges are meant to incentivize customers to delay their request, reducing the current demand, as well as to incentivize drivers to offer their service in areas or at times with high demand, increasing the supply.

As illustrated in Fig. 1b, during the evening the system settles to constant base cost, reflecting constant trip duration in uncongested traffic. Yet, even under these apparent equilibrium conditions, the time evolution of the surge fee exhibits a series of short, repeated price surges (dashed box in Fig. 1b) that are not reflected in the demand dynamics (Fig. 1c). In fact, recent reports about driver behavior at DCA^17–19^ indicate that drivers collaboratively stimulate price surges in the evening hours by temporarily switching off their app. Thereby, they cause artificial supply shortages, implying supply-side-induced out-of-equilibrium price dynamics at this airport consistent with our observations.

Using the observed price surges of confirmed anomalous supply shortages at DCA as a reference case, we address two key questions: First, what are the underlying incentives causing drivers to induce anomalous supply shortages and under which conditions do they emerge? Second, do these non-equilibrium dynamics emerge at other locations as well and how can we identify them without direct observation?

### Incentives promoting anomalous supply shortages

**Fig. 2:**
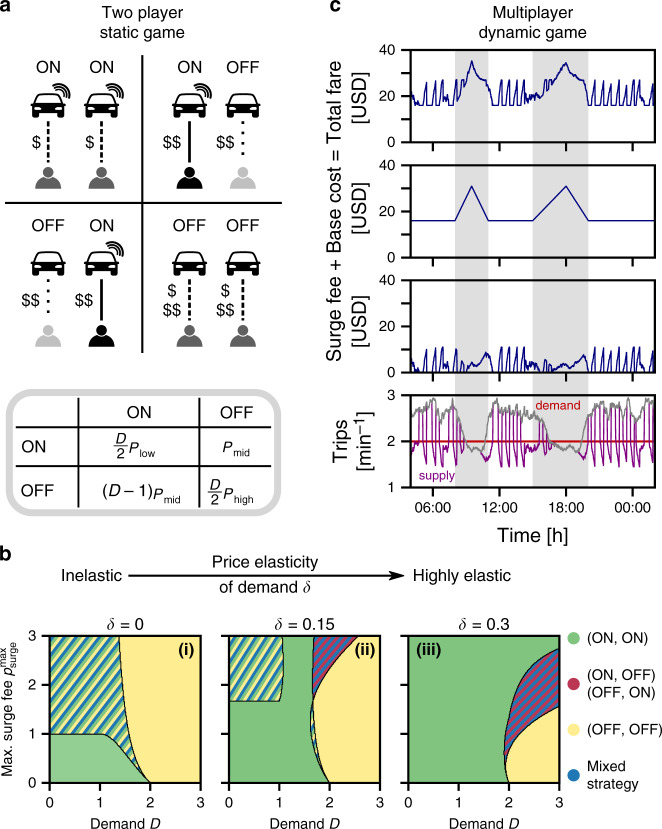
Incentive structure in dynamic pricing. **a** A two player game captures the fundamental incentives for drivers. Both drivers compete for a fixed average number of customers 1 ≤ *D* ≤ 2. The drivers may choose to temporarily switch off their apps to induce an artificial supply shortage and additional surge fees (see “Methods” section). If both drivers keep their apps on, both earn *p*_low_ ($) with probability *D*/2 (panel **a**, top left). If one driver switches their app off, the total fare increases to *p*_mid_ ($$). However, the other driver exploits their first-mover advantage to secure a customer, earning guaranteed *p*_mid_, while the offline driver only earns (*D*−1) *p*_mid_ from the remaining demand (panel **a**, top right and bottom left). If both drivers switch off their apps, they induce a larger supply shortage and thus a larger surge fee, resulting in the total fare *p*_high_ ($$$). Both drivers again share the demand equally when they go back online (panel **a**, bottom right). **b** Phase diagram of the resulting Nash equilibria. (i) If the demand is sufficiently large, the game is trivial and both drivers always go offline, triggering anomalous supply shortages (orange). At low demand the game becomes a prisoner’s dilemma^21^ and both drivers remain online (green) or stag hunt^22^ (hatched region with multiple coexisting Nash equilibria). (ii) and (iii) As the demand becomes more elastic and decreases as the price increases [Eq. (4)], drivers switching off their app risk missing out on a customer completely and the parameter range promoting artificial price surges becomes smaller (orange). Drivers are more likely to both remain online (green). At high demand and high surge fee, partial supply shortages emerge as equilibrium states where only one of the two drivers goes offline (red/blue hatched). These results are robust for nonlinear demand response (see Supplementary Figs. 18 and 19). **c** A dynamic game with multiple drivers (see “Methods” section and Supplementary Note 3) qualitatively reproduces the observed dynamics (compare DCA, Fig. 1b): Sustained non-zero surge fees occur during commuting hours with high base cost (gray). During non-commuting hours, drivers cooperate to induce artificial supply shortages to optimize their collective profit.

While the specific conditions promoting artificial price surges depend on local details and demand dynamics, a first principles game theoretic description captures fundamental incentives underlying the anomalous supply shortages: *S* = 2 drivers are competing for a fixed demand *D* aiming to maximize their expected profit (Fig. 2a). For illustration, we take a piecewise linear price function, representing the simplest possible demand-supply response, such that drivers earn the total fare

3 $${p}^{\prime}(S,D)=\left\{\begin{array}{ll}{p}_{{\rm{base}}}\hfill &{\rm{if}}\quad S\ge D\\ {p}_{{\rm{base}}}+{p}_{{\rm{surge}}}^{\max }\ \frac{D-S}{D}&{\rm{else}}\hfill\end{array}\right.$$

when they serve a customer, where *p*_base_ denotes the (constant) base cost and ${p}_{{\rm{surge}}}^{\max }$ denotes the maximum possible surge fee when *S* = 0 (see “Methods” section, Supplementary Note 3 and Supplementary Fig. 16 for details). Each driver has the option to temporarily not offer their service, contributing to an artificial supply shortage, *S* < 2. As drivers turn off their app, the fare increases from ${p}_{{\rm{low}}}={p}^{\prime}(2,D)$ with both drivers online over ${p}_{{\rm{mid}}}={p}^{\prime}(1,D)\ge {p}_{{\rm{low}}}$ as one driver goes offline to ${p}_{{\rm{high}}}={p}^{\prime}(0,D)\ge {p}_{{\rm{mid}}}$ when both drivers withhold their service. While drivers who do not offer their service would typically miss out on a customer, the use of online mobile applications in most ride-hailing services enables them to quickly change their decision. Turning their app back on, they can capitalize on the additional surge fee and earn the higher total fare by quickly accepting a customer before the dynamic pricing algorithm reacts (Fig. 2a, see “Methods” section for details).

Figure 2b illustrates the phase diagram of the resulting Nash equilibria. When the demand is inelastic and does not change as the price increases [Fig. 2b, panel (i)], at low demand and low surge fee the payoff structure of the game resembles a prisoner’s dilemma^21^, describing a conflict of interest between the drivers. While the socially optimal strategy for both drivers is to go offline, maximizing their total profit, each driver individually profits more from remaining online. Consequently, both drivers remain online due to the high risk of completely missing out on a customer if the other driver remains online (ON–ON equilibrium, green). The payoff structure changes to a stag hung^22^ with multiple Nash equilibria when the surge fee or the demand increases. If both drivers are online, neither profits individually from going offline, and vice-versa if both drivers are offline. Depending on the trust between the drivers, they settle into either an on–on (risk-averse) or an off–off (payoff-dominant and socially optimal) Nash equilibrium. In this regime, an additional mixed strategy Nash equilibrium also exists, where both drivers go offline with a certain probability. At high demand, the payoff structure becomes that of a trivial game without any conflict of interest between the drivers as both drivers always profit from inducing artificial supply shortages to earn the additional surge fee (OFF–OFF equilibrium, orange).

As the demand becomes elastic [Fig. 2b, panels (ii) and (iii)], i.e. the demand decreases in response to an increase of the total fare as

4 $$\begin{array}{r}{D}^{\prime}({p}^{\prime},D)=D\ (1-\delta \ ({p}^{\prime}-{p}_{{\rm{base}}}))\ \end{array}$$

governed by the price elasticity *δ*, the risk of missing out on a customer increases and profits due to surge fees are counteracted by the reduced demand. For a sufficiently strong demand response (high elasticity), the game setting effectively changes to low demand conditions when a single driver goes offline. The game becomes a prisoner’s dilemma or a trivial game where both drivers remain online (green). Consequently, the parameter region where drivers are incentivized to switch off their app (orange) shrinks. In particular, drivers are more strongly incentivized to create artificial price surges when the maximum surge fee is small. For intermediate conditions, a new state of partial supply shortages emerges, where only one of the two drivers goes offline (red-blue-hatched). This incentive structure is a generic property of the dynamic pricing, illustrated by its existence in this fundamental game-theoretic model and demonstrated for more than two players in Supplementary Fig. 20 and non-linear demand response in Supplementary Fig. 19.

Moreover, these incentives are sufficient to explain anomalous supply shortages in a time-continuous game under constant conditions (constant demand, a constant number of drivers and a constant price elasticity of demand) where the ON–OFF-decisions of the drivers, reacting to the current conditions, are the only remaining dynamics (Fig. 2c). Drivers contribute to an artificial supply shortage if sufficiently many other idle drivers are willing to also participate, following their mean-field optimal strategy. To avoid never making profit, however, individual drivers remain offline only for a short amount of time, explicitly limiting the timescale of potential artificial price surges (see “Methods” section and Supplementary Note 3 for details). The simulations shown in Fig. 2c reproduce qualitatively the same non-equilibrium price dynamics as observed in the recorded price data (compare Fig. 1b): Increases of the trip duration during commuting hours (gray shading in Fig. 2c) are accompanied by a sustained supply–demand imbalance and surge fees without drivers turning off their app. At other times, the drivers create short, artificial price surges to maximize their profit.

### Identifying characteristic price dynamics

The fact that these incentives are generic to dynamic pricing schemes suggests that artificial supply shortages and non-equilibrium surge dynamics emerge independent of the location. However, direct observation of the supply dynamics, e.g. of the number and location of online drivers, is typically impossible as this information is not publicly available. Even with the above results, a bottom-up prediction is practically infeasible since the exact conditions under which these dynamics are promoted depend on the specific details of the trip, the local dynamics of demand and drivers, publicly unavailable details on the surge pricing algorithm as well as additional external influences such as local legislation.

**Fig. 3:**
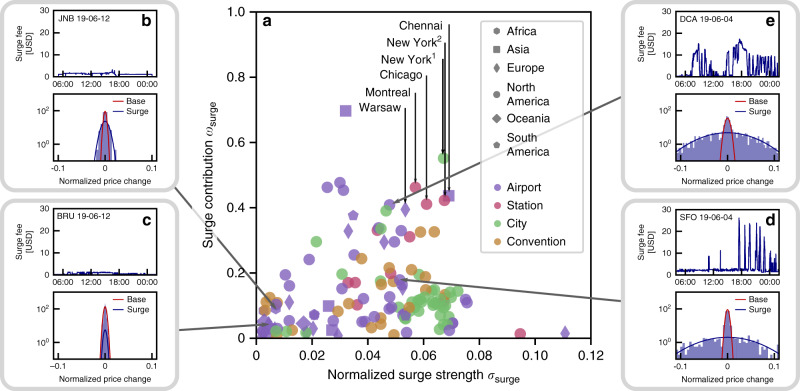
Characterizing non-equilibrium surge dynamics. The timescales of price changes characterize the surge dynamics at different locations. These price changes separate into small, slow changes *P*_base_ corresponding to varying base costs and fast, strong changes *P*_surge_ corresponding to the surge fee (red and blue line in the histograms in **b**–**e**, respectively). **a** Characterizing locations by the total weight *w*_surge_ of the surge component of the price change distribution and the magnitude *σ*_surge_ of the associated price changes reveals several locations [e.g., Warsaw, Montreal, Chicago, New York City [city(1) and station(2)] and Chennai] with similar surge characteristics to DCA (see Fig. 4 and “Methods” section and Supplementary Note 2 for more details and additional examples). **b** and **c** (left), Locations with low surge strength *σ*_surge_ exhibit no significant surge activity and no price changes on a fast time scale, shown here for Johannesburg (JNB, South Africa) and Brussels (BRU, Belgium). **d** (bottom right), Locations with high surge strength *σ*_surge_ and small surge contribution *w*_surge_ exhibit relatively few price surges (SFO, San Francisco, USA). **e** (top right), Locations with high surge strength *σ*_surge_ and high surge contribution *w*_surge_ exhibit a large number of fast price surges potentially driven by artificially induced supply shortages. Figure 4 confirms that the surge fee dynamics at these locations is indeed similar to the dynamics observed at DCA (Washington, DC, USA).

We overcome these obstacles by exploiting the characteristic temporal structure of the surge dynamics observed for confirmed anomalous supply shortages in DCA (compare Fig. 1b) to identify locations with similar dynamics. Based only on the price time series, we quantify the timescales of normalized price changes Δ*p* for 137 different routes in 59 urban areas across six continents (Fig. 3a, see “Methods” section for details). The distribution of price changes separates into a slow and fast timescale and a contribution where the price does not change

5 $$P\left(\Delta p\right)=	\, {w}_{{\rm{base}}}\ {P}_{{\rm{base}}}\left(\Delta p;{\sigma }_{{\rm{base}}}\right)\\ 	+{w}_{{\rm{surge}}}\ {P}_{{\rm{surge}}}(\Delta p;{\sigma }_{{\rm{surge}}})+{w}_{0}\ \delta (\Delta p)\ .$$

The slow price changes ${P}_{{\rm{base}}}\left(\Delta p;{\sigma }_{{\rm{base}}}\right)$ describe changes of the base cost varying as slowly as traffic conditions change during the day. The fast price changes ${P}_{{\rm{surge}}}(\Delta p;{\sigma }_{{\rm{surge}}})$ are associated with sudden changes of the surge fee. The last term *w*_0_ *δ*(Δ*p*) describes times when the price remains constant and contributes only at Δ*p* = 0, where *δ* represents the Dirac-Delta distribution and *w*_0_ the remaining weight *w*_0_ = 1−*w*_base_−*w*_surge_.

Characterizing the contribution *w*_surge_ of the surge fee and the magnitude *σ*_surge_ of the associated price changes with a maximum-likelihood Gaussian mixture model fit

6 $${P}_{x}(\Delta p;{\sigma }_{x})=\frac{1}{\sqrt{2\pi {\sigma }_{x}^{2}}}\ {\rm{{{e}}}}^{-\frac{\Delta {p}^{2}}{2{\sigma }_{x}^{2}}}$$

with $x\in \left\{{\rm{base}},{\rm{surge}}\right\}$ (see “Methods” section for details), we find locations without surge activity (Fig. 3b and c) as well as locations with strong but infrequent price surges (Fig. 3d). Importantly, we also identify several locations with price change characteristics similar to those observed at DCA, with a high magnitude and contribution of surge price changes, suggesting strong and frequent price surges potentially driven by anomalous supply dynamics (compare Fig. 3e).

**Fig. 4:**
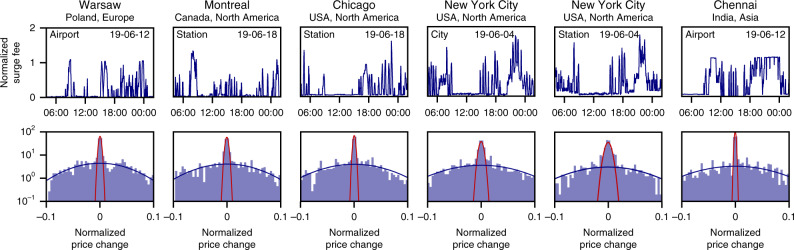
Identifying non-equilibrium surge dynamics and anomalous supply shortages. Repeated price surges similar to those observed at DCA (compare Fig. 3e) emerge in locations across the globe (America, Asia, and Europe) and independent of type of origin (airport, train station, and other prominent locations). The surge dynamics at the six locations identified in Fig. 3a is qualitatively and statistically similar to DCA. In particular, sustained periods with non-zero surge fee likely reflect a real supply–demand imbalance at that time while periods with repeated surge peaks are characteristic for price surges induced by artificial supply shortages (e.g. Warsaw evening, Montreal evening, Chicago evening, New York City afternoon and evening).

Indeed, all of the identified locations exhibit qualitatively similar non-equilibrium surge fee dynamics with a large number of repeated price surges, in particular during evening hours, demonstrating that the phenomenon is ubiquitous (Fig. 4, see Supplementary Figs. 14 and 15 for additional examples). While these results do not directly imply that all price surges at these locations are induced artificially, both the similarity of the timescale separation to confirmed artificial price surges and the universality of the incentives for drivers provide evidence supporting this conclusion.

## Discussion

In summary, we quantitatively demonstrated the emergence of non-equilibrium price dynamics in on-demand mobility systems at various locations across the globe and explained the fundamental incentive structure ultimately giving rise to such non-equilibrium price dynamics.

The exact conditions promoting anomalous supply shortages and artificial price surges depend on a multitude of factors at each location, such as users’ transportation preferences, working conditions for service providers, local legislation, and the availability of alternative transport options. Our methodology to classify the price dynamics based on the separation of timescales of price changes, without explicit knowledge about the time-resolved demand and supply evolution, enables a systematic search for supply anomalies based on price time series only. Although a direct observation of the supply dynamics may be required to confirm anomalous supply shortages, we identify a number of locations likely exhibiting anomalous supply shortages by combining confirmed reports and quantitative observations for reference cases, game-theoretically revealed generic incentive structures and large-scale time series analysis of recorded price estimates.

Our theoretical model demonstrates that the underlying incentives are a generic property of dynamic pricing and should even apply across industries where prices are adapted to supply and demand fluctuations on short timescales. This is particularly relevant for applications where prices are prescribed by an external algorithm instead of market clearing prices of buy and sell offers. One contemporary example may be recently discussed smart pricing schemes in power grids^14,23^, especially since large parts of the demand are inelastic due to fixed daily routines. Our results demonstrate that a carefully designed pricing scheme is essential to avoid unintended incentives that potentially reduce power grid stability instead of enhancing it.

For mobility systems in particular, characterizing the incentives and the conditions that promote artificially induced price surges suggests specific actions to suppress their emergence. This may include offering ride-sharing options^24–27^ (effectively lowering the demand, compare Fig. 2b) or providing more or alternative public transport options (effectively increasing the price elasticity of demand, compare Fig. 2b). The same incentives following a combination of few public transport options and a mismatch in driver availability and demand dynamics^28^ may also promote the emergence of supply anomalies particularly in the evening and at nighttime. Importantly, our results suggest that limiting the maximum surge fee, as done in response to the initial reports from DCA^29^ (see Fig. 1b) and frequently discussed as potential legislation^30,31^ (compare Chennai, Fig. 4), is not an effective response and may even result in the opposite effect if the demand is highly elastic.

In general, with the emergence of digital platforms, sharing economies and autonomous vehicle fleets, mobility services and other industries are becoming increasingly self-organized and complex such that new, potentially unintended collective dynamics can emerge^1,3–5,7,11,32^. Our results provide conceptual insights into these dynamics and may thereby support the creation and regulation of fair, efficient and transparent publicly available mobility services^24–27,33–35^.

## Methods

### Data sources and acquisition

In this work, we have recorded ~28 million ride-hailing price estimates for 137 routes of Uber rides in 59 urban areas across six continents between 31-05-2019 and 25-06-2019. We distinguish between four types of routes based on the origin location: 63 airport, 23 convention center, 12 train station, and 39 city trips (see Supplementary Note 4, Supplementary Fig. 1, and Supplementary Table 2 for detailed information, see Supplementary Data for precise GPS coordinates of the different routes).

For each route, we prompted total fare requests with a fixed interval via Uber’s price estimate API endpoint recording the price estimates for each route every 2–30 s. Per request, the API returned lower and upper total fare estimates for all Uber products operating in the local area, as well as estimated distance and duration of the trip which we equipped with the request timestamp. Using Uber’s products API endpoint, we complemented the price estimate data with information on local booking fee, price per minute, price per mile, distance unit, minimum fees, and the currency code parameter per product and location. We convert all price estimates to US Dollars based on currency exchange rates provided by the European Central Bank for the date of recording.

In all our analyses, we work with the lower estimate of the local economy product (UberX, UberGO in India).

### Data quality

We consider the data reliable because of the generally good availability of Uber’s service. Almost all data points are recorded with sub-minute intervals, most on an even finer scale. Individual locations have single gaps of up to 18 min between two recordings, though these are single occurrences out of a total 3–7 days of observations per location and thus do not affect the overall statistical analysis. While we chose origin and destination locations consistently by type for each location, rounding of the price estimates to integer values and granularity of reported trip duration may limit data quality for very short trips (see also Supplementary Table 2 for a detailed list of trips recorded). The resulting small fluctuations of the surge fee, however, are not captured by our timescale analysis as they are still small compared to the base cost and to actual price surges.

### Base cost

To determine the base cost (sum of pickup fee, trip fee, and surcharges) of a trip we first compute the trip fee based on the price per mile, price per minute, and the estimated trip length and duration. We add the pickup fee obtained from the Uber products API. Since data on the surcharges (e.g. airport fees or tolls) of individual trips is not available, we take surcharges to be constant for each trip. We subtract the pickup fee and trip fee from the price estimate, and take the minimum value of this remaining surge fee and surcharge cost as estimate of the surcharges, such that zero surge fee occurs at least once in the recorded price estimates.

### Surge fee

To estimate the surge fee time series, we subtract the base cost of the respective product from the total fare estimate. Since the available price estimates are rounded to integer values, the recorded price estimate may not reflect all changes of the trip fare especially for shorter trips with lower absolute total fare. This leads to small fluctuations in the extracted surge fee that do not correspond to actual surge activity.

### Airport arrival data

To estimate the demand for rides at airports, we record the number of arrivals at each of the 63 airports where we recorded price estimates. We collected aircraft landing times, call signs, and type of aircraft using flightradar24’s open API in the corresponding time frame, as well as information on the different aircraft’s current seat configuration obtained via flightera.net. We disregard entries without call signs or real landing times. In rare cases where no seat configuration was available, we estimate the number of seats as the average of all recorded flights with the same aircraft model (or the average over all aircraft models if no other similar model was recorded).

### Airport demand

We estimate the demand for ride-hailing services as proportional to the number of seats of all arriving airplanes (implying a constant fraction of potential Uber customers). To create a continuous time series from the discrete arrival events of individual airplanes we compute a 5 min moving average to create equidistant records every minute. This also slightly reduces the strong variations between minutes with and without arrivals.

Because we have much more frequent but not equally spaced data for the Uber price estimates, we use the same procedure and compute a 5 min moving average of the surge fee for every minute. This leaves us with the same granularity of the data as for the deplanements.

Using these data, we compute the cross-correlation between the Uber surge fee estimates and the deplanement data at the corresponding airport. In Fig. 1c we show the scatterplot at the timelag (deplanement earlier than surge) where this correlation is maximal for the illustrated window from 20:00 to 02:00 of the surge fee.

### Comparison of surge dynamics

To compare and characterize the surge dynamics for different trips we normalize the absolute surge fee time series by the base cost at that time, yielding an effective surge factor. For these normalized time series, we compute the per minute changes Δ*p* between consecutive time points (time *t* in minutes),

7 $$\Delta p(t)=\frac{{\text{total}}\; {\text{fare}}\,(t)}{\,{\text{base}}\; {\text{cost}}\,(t)}-\frac{{\text{total}}\; {\text{fare}}\,(t-1)}{{\text{base}}\; {\text{cost}}\,(t-1)}=\frac{{\text{surge}}\; {\text{fee}}\,(t)}{{\text{base}}\; {\text{cost}}\,(t)}-\frac{{\text{surge}}\; {\text{fee}}\,(t-1)}{{\text{base}}\; {\text{cost}}\,(t-1)}\ .$$

To quantify and compare the statistical properties of the surge factor time series we split the price changes into three contributions. We take any data point with Δ*p*^2^ < 10^−7^ to belong to a Dirac delta distribution at zero (not shown in the histograms) and fit a Gaussian mixture model with two Gaussian distributions to the remaining data. Taking both distributions to have a mean of zero (no price change on average) yields

$${\rm{Prob}}\left(\Delta p\right)= {w}_{0}\ \delta (\Delta p) +{w}_{{\rm{base}}}\ \frac{1}{\sqrt{2\pi {\sigma }_{{\rm{main}}}^{2}}}\ {\rm{{{e}}}}^{-\frac{\Delta {p}^{2}}{2{\sigma }_{{\rm{base}}}^{2}}} +{w}_{{\rm{surge}}}\ \frac{1}{\sqrt{2\pi {\sigma }_{{\rm{surge}}}^{2}}}\ {\rm{{{e}}}}^{-\frac{\Delta {p}^{2}}{2{\sigma }_{{\rm{surge}}}^{2}}}$$

where the weight *w*_surge_ defines the surge contribution and the standard deviation *σ*_surge_ is the normalized surge strength used to characterize the surge dynamics.

### Two player game—minimal theoretical model

The results presented in the manuscript (Fig. 2b) are obtained with normalized parameters *p*_base_ = 1 and $\delta \in \left\{0,0.15,0.30\right\}$, allowing up to ${p}_{{\rm{surge}}}^{\max }=1/0.30\approx 3.33$ before no customer orders a ride at the maximum surge fee. See Supplementary Note 3 for a detailed description.

### Dynamic multiplayer game

For the dynamic multiplayer game, we consider a single origin location with *N* = 160 drivers. Upon completing a trip, drivers return to the origin location after a total round-trip time *t*_s_ uniformly distributed in $\left[\left\langle {t}_{{\rm{{s}}}}\right\rangle -5,\left\langle {t}_{{\rm{{s}}}}\right\rangle +5\right]$ minutes. We increase the round-trip time from the base value $\left\langle {t}_{{\rm{{s}}}}\right\rangle =30$ min to $\left\langle {t}_{\rm{{{s}}}}\right\rangle =60$ min in the morning and afternoon (starting at 08:00 and increasing linearly up to the maximum at 9:30 and back to the base value until 11:00. Similarly in the afternoon from 15:00 to the maximum at 18:00 and back until 20:00).

The base cost *p*_base_ depend linearly on the round-trip time as ${p}_{{\rm{base}}}=1+\left\langle {t}_{{\rm{{s}}}}\right\rangle /2\in \left[16,31\right]$ USD as the round-trip time changes during the day. Similar to the two-player game, we take a linear price dependence for the surge pricing as

8 $${p}^{\prime}(t)=\left\{\begin{array}{ll}{p}_{{\rm{base}}}\hfill &{\rm{if}}\quad {N}_{{\rm{idle}}}(t)\ge {N}_{{\rm{thresh}}}\\ {p}_{{\rm{base}}}+{p}_{{\rm{surge}}}^{\max }\left(1-\frac{{N}_{{\rm{idle}}}(t)}{{N}_{{\rm{thres}}}}\right)&{\rm{else}}\hfill\end{array}\right.$$

based on the number *N*_idle_ of online drivers at the trip origin and the number of drivers *N*_thresh_ before the surge fee becomes non-zero. We take ${N}_{{\rm{thresh}}}=\lambda \ <{t}_{{\rm{{s}}}}>$, where *λ* = 2 requests per minute describes the demand modeled as a Poisson process in time. We model responses of the price to the current system state (number of available drivers and round-trip time) as instantaneous.

The behavior of customers and drivers is as follows: Each customer *i* is assigned a uniformly random maximum price ${p}_{\max ,{\rm{i}}}\in [{p}_{{\rm{base}}},{p}_{\max }]$ they are willing to pay, where we take ${p}_{\max }=54$ USD. When the customer makes a request, they check the current total fare. If the current total fare is smaller than ${p}_{\max ,{\rm{i}}}$, the customer orders the ride. If the total fare is higher or no drivers are online and idle, the customer waits and checks again every 2 min. After 10 min without ordering a ride, the customer leaves the system.

At every point in time the drivers decide whether to switch their app off or on. They make this decision based on the (mean field) optimal strategy to optimize their collective payoff. A driver switches off their app only if two conditions are fulfilled: first, if there are sufficiently many drivers available and willing to be offline to induce a non-zero surge fee. Second, if the price is less than the (mean field) optimal value for the drivers given the current system state. Each driver remains offline for at most 20 min. After this time, the driver only considers going offline again after serving a customer (drivers try to obtain similar individual profits whereas their optimal strategy based on maximizing their collective profit would be for some drivers to be always offline).

### Reporting summary

Further information on research design is available in the Nature Research Reporting Summary linked to this article.

## Supplementary information

Supplementary Information

Supplementary Data 1

Reporting Summary

## Data Availability

A list of aggregate statistics for all recorded trips containing the exact origin and destination coordinates, as well as measurement parameters and numerical analysis results (presented in Figs. 3 and 4) is available as Supplementary Data. The full original data is available on reasonable request to the authors.

## Code availability

Full details on the data analysis and game-theoretic modeling are provided in the Supplementary Information. The code is available on request to the authors.
