# Supplementary material for: Anomalous supply shortages from dynamic pricing in on-demand mobility
Source: Nat Commun. 2020 Sep 24;11:4831. doi: 10.1038/s41467-020-18370-3 (PMC7515901; doi:10.1038/s41467-020-18370-3)
Supplement: Supplementary file 1 — Supplementary Information [file 41467_2020_18370_MOESM1_ESM.pdf]

Supplementary Information

accompanying the manuscript

Anomalous supply shortages from dynamic pricing in on-demand mobility

Malte Schröder*,[1,2] David-Maximilian Storch*,[2] Philip Marszal*,[1,2] and Marc Timme[1,2,3]

[1] *Chair for Network Dynamics, Center for Advancing Electronics Dresden (cfaed), Technical University of Dresden, 01062 Dresden, Germany*

[2] *Institute for Theoretical Physics, Technical University of Dresden, 01062 Dresden, Germany*

[3] *Lakeside Labs, Lakeside B04b, 9020 Klagenfurt, Austria*

(Dated: August 10, 2020, *these authors contributed equally to the manuscript)

In the main manuscript we illustrate how inherent incentives of dynamic pricing induce anomalous supply dynamics and artificial price surges in ride-hailing services. We show that the incentives driving these dynamics are generic to dynamic pricing schemes and analyze the specific conditions that promote them. Based on confirmed reports of these non-equilibrium dynamics at Reagan National Airport (DCA) in Washington, D.C. [1], we identify locations with similar surge fee dynamics from Uber price estimates for trips originating from 63 airports around the globe as well as 23 convention centers, 12 train stations and 39 city trips in North America (largely US).

In this Supplementary Information we provide additional details on all analyses and results presented in the main manuscript. This document is structured as follows:

Supplementary Note 1. Dynamic pricing in ride-hailing

We explain Uber's dynamic pricing model and isolate the surge fee component influenced by supply and demand imbalances. We give a detailed comparison of the surge activity to the estimated demand at DCA and other airports.

Supplementary Note 2. Statistical properties of surge pricing

We compare the surge fee dynamics of all recorded price estimates to identify locations with similar surge activity to DCA that may be driven by anomalous supply dynamics.

Supplementary Note 3. Incentive structure for drivers under dynamic pricing

We explain the incentives that determine the action of drivers under dynamic pricing in a simplified game-theoretic setting.

Supplementary Note 4. Data, methodology and details

We provide a detailed explanation of data collection and summarize our data processing approach.

Supplementary Note 1. Dynamic pricing in ride-haling

Dynamic pricing is a general mechanism to adjust prices to time-varying conditions. Application of dynamic pricing schemes are widespread in online retail and used, for example, by Amazon [2], and is of increasing importance in transportation contexts, too. In particular for ride-hailing services, where service conditions may vary strongly over the day due to weather, special events or simply rush hour and congestion, many service providers apply dynamic pricing [3, 4].

Uber Technologies Inc. is a major ride-hailing platform operator that matches drivers with customers requesting transportation in 795 metropolitan areas worldwide (as of July 2019 [5], Supplementary Fig. 1). The company operates a digital marketplace for transportation services where riders voice their demand for a specific trip and drivers offer to deliver the service. The dynamic pricing scheme employed by Uber includes both an adaptive trip fee, based on local traffic conditions and similar parameters, as well as an additional component to balance the spatio-temporal distribution of demand and supply in the operating area, commonly denoted as surge pricing [3, 6].

In this section, we detail the different components of the pricing mechanism and isolate how they impact the evolution of the recorded price estimate time series.

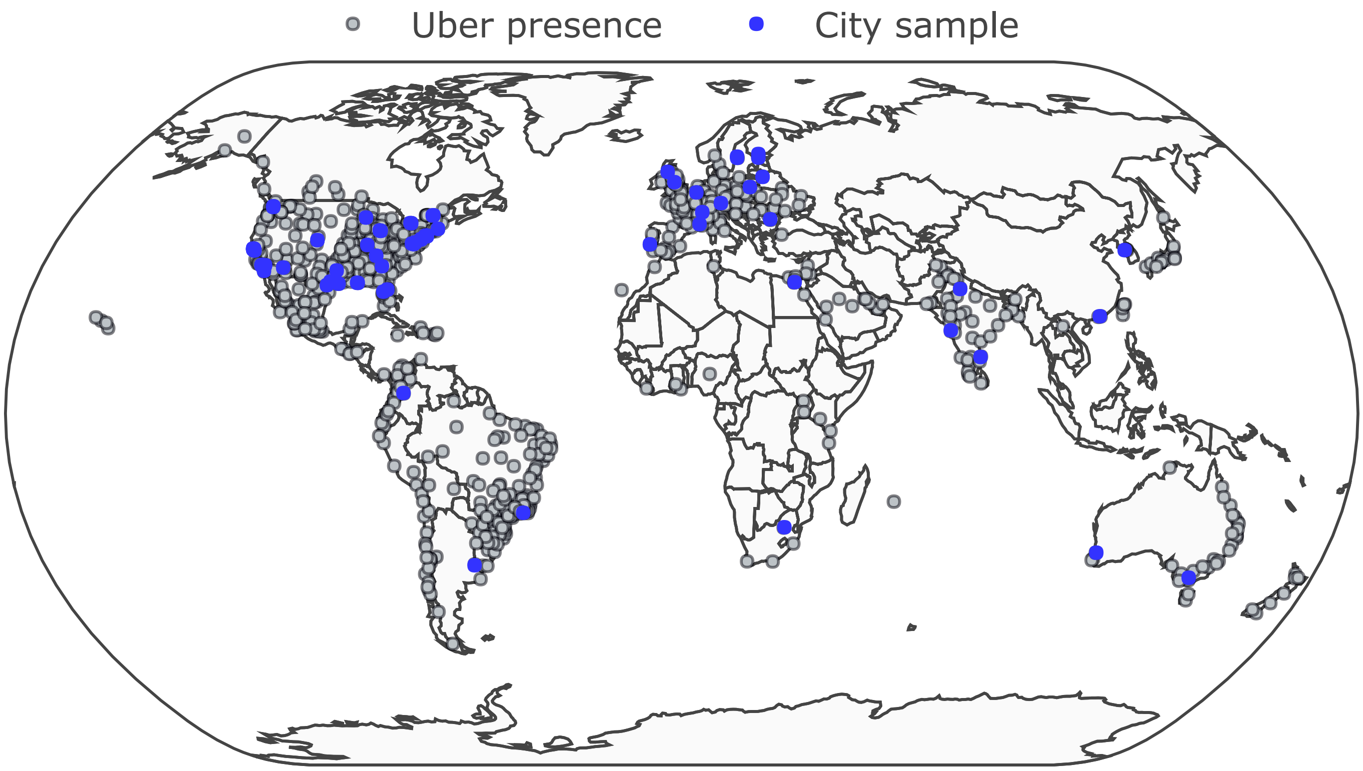

Supplementary Figure 1. **Uber ride-hailing services around the world.** Blue markers indicate all cities where price estimate time series have been recorded out of all locations where Uber service is available (grey). In some locations, especially in the US, we recorded price estimates for multiple different trips in the same location (see Supplementary Tab. II in Supplementary Note 4 for details).

Uber's price model

Uber's dynamic pricing model consists of four different fee categories[1]:

- **Pickup fee**: cost for customer pick-up from the requested origin point of the trip.

  Pickup fees contain a base fare (flat fee per pick-up) and may be subject to additional long-pickup fees if drivers and customers are far away from one another, e.g. when requesting a ride from a remote location. Long-pickup fees apply if the pickup duration exceeds a pre-defined threshold value (e.g. 10 minutes in many US cities), and is charged for certain Uber products in selected operating areas only.

  Long-pickup fees are calculated based on the distance and time the driver has to invest to pick up the customer, and is bound from above by a maximum pickup fee. Price per minute and price per mile parameters determine the absolute amount of the fee.

  $$\text{Pickup fee} = \text{base fare} + \text{long-pickup fees (optional)}$$

  Input parameters: base fare, price per minute (optional), pickup duration (optional), price per mile (optional), pickup distance (optional)

- **Trip fees**: cost for the passenger transportation component of the ride.

  Trip fees consist of a fixed booking fee as well as variable per minute and per mile prices. The trip fee is lower bounded by the minimum fare parameter.

  $$\text{Trip fee} = \text{booking fee} + \text{distance fee} + \text{time fee}$$

  Input parameters: booking fare, price per minute, trip duration, price per mile, trip distance, minimum fare

- **Surcharges**: optional add-on fees for the specific type of ride.

  Surcharges may reflect additional fees such as tolls, airport pickup fees, tip, ..., and thus depend on the trip specifics.

  $$\text{Surcharges} = \text{tolls} + \text{airport pickup fees} + \ldots$$

  Input parameters: tolls (optional), airport pickup fees (optional), tip (optional), ...

- **Surge fee**: supply- and demand-based cost increment.

  Surge fees consider the spatio-temporal distribution of ride requests and available drivers. They reflect a price increment on trip and base fares, either in terms of a surge multiplier or as an additive surge component that is intended to rebalance local demand and supply [6, 8].

  Input parameters: Unknown. Uber's surge pricing algorithm is not public.

The total fare for the ride is the sum of these four components

$$\begin{aligned}\text{total fare} &= \underbrace{\text{pickup fee} + \text{trip fee} + \text{surcharges}} + \text{surge fee} \\ &= \qquad\qquad \text{base cost} \qquad\qquad + \text{surge fee}\,, \end{aligned} \tag{S1}$$

which we denote as trip dependent base cost and supply-demand dependent surge fee.

[1] This information on Uber's price model is based on Ref. [7], accessed on 19-06-24, and may be subject to change.

Depending on the city, the values of the input parameters for the different price components may vary. Moreover, Uber offers different products that differ by their level of service, and have different input parameters to the pricing model. In our analysis, we focus on the standard service and, for comparison, a corresponding premium service available in the region:

- **Standard service**: UberX, UberGO (India)
- **Premium service**: Black, Berline (France), Exec (Great Britain), Lux, Premier (India) or Select (Egypt, Argentina)

More details can be found in the data collection section at the end of this Supplementary Information. For all data presented in this section UberX and Black are available and results are based on price estimates for these services. Supplementary Table I gives an exemplary overview of several parameter values serving as inputs to the Uber price mechanism for four US cities.

		**Washington D.C.**		**Los Angeles**		**San Francisco**		**Houston**	
		Black	UberX	Black	UberX	Black	UberX	Black	UberX
**Pickup fees**	Base fare [USD]	7.75	1.21	8.75	0.00	8.75	2.20	7.75	1.00
	Long-pickup fee [USD]	-	Variable	-	Variable	-	Variable	-	Variable
	- Price per minute [USD]	-	0.30	-	0.28	-	0.39	-	0.17
	- Price per mile [USD]	-	0.80	-	0.80	-	0.91	-	0.80
**Trip fees**	Booking fee [USD]	0.00	2.00	0.00	2.30	0.00	2.20	0.00	2.90
	Minimum fare [USD]	15.75	7.00	15.75	5.80	15.75	7.20	15.75	5.95
	Price per minute [USD]	0.82	0.30	0.71	0.28	1.08	0.39	1.03	0.17
	Price per mile [USD]	2.20	0.80	2.92	0.80	2.73	0.91	2.20	0.80
**Surcharges**	Airport pickup fee	4.00	4.00	5.00	4.00	N/A	N/A	0.00	2.75
**Surge fee**	Supply/demand balance	N/A	N/A	N/A	N/A	N/A	N/A	N/A	N/A

Supplementary Table I. **Input parameter values determining Uber prices differ per product and locality.** Price parameters for Uber's standard (UberX) and premium (Black) ride-hailing services in four exemplary US cities [7].

Time series of Uber price estimates

Supplementary Figure 2a shows price estimate time series for four exemplary trips originating from airports in Washington, D.C. (DCA), San Francisco (SFO), Los Angeles (LAX) and Houston (IAH) for Uber Black and UberX over the time span of 24 hours. Trip fares change dynamically in all of the four cities and exhibit a slow and fast timescale of price volatility. While the slow dynamics modulates the price in timescales of several hours, the fast timescale adds price spikes in the order of ten minutes to half an hour. Here, we isolate the different contributions from pickup fees, trip fees, surge fee, and surcharges to the price evolution.

The price dynamics shown in Supplementary Fig. 2a is driven by trip fees (see Supplementary Fig. 2b) and surge fees (see Supplementary Fig. 2c) which vary over time. We assume that pickup fees and surcharges are constant for all time series analyzed. Possible exceptions include tolls which apply during the day but not at night or are applicable only on alternative routes (e.g. for IAH). However, these surcharges are typically small and change over very long timescales (~ 12 hour) and do not significantly alter the observed surge dynamics.

Trip fees vary as a function of intra-day variation in local traffic conditions in each of the four cities. Two effects superimpose: On the one hand, trip duration estimates change as the streets from the different airports to the respective inner-city destinations become congested during commuting and business hours (see Supplementary Fig. 3 for trip duration estimates). Hence, the time-dependent trip fee increases and decreases over the course of the day proportional to the street flow traffic conditions. On the other hand, route choice recommendations change as a function of the current traffic conditions. As traffic intensifies, alternative routes may become faster and thus more attractive to complete the trip. However, those trip duration-preferable routes might be longer compared to the shortest-distance path, implying higher distance-dependent trip fees (see Supplementary Fig. 3, note in particular DCA, where the trip distance increases only when the trip duration is large.). Together, both contributions define the time-dependent trip fee component of the total fare estimate. Trip duration and distance estimates are identical for both Uber Black and UberX. Hence, their trip fees evolve synchronously, though with different per-minute and per-mile charges (see Supplementary Fig. 2b and Supplementary Tab. I).

In Supplementary Fig. 2 (right column) we subtract pickup fees, trip fees and (estimated constant) surcharges from the price estimate time series to isolate the time evolution of the surge fee component (see Supplementary Note 4 for more details). For Uber Black, there is almost no surge activity at DCA, LAX or IAH over the illustrated time span of 24 hours. Similarly, SFO does not exhibit Uber Black price surges for most of the day, but only a single distinctive surge at 00:30. Hence, we assume that the marketplace for Uber Black is in equilibrium at all airports, and there are no stark spatio-temporal supply and demand imbalances.

For UberX, the surge fee dynamics differs substantially. At DCA, we observe substantial surge activity for most of the day. Only for four hours at night time the marketplace does not give rise to surge fees (consistent with the typically low demand for transportation during this time window. Longer price surges exist for approximately 1.5 hours during morning (07:30 to 09:00) and 3.5 hours during evening commuting hours (16:00 to 19:30). It is plausible that these price surges supplement the typical high commuting demand during rush hour. Additionally, the dynamic pricing mechanism induces a series of short, characteristic, almost periodic surges with approximately universal peak value of ten USD and duration of 20 minutes that appear between 19:00 and 03:00. Similar surge dynamics with short, repeated surges are clearly visible at SFO between 18:00 and 23:00, possibly between 23:30 and 01:30 at LAX, but not at IAH.

Given the long-term presence of Uber in any of these cities (launch dates: San Francisco in May 2010, Los Angeles in March 2010, Washington, D.C. in December 2011 and Houston in February 2014 [9]) and the difficulty for the drivers to operate economically sustainable in a market with too many competitors for given demand, it is reasonable to assume that the different marketplaces are equilibrated with respect to long term fluctuations. In fact, the Uber Black surge dynamics supports this hypothesis and even suggest equilibration down to small, intra-day timescales, with drivers having adjusted to when and where to work efficiently. In contrast, the repeated, sudden surges of UberX indicate out-of-equilibrium dynamics. Counter-intuitively, the dynamic pricing mechanism seems to prevent the system from settling into an equilibrium.

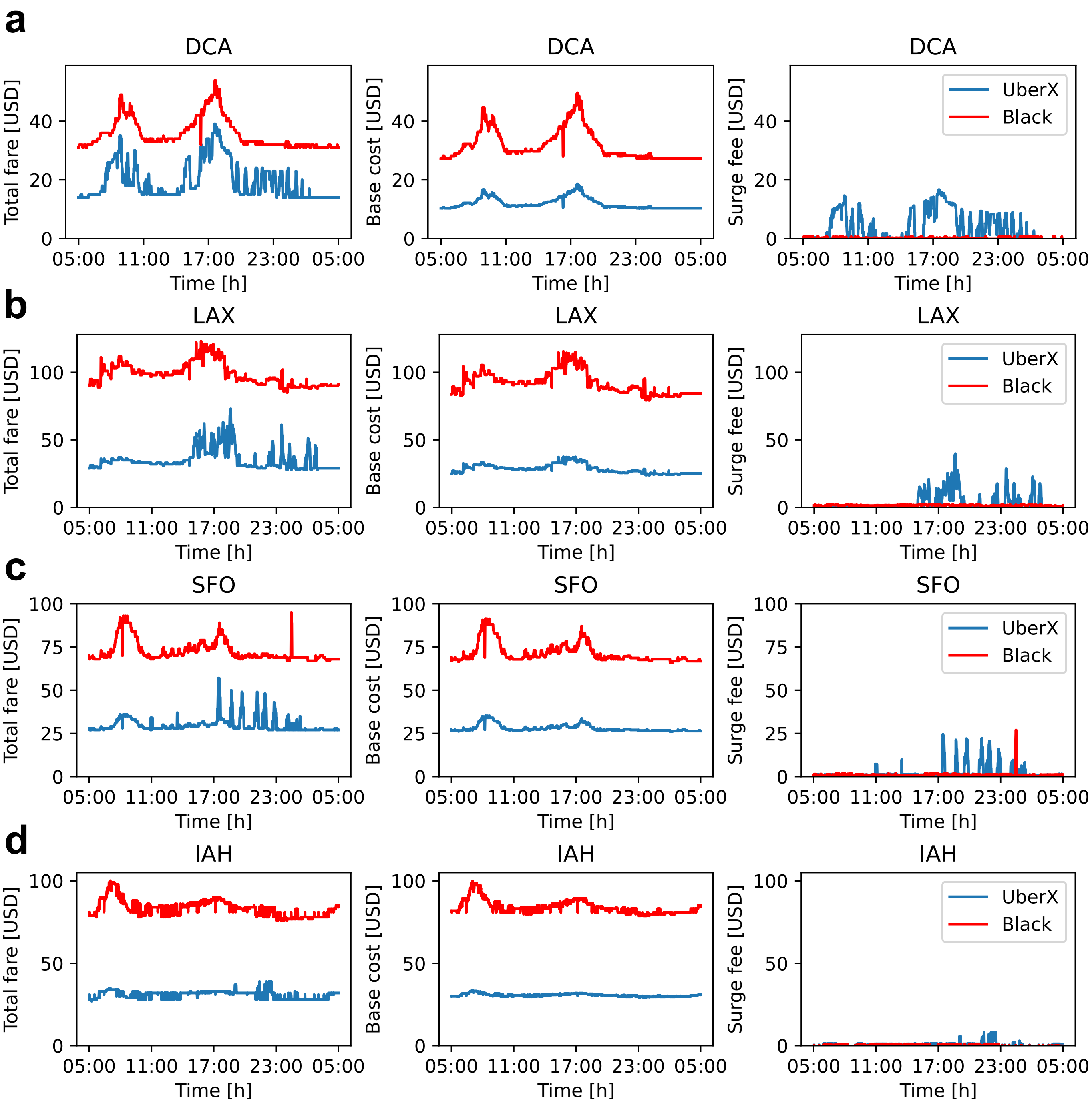

Supplementary Figure 2. **Uber Black and UberX trip fees evolve synchronously while the respective surge fees do not**. **a-d** Fare estimates for UberX (blue) and Uber Black (red) on 19-06-04 for trips originating from Reagan National Airport, Washington, D.C. (DCA), Los Angeles International Airport (LAX), San Francisco International Airport (SFO) and George Bush Intercontinental Airport, Houston (IAH). Columns show total fare, base cost and surge fee, respectively (from left to right).

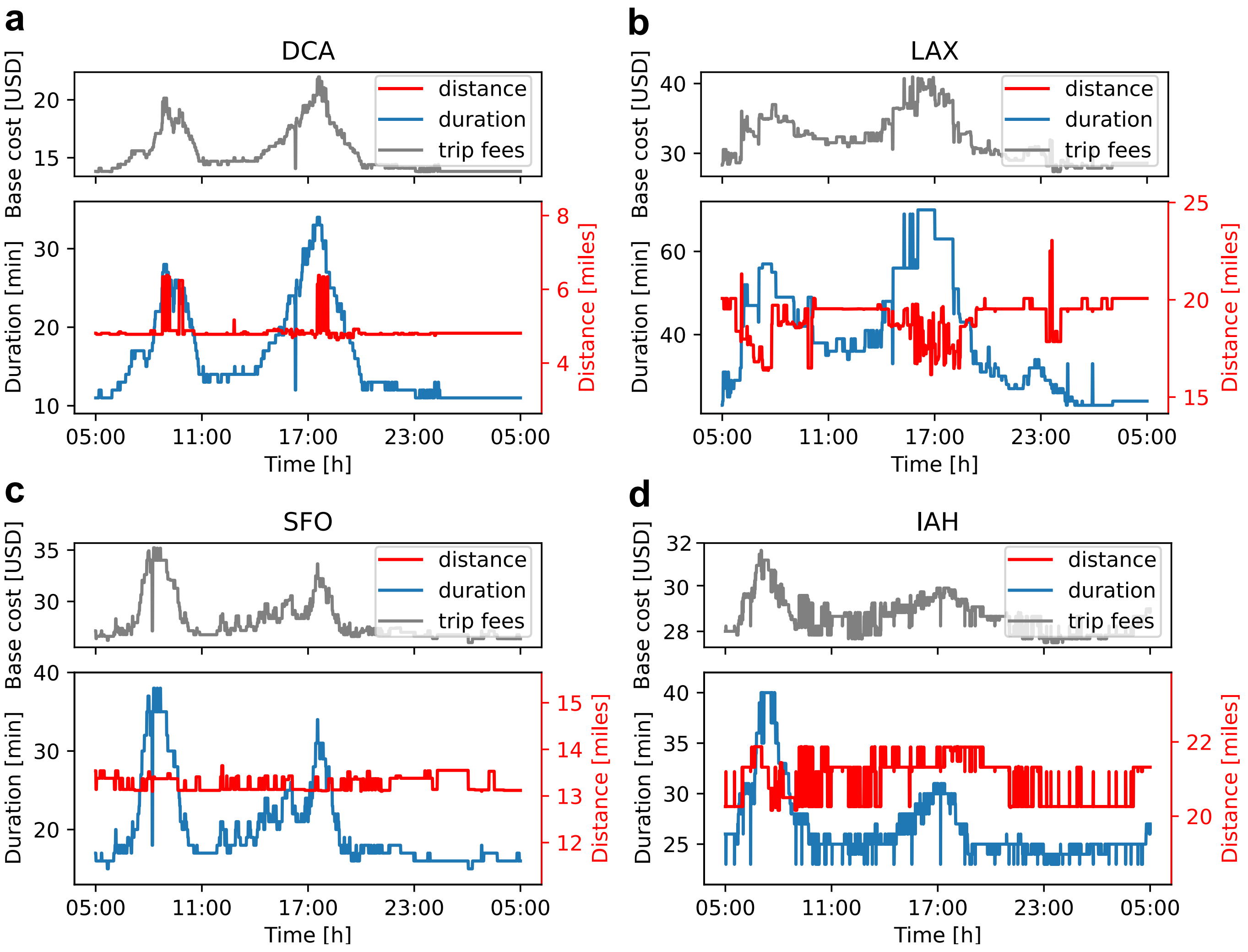

Supplementary Figure 3. **Uber trip fees change proportional to city traffic flow conditions**. UberX base cost change intra-day at **a** DCA, **b** LAX, **c** SFO and **d** IAH (data from 19-06-04, compare Supplementary Fig. 2) as trip fees adapt to latest estimates for trip duration (blue) and distance (red). Both estimates respond to changing traffic conditions, congestion and dynamic route choice. During commuting hours rising trip duration estimates reflect congested streets, while changes in trip distance estimates correspond to alternative route choice advise as the fastest route option changes for given traffic conditions. Uber Black trip fees behave qualitatively identically (compare Supplementary Fig. 2) since they are based on the same duration and distance estimates.

Demand model

Possible origins for the surge dynamics are either local fluctuations in the demand (i.e. many customers requesting a ride at the same time) or changes in the supply (i.e. few available drivers). While driver induced price surges are confirmed to occur at DCA [1], we do not know which, if any, of the observed peaks correspond to these artificial prices surges. To identify parts of the surge fee time series that are likely caused by supply-side action, we develop a demand model for Reagan National Airport (DCA) and assess to what extent it explains the price dynamics observed in Supplementary Fig. 2 (top right). In particular, we estimate the demand based on historic airport taxi departures and recorded aircraft deplanements. We find that a large part of the surge dynamics cannot be satisfactorily explained through the demand model, suggesting that in particular the short, repeated surges in the evening are primarily induced by changes of the supply.

Historic taxi trip records

Uber's ride-hailing service operates in the same niche as traditional taxi services. According to the Department For-Hire Vehicles, four of Washington D.C.'s large ride-hailing companies (HopSkipDrive, Lyft, Uber and Via) generated 45% of the combined taxicab and ride-hailing tax revenues in 2016, 59% in 2017 and 67% in 2018 [10]. As the absolute amount of those tax revenues remained approximately constant over the three years (2016: 6.75 million USD, 2017: 7.65 million USD, 2018: 7.19 million USD), ride-hailing companies kept on gaining market share from traditional taxi providers, underpinning that digital ride-hailing and traditional taxi services are substitutes. This suggests that taxis and ride-hailing services serve a similar demand and recorded taxi trips departing from DCA likely reflect the typical intra-day demand also for ride-hailing services.

Supplementary Figure 4 shows the average intra-day taxi demand evolution for trips originating from DCA airport, calculated for trips from January to August in 2017. and, interestingly, do not reflect the rush hour traffic observed in Supplementary Fig. 3. In particular on Monday, Tuesday and Wednesday the average demand is approximately constant over the whole day with 1.85 rides per minute (standard deviation 0.37 rides per minute). Higher demand in the evening at other days may strengthen price surges during that time but no specific correspondence between taxi demand and the dynamics of the surge fee is visible.

Overall, there seems to be no direct influence of the general demand evolution on the surge fees, consistent with our assumption of an equilibrated market on these timescales. However, the information about events of a specific day is lost by considering average historic demand data. Events of an individual day may reveal more direct correlations between the demand and the surge fee. In the following, we therefore attempt to match the surge fee with the demand (aircraft arrivals) of the respective day.

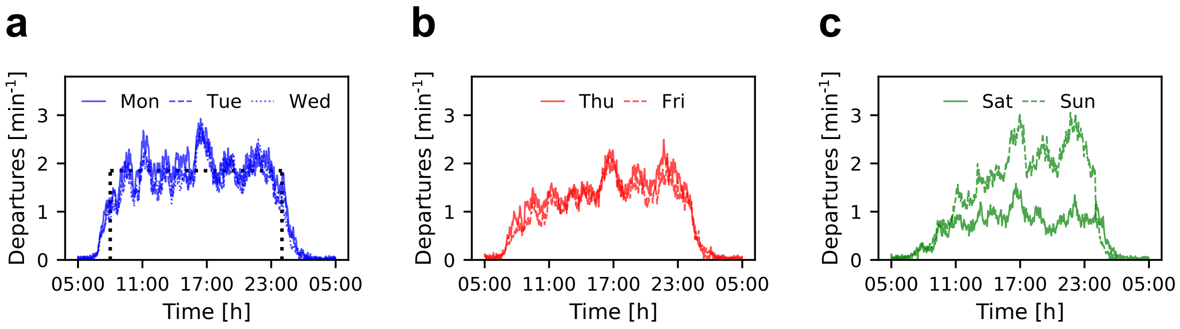

Supplementary Figure 4. **Average demand for taxi trips from DCA is approximately constant during the day.** Average number of taxi departures per minute from DCA recorded between 17-01-02-17-08-27. **a** From Monday to Wednesday, the average demand during business hours is approximately constant at 1.85 taxi rides per minute (dashed black line). **b,c** Thursday through Sunday show a slightly higher demand in the evening. In general, larger fluctuations typically occur over one to two hours. Details on data processing can be found in Supplementary Note 4.

Aircraft passenger arrivals

The taxi trips analyzed above only provide the average demand across many days. However, the surge dynamics of a specific day depend on the demand due to the specific arrival pattern of airplanes on that day.

Passengers arrive in DCA with each aircraft deplanement. Some of these passenger may continue on a connecting flight while the remainder likely travels in the direction of Washington, D.C., using for example ride-hailing services like Uber or taxi cabs. Taking a homogeneous load factor and a constant share of Uber customers across all arriving planes, we expect the demand for ride-hailing services to be proportional to the seat capacity of the arriving aircraft. Supplementary Figure 5a shows the time series of the UberX surge fee together with the corresponding capacity of arriving aircraft (compare DCA, Supplementary Fig. 2).

The capacity of arriving aircraft follows a similar pattern to the average taxi demand above. At nighttime almost no deplanements are observed, in agreement with the DCA Nighttime Noise Rule. Over the course of the day, DCA handles a near constant stream of aircraft landings, in line with the approximately constant demand for taxi service. Deplanements occur with a median interval of 2 minutes with aircraft equipped with with 50 to 213 seats. On average, 32 passengers arrive in DCA per minute, a fraction of which will use ride-hailing serivces.

To estimate the influence of the arrivals on the surge dynamics, we compute the normalized cross-correlation function

$$\rho(\Delta t) = \frac{E\left[\left(S(t) - \langle S(t)\rangle\right)\left(A(t+\Delta t) - \langle A(t+\Delta t)\rangle\right]}{\sigma_S(t)\sigma_A(t+\Delta t)} \tag{S2}$$

between fixed windows of the UberX surge fee $S(t)$ and the capacity of arriving airplanes $A(t)$ with time lag Δt. $E[\cdot]$ denotes the expectation value of the argument and σ_S and σ_A denote the standard deviation of the respective time series in the corresponding time window. For more details on the data preparation and processing see Supplementary Note 4.

To avoid high correlations simply due to the significant night-day differences in both airplane arrivals and ride-hailing demand, we calculate the cross-correlation $\rho(\Delta t)$ only for time windows between 08:00 and 02:00 on the next morning

- 6-hour windows: We analyze morning time windows between 08:00 to 14:00 (Supplementary Fig. 5b, yellow lines), afternoon time windows from 14:00 to 20:00 (green lines), and evening time windows from 20:00 to 02:00 (blue lines).
- 12-hour windows: We contrast the 6-hour time widows with half-day daytime windows between 08:00 to 20:00 (Supplementary Fig. 5b dotted gray lines) and late-afternoon to nighttime windows between 14:00 to 02:00 (dotted-dashed gray lines)
- 18-hour windows: Furthermore, we define an 18-hour time window between 08:00 to 02:00 extending over the full time span of surge activity (Supplementary Fig. 5b solid gray lines).

In particular, we focus on the correlation of the 6-hour evening time window from 20:00 to 02:00 (see Supplementary Fig. 5, shaded in panel a, dark blue line in panel b), where the trip fee is constant and we expect no influence of traffic conditions on the surge fee. In this time window, we observe repeated peaks of the surge fee and supply-induced price surges are known to occur [1].

The first maximum of the cross-correlation function is obtained for a time lag of $\Delta t^* = -5$ minutes at a value of $\rho(\Delta t^*) = 0.31$, indicating only a weak direct influence of aircraft arrivals on surge dynamics. Several similar maxima of the correlation correspond to the 30 to 40 minute periodicity of the surges in this time window. In contrast, this periodicity is not reflected in the aircraft arrivals. On other days, the correlation reaches up to $\rho(\Delta t^*) \approx 0.39$ in the same time window. The correlation at $\Delta t^* = -5$ minutes may be explained by passengers hailing a ride or checking prices very quickly after landing, possibly while taxiing to the gate or immediately after exiting the plane.[2] However, a scatter plot at the time lag of the maximum correlation reveals no clear relationship between the surge fee and aircraft arrivals (see Fig 5c). Results for other time windows are similarly ambiguous, showing only weak correlation between the two time series and offering no clear explanation of the surge dynamics.

Therefore, the variability of aircraft arrivals only partially explains the surge dynamics. Other demand sources apart from aircraft arrivals seem unlikely at DCA. While we assumed a constant airplane load (fraction of occupied seats), we expect this factor to vary at most on the timescale of several hours with a typical daily pattern rather than on short timescales like the surge dynamics. Overall, we conclude that changes in ride-hailing demand do not offer a sufficient explanation for the observed surge dynamics. There must be additional, unobserved changes of supply affecting the surge fee.

---

[2] According to the US Federal Communications Commission, "[t]he use of cellular telephones while [..] aircraft [are] airborne is prohibited".

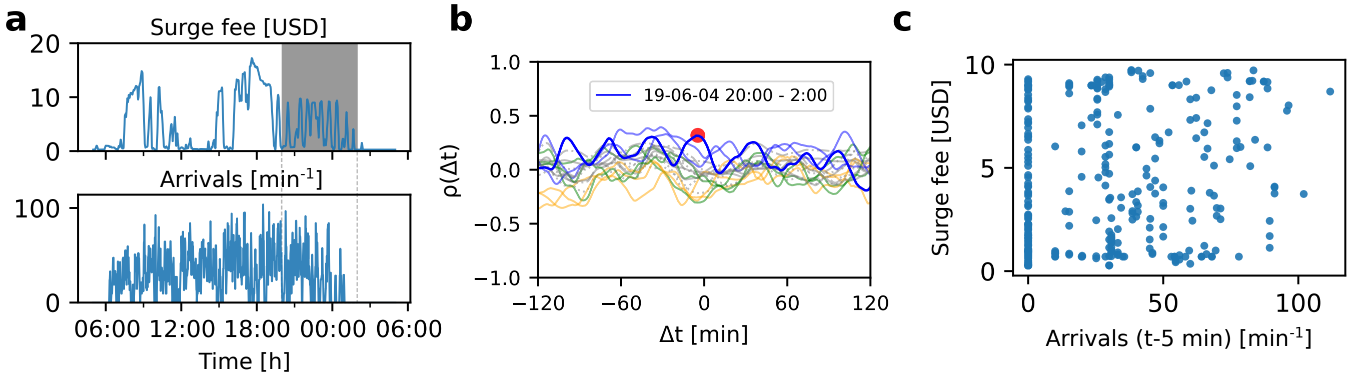

Supplementary Figure 5. **Capacity of arriving aircraft is only weakly correlated with UberX surge fees.** **a** UberX surge fee (top) and capacity of arriving airplanes (bottom) at DCA on Tuesday, 19-06-04. **b** Normalized cross-correlation $\rho(\Delta t)$ between fixed time windows of the surge fee and the capacity of arriving aircraft. The dark blue line corresponds to the shaded window from 20:00 to 02:00 in panel **a**, light blue lines to the same time windows on other days. Yellow lines show the correlation function for the 6-hour time window from 08:00 to 14:00, green lines for the 6-hour time windows from 14:00 to 20:00. Grey lines show 12- and 18-hour windows (dotted line: 08:00 to 20:00.; dotted-dashed line: 14:00 to 02:00; solid: 08:00 to 02:00). The maximum correlation $\rho(\Delta t^*) = 0.31$ for the highlighted time window is obtained at $\Delta t^* = -5$ minutes (red point). **c** Surge fee and aircraft capacity at the time lag Δt^* of maximum correlation (red point in panel **b**). There is no apparent relationship between the arrivals and the surge fee, suggesting a large supply-side influence on the observed surge dynamics.

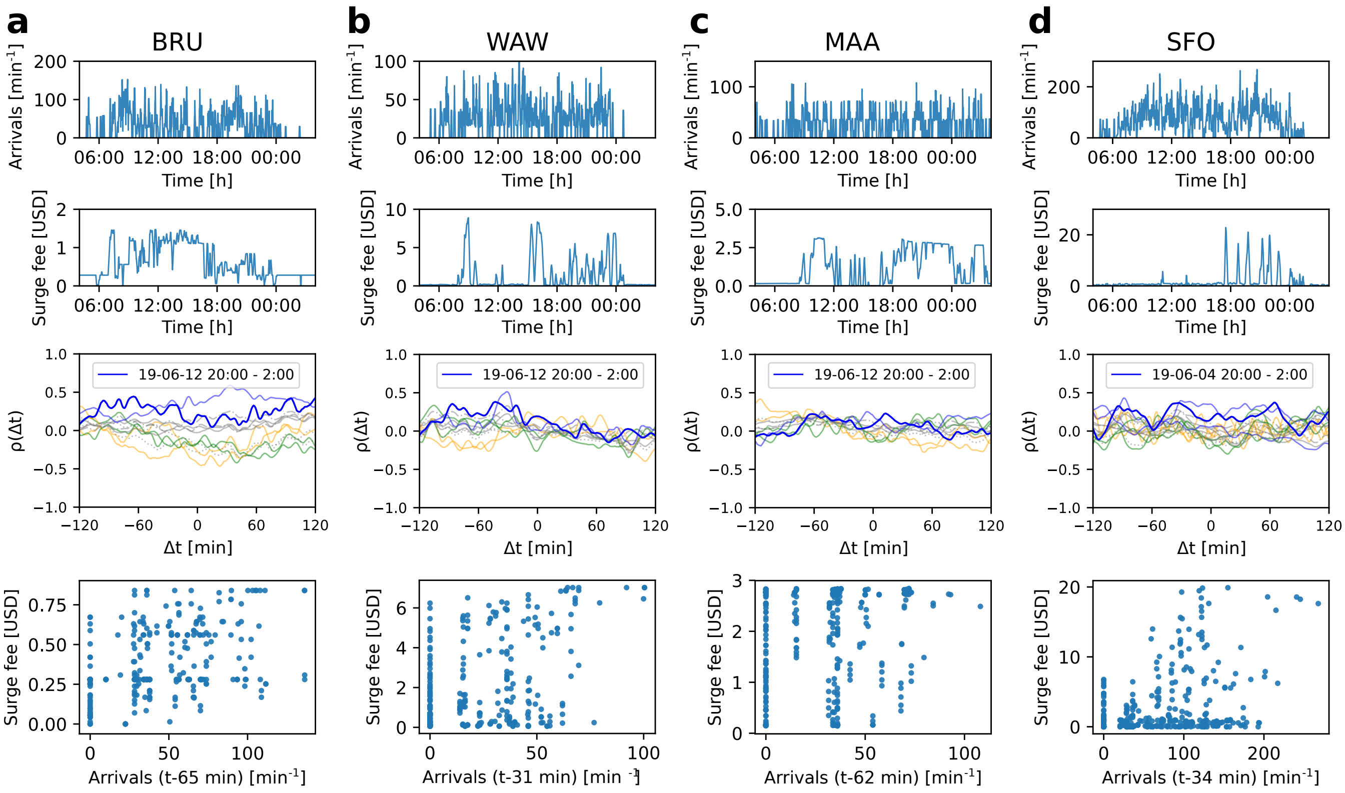

Supplementary Figure 6. **Weak correlations between airport arrivals and surge fee across airports.** The time series of aircraft arrivals and UberX surge fees for **a** Brussels, **b** Warsaw, **c** Chennai and **d** San Francisco (top two rows). The third row shows the cross-correlation (Pearson correlation coefficient) ρ between the surge fee time series and the arrivals at different delays Δt using the same approach as for DCA (compare Supplementary Fig. 5). The bottom row shows the surge fee vs. the number of arrivals plot at the delay Δt^* of the largest cross-correlation. As in Supplementary Fig. 5c, the correlation between arrivals and surge fee is weak most of the time.

Conclusion

The taxi and airport data analyzed above indicate an approximately constant demand during the day with no direct correspondence between demand changes and surge dynamics. Taxi data show a generally higher average demand during the evening, suggesting that price surges may be more likely during this time. Aircraft arrivals show weak correlation with the surge fee, offering only a partial explanation of the surge dynamics.

Unrelated to direct demand fluctuations, we consider other sources for demand and supply changes. Specifically, longer service times during rush hour (compare Supplementary Fig. 3) not only lead to higher trip fees but also to drivers spending more time serving each request. With a constant demand (compare taxi departures in Supplementary Fig. 4), this means more drivers are busy and fewer drivers are available at a given time during this period. This general mechanism for supply shortages as a secondary effect of prolonged heavy traffic is consistent with the observed long duration price surges during main commuting times.

In summary, we only have a satisfactory explanation for the long rush hour peaks of the surge fee at DCA. The short, repeated, almost periodic surges with universal peak value and duration (in particular in the evening between 19:00 and 03:00, see Supplementary Fig. 2, top right) cannot be explained by demand-side fluctuations alone or general demand and traffic conditions. We therefore consider these peaks as supply-side induced (or at least having a strong supply-side influence), consistent with the reported driver-induced price surges at DCA in the evening [1]. In the following, we attempt to find price time series with similar out-of-equilibrium surge dynamics to identify locations that may also be affected by artificially induced price surges.

Supplementary Note 2. Statistical properties of surge pricing

Price surges reflect the imbalance of supply and demand at the origin location at the time of a given request. In the previous section, we have identified patterns of the surge dynamics in DCA that are likely related to artificial supply manipulation (compare DCA, Supplementary Fig. 2) [1]. In the examples presented before, however, we also observe locations without surge activity (compare IAH, Supplementary Fig. 2). Different locations around the globe exhibit different price dynamics, some without any price surges, some with single, distinct surge peaks and others with large, frequent surges similar to those observed in DCA (Supplementary Fig. 7 and 8).

In this section, we attempt to characterize the out-of-equilibrium surge dynamics by quantifying properties of the price surges and the price time series at all observed locations (Supplementary Fig. 1) and aim to identify cities with artificial price surges by comparing their surge dynamics to those observed at DCA.

We approach the problem of identifying price surges and quantifying the surge dynamics of locations from two different directions. First, we attempt to identify individual surge peaks directly. Second, we consider the distribution of the price changes across the whole price time series and identify two distinct contributions to the price dynamics, isolating changes corresponding to the surge fee component of the price changes in cities exhibiting price surges. While we find no clear distinction between locations with and without price surges or with and without potentially artificially induced price surges, we find several locations with similar or more volatile surge dynamics than DCA.

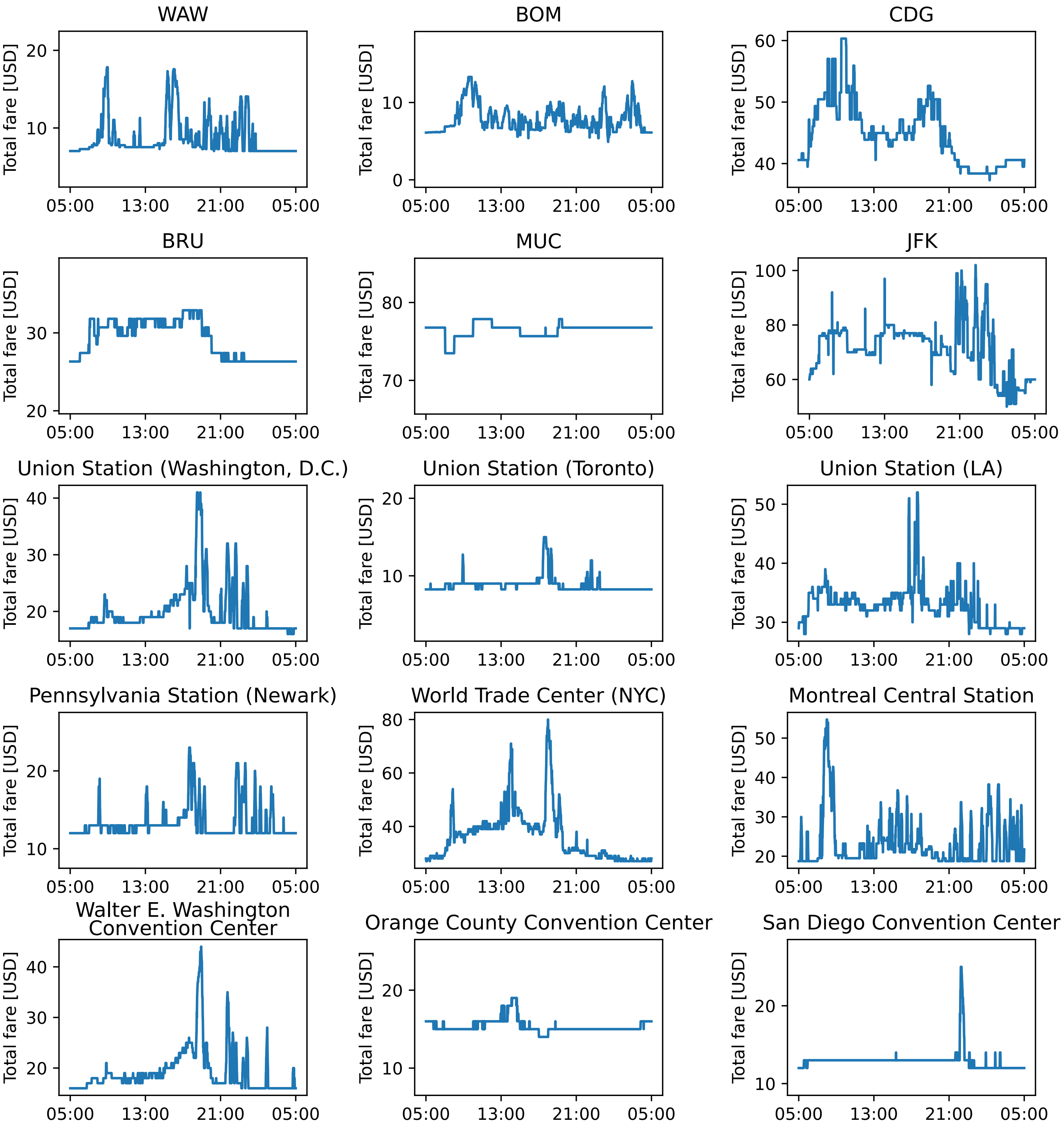

Supplementary Figure 7. **The evolution of total fares for standard UberX services differs qualitatively across locations.** Sample of total fare dynamics over a time span of 24 hours for rides originating from airports (top two rows), train stations (third and fourth row) and convention centers (bottom row) in different cities illustrating the range of possible price dynamics. Trip characteristics can be found in the data section and in Supplementary Tab. II. All currencies were converted to USD using the exchange rates for the respective day provided by the European Central Bank.

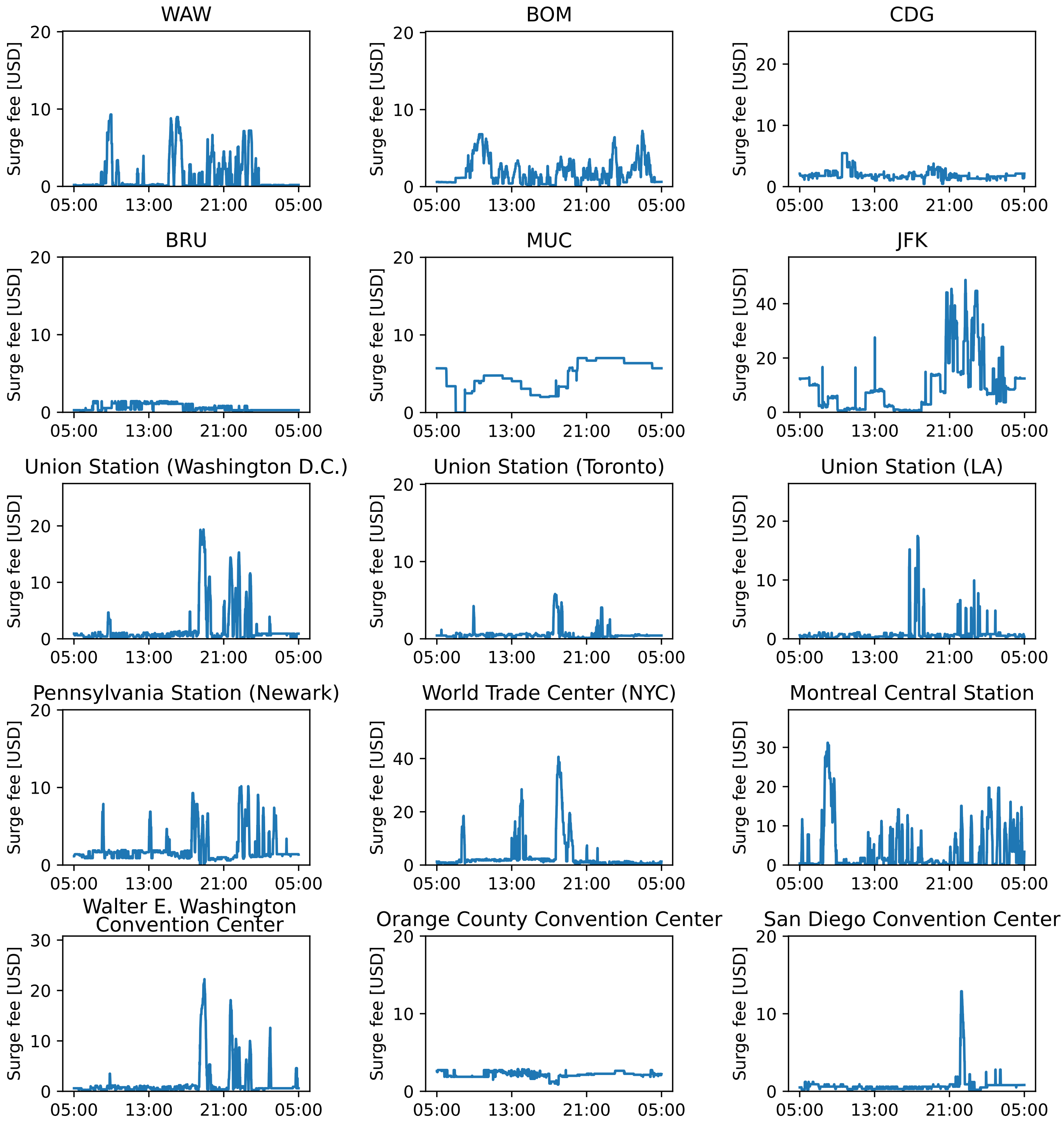

Supplementary Figure 8. **The evolution of surge fees for standard UberX services differs qualitatively across locations.** Estimated surge fee for the trip samples shown in Supplementary Fig. 7. The surge fee shows a similarly broad range of dynamics as the total fare. All currencies were converted to USD using the exchange rates for the respective day provided by the European Central Bank.

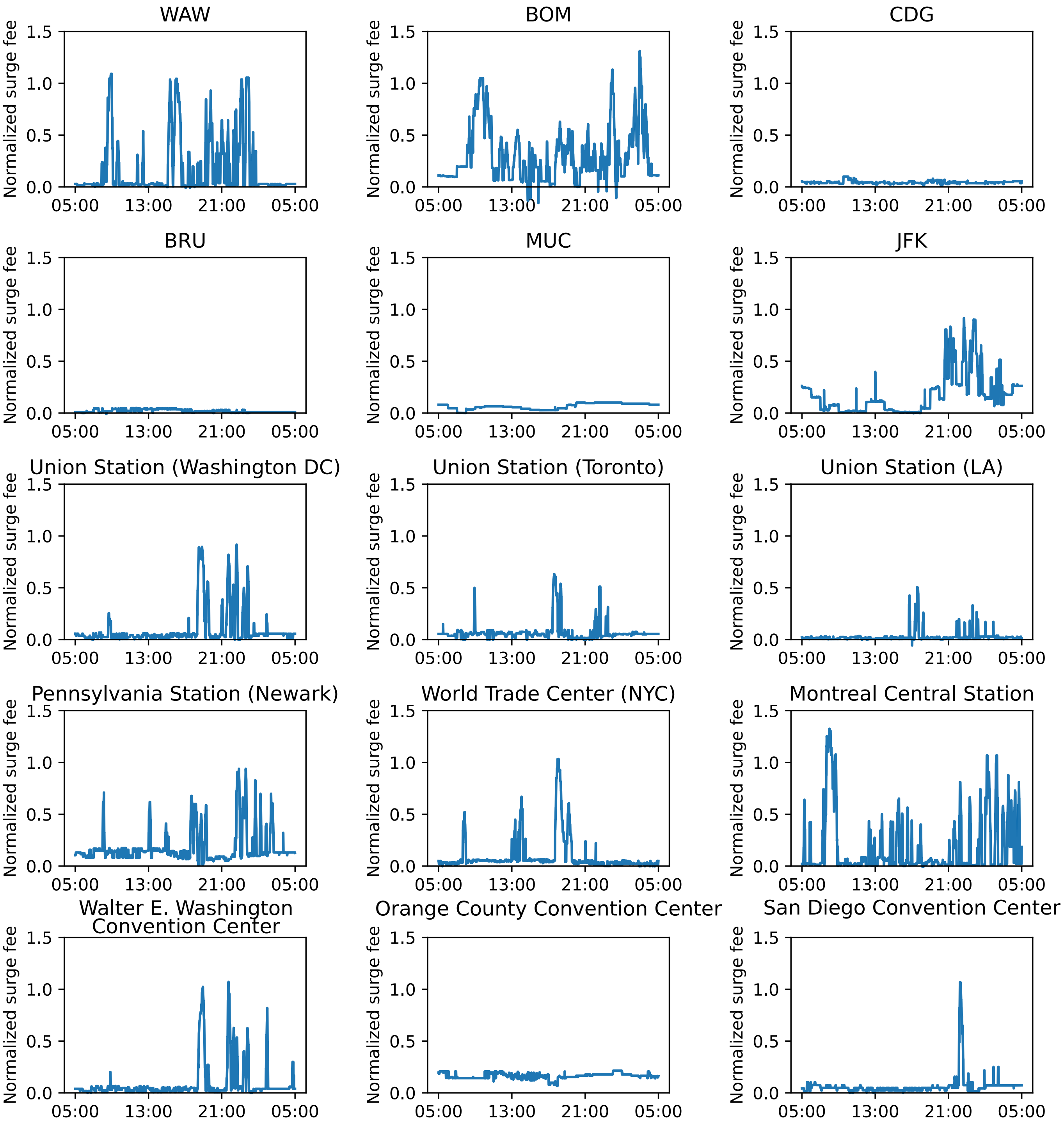

Supplementary Figure 9. **The normalized surge fees for standard UberX services enables comparison of the surge dynamics across locations.** Estimated normalized surge fee (surge fee divided by base cost) for the trip samples shown in Supplementary Fig. 7. The normalized surge fee effectively represents a surge factor. Using this representation, the dynamics at different locations become comparable independent of the absolute cost of the trip (e.g. trip distance or local currency). In particular, differences between locations with and without price surges become clearer.

Statistics of individual surge peaks

In order to identify individual surge peaks, we consider the surge fee component $S_{\mathrm{X}}(t)$ of the total UberX fare (compare Supplementary Fig. 8). To compare different locations with varying total fare, we normalize the surge fee by the base cost at the current time, resulting in a surge factor $s_{\mathrm{X}}(t)$ describing the relative increase of the price due to price surges (compare Supplementary Fig. 9). In order to filter surges due to overall demand increase, we subtract the corresponding premium service surge factor s_{prem} (e.g. of Uber Black), resulting in a normalized surge factor

$$\tilde{s}_{\mathrm{X}} = \frac{\text{surge fee}_{\mathrm{UberX}}(t)}{\text{base cost}_{\mathrm{UberX}}(t)} - \frac{\text{surge fee}_{\mathrm{Black}}(t)}{\text{base cost}_{\mathrm{Black}}(t)} \,. \tag{S3}$$

We use the premium products as a reference value since they tend to exhibit price surges only on rare occasions, presumably at times of generally high demand.

Using this normalized surge factor, we define a surge by values $\tilde{s}_{\mathrm{X}} > 0.2$, that means a surge starts when $\tilde{s}_{\mathrm{X}}$ increases above 0.2. To avoid peaks repeatedly ending and beginning due to small fluctuations, we define the end of a surge as the first time $\tilde{s}_{\mathrm{X}}$ decreases again to below 0.1. Using different (reasonable) threshold values gives qualitatively similar results. These peaks are well described by boxes with start and end times defined by the peak identification conditions above and height given by the maximum value of the normalized surge factor during the peak. An example is illustrated in Supplementary Fig. 10.

We use the identified surges to compute aggregate characteristics of the surge dynamics for each location (see data section and Supplementary Tab. II for details). In particular we consider the mean duration and height of the peaks as well as the average number of peaks. Supplementary Figure 11a-c shows the resulting statistics for each city by rank. The distinction between locations with and without peaks is clear only in the average peak height (Supplementary Fig. 11b) due to the non-zero threshold used for peak identification. When peaks were identified at a location, neither the average duration, height nor number of peaks clearly separate locations with few or many or with strong or weak price surges. All measures change steadily across the different locations. Also the combination of these features (see Supplementary Fig. 11d for an example) does not reveal distinct groups of locations.

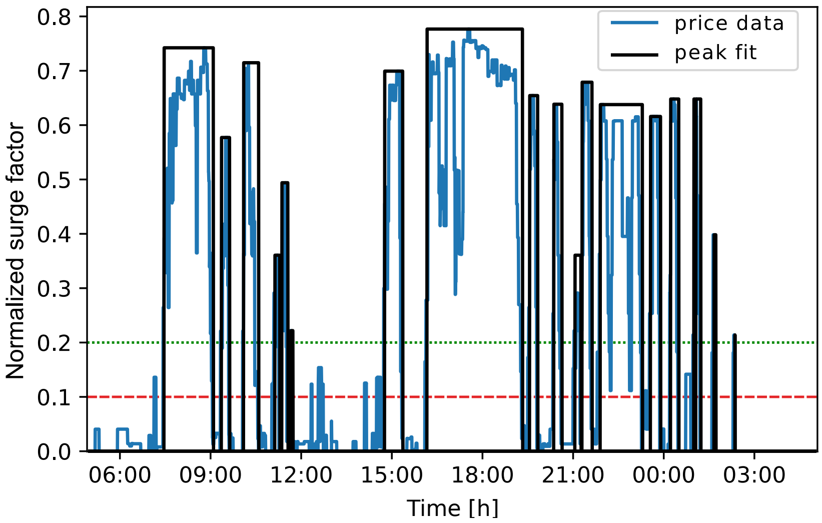

Supplementary Figure 10. **Surge peak detection.** Visualization of the surge peak identification for the normalized surge factor $\tilde{s}_{\mathrm{X}}$, Eq. (S3, in DCA. Peaks are defined to start when $\tilde{s}_{\mathrm{X}} > 0.2$ (dotted green line) and end when $\tilde{s}_{\mathrm{X}} < 0.1$ (dashed red line). Their amplitude is taken as the maximum value of $\tilde{s}_{\mathrm{X}}$ during the peak (solid black rectangles).

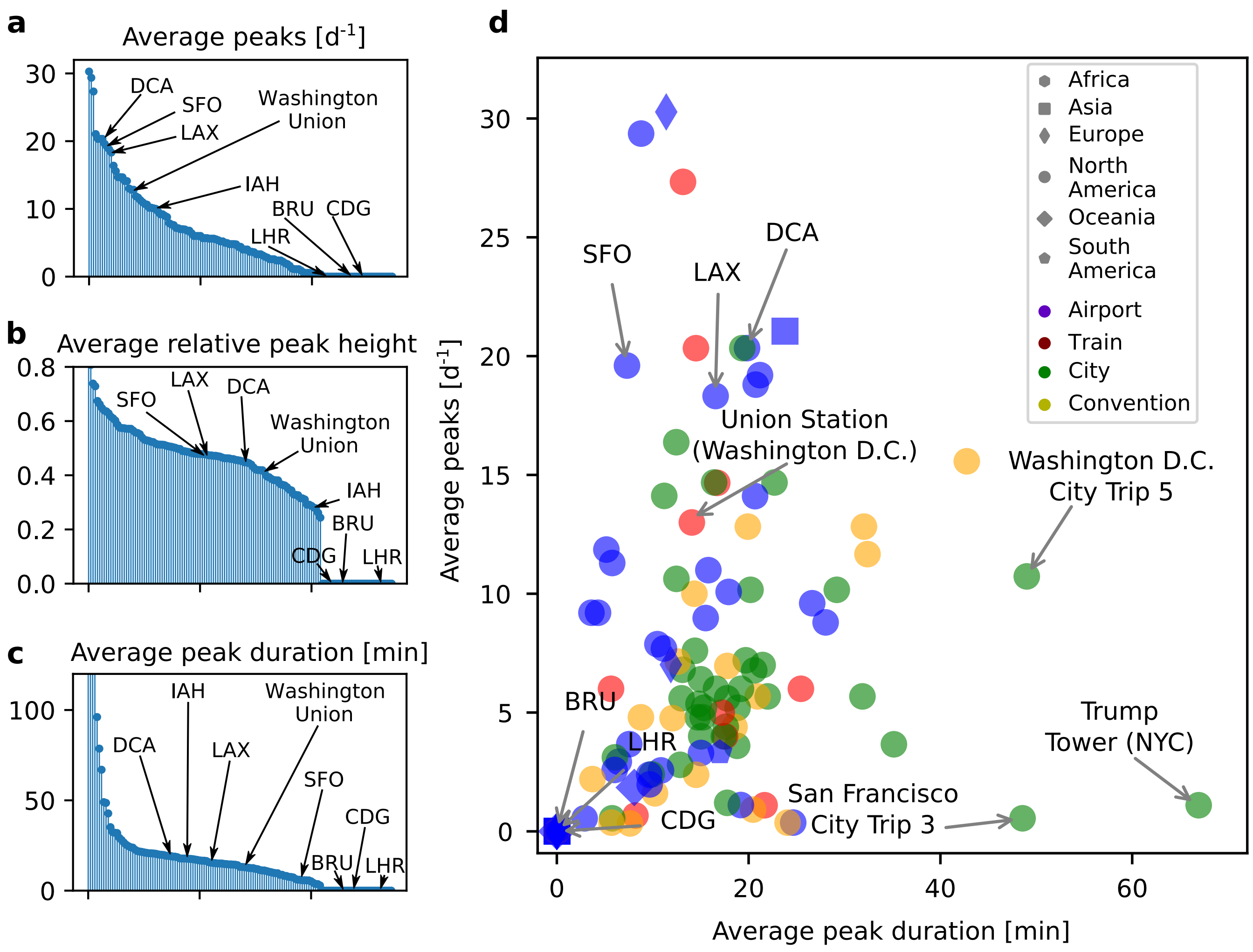

Supplementary Figure 11. **Single peak statistics do not separate locations with qualitatively different surge dynamics.** None of the average peak characteristics (**a** number of peaks, **b** height, **c** duration] clearly classifies the cities with price surges into different categories, all measures gradually change from high to low values. The measures only distinguish between cities with peaks from those where no peaks where identified (e.g. panel **b** due to the threshold $\tilde{s}_X = 0.2$ used for peak identification). **d** Combining the average peak count per day and the average peak duration also reveals no clear pattern (results are qualitatively the same for other combinations of observables). Some locations with extremely long lasting surges (compare panel **c**) with an average peak duration upwards of 80 minutes are not visible in **d** (Orange County Convention Center, San Francisco City Trip 5, San Francisco City Trip 9, Minneapolis Convention Center). The long lasting price surges suggest a general supply-demand imbalance in these locations rather than artificially induced price surges, similar to the sustained rush-hour surge peaks in DCA (compare Supplementary Fig. 2). No dependence on the trip type (airport, station, city or convention) is visible.

Statistics of surge time series

In order to better characterize at which locations repeated price surges occur, we consider statistics of the entire price time series instead of individual price surge peaks. In particular, we consider per minute changes in the normalized price to identify the typical timescales of the price dynamics.

For the analysis described in this section, we use a smoothed time series of normalized prices, i.e. the total fare divided by base cost, measuring the price in fractions of the time dependent base cost with equally spaced data points every minute (see also data processing Supplementary Note 4). For these normalized time series, we compute the per minute changes Δp between consecutive time points,

$$\begin{aligned}\Delta p(t) &= \frac{\text{total fare}(t)}{\text{base cost}(t)} - \frac{\text{total fare}(t-1)}{\text{base cost}(t-1)} \\ &= \frac{\text{base cost}(t) + \text{surge fee}(t)}{\text{base cost}(t)} - \frac{\text{base cost}(t-1) + \text{surge fee}(t-1)}{\text{base cost}(t-1)}\,. \\ &= \frac{\text{surge fee}(t)}{\text{base cost}(t)} - \frac{\text{surge fee}(t-1)}{\text{base cost}(t-1)}\,.\end{aligned} \tag{S4}$$

The normalized price can be interpreted as an effective surge factor (compare previous section). Although Uber began to transition to an additive surge computation [6, 8], this normalization is necessary to compare price estimates across different locations regardless of base cost, currency or sampling frequency.

Note that even in locations without surge activity, the estimated surge fee is not exactly zero, likely due to rounding of the price estimates. Taking into account these small fluctuations, we expect there to be three major contributions to the relative price changes: (i) Minutes without any change, e.g. during night where traffic and demand conditions do not change at all. (ii) Small changes due the rounding of the price estimate and slow changes of the base cost, mostly driven by changes of the trip fare as traffic conditions change over multiple hours. (iii) Fast changes of the surge fee component, increasing the price by up to 80% in a matter of minutes.

In order to quantify the contribution of these three parts, we fit an extended Gaussian mixture model to the data, consisting of one Dirac-delta distribution and two Gaussian distributions modelling the three contributions described above. We take the mean of both Gaussians to be zero (no price change on average), such that

$$\text{Prob}\,(\Delta p) = w_0\,\delta(\Delta p) + w_{\text{base}}\,\frac{1}{\sqrt{2\pi\sigma^2_{\text{base}}}}\,e^{-\frac{\Delta p^2}{2\,\sigma^2_{\text{base}}}} + w_{\text{surge}}\,\frac{1}{\sqrt{2\pi\sigma^2_{\text{surge}}}}\,e^{-\frac{\Delta p^2}{2\,\sigma^2_{\text{surge}}}} \tag{S5}$$

where we define the second Gaussian corresponding to the surge fee component to be broader, $\sigma_{\text{surge}} > \sigma_{\text{base}}$. We take all datapoints with $\Delta p^2 < 10^{-7}$ to belong to the Dirac-delta distribution indicating no price change and fit the two Gaussian distributions to the remaining data to determine the weights and standard deviations. A broad distribution corresponds to a fast changing behaviour (surge fee), while a narrow distribution describes a slowly changing price (base cost). For better visibility we only show the reduced data without the data points with no price change in the following, corresponding to the two Gaussian distributions without the Dirac delta distribution.

Supplementary Fig. 12 shows the resulting distribution of the price changes at different locations without the Dirac-delta distribution. As expected, for all locations we find a large number of small changes, corresponding to small fluctuations of the base cost and rounding errors of the price estimates. Locations exhibiting price surges, like DCA, SFO and LAX, additionally have many larger price changes, corresponding to the dynamics of the surge fee. In contrast, locations without surge activity (LHR, CDG, BRU) have a very narrow distribution.

For the data shown in Supplementary Fig. 12, we find base cost fuctuations characterized by $\sigma_{\text{base}} \approx 0.003$ whereas the relative price changes corresponding to the surge fee component (when they are different from the vase cost fluctuations) are characterized by $\sigma_{\text{surge}} \approx 0.05$. When price surges do not exist, the relative price change distribution is typically well described by a single Gaussian such that the estimated values for σ_{base} and σ_{surge} are more similar.

To validate these observations, we compute the relative price changes also for Uber Black at Reagan National Airport (DCA) in Washington D.C. Comparing the resulting distributions for UberX and Uber Black (see Supplementary Fig. 13) shows that changes of the Uber Black price are well described by only the base cost fluctuations. This is consistent with the observation that the trip fare evolves synchronously for UberX and Uber Black but only UberX exhibits significant surge activity (compare Supplementary Fig. 2 above).

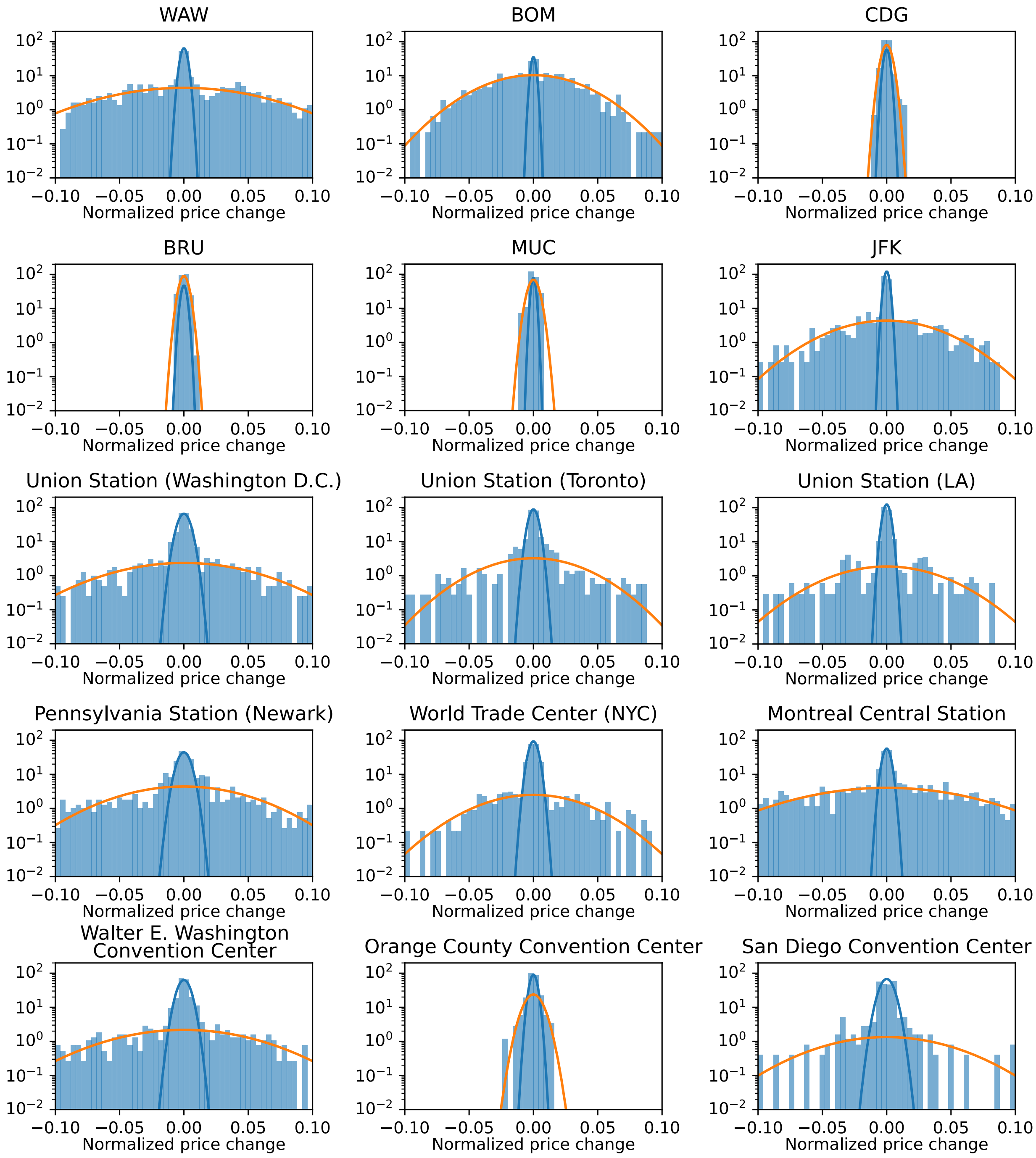

Supplementary Figure 12. **Relative price change distributions differ between locations with and without surge activity.** The relative price change distribution from the normalized UberX price time series for different locations (blue histogram, see text). All locations show a narrow peak corresponding to slow changes of the base cost and small rounding errors of the price estimate (base cost fluctuations, blue line). Locations where price surges are prevalent (e.g. WAW, BOM in the top row) show a second part of the distribution, characterizing the larger and faster price changes during price surges (orange line). The orange and blue lines indicate the individual distributions of the Gaussian mixture model fit Eq. (S5).

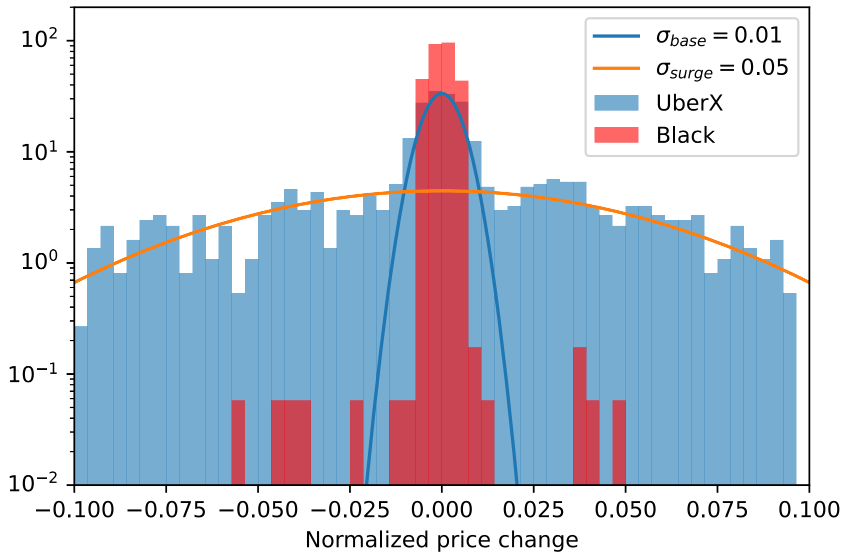

Supplementary Figure 13. **Uber Black price changes are well described by only base cost fluctuations.** Distribution of the relative price changes at the Reagan National Airport (DCA) for UberX (blue) and Uber Black (red, higher values due to normalization of he probability distribution). The Gaussian distribution corresponding to the base cost fluctuations of UberX approximately matches the price dynamics of Uber Black, illustrating that these are base cost fluctuations unrelated to surge activity.

Based on these observations, we attempt to quantify the surge dynamics at different locations. If the estimated σ_{surge} is large, the price time series of that location likely exhibits price surges. The absolute value of σ_{surge} characterizes the strength of the surges, the weight w_{surge} quantifies the overall contribution of surge dynamics to the price behaviour, similar to the average number of surge peaks in the previous section. Note that in locations without surge activity, the estimated standard deviation of both distributions is similar and the weights are becoming more and more interchangeable. In the limit of $\sigma_{\text{main}} = \sigma_{\text{surge}}$, all combinations of weights are indistinguishable.

Supplementary Figure 14 shows a scatter-plot of the time series statistics illustrating the magnitude σ_{surge} (normalized surge strength, large values indicate strong price surges, small values indicate no surge activity) and the weight w_{surge} (surge contribution, large values of w_{surge} indicate many price surges, small values indicate few surges). Also in this representation, the data do not separate into distinct clusters. However, we clearly identify several locations where both σ_{surge} and w_{surge} are large (top right in Supplementary Fig. 14), including DCA (Washington D.C.), LAX (Los Angeles), WAW (Warsaw) and Pennsylvania Station (Manhattan, New York City). Prices in these locations show strong, highly volative surge activity similar to DCA, suggesting artificial causes for at least some of the price surges observed there.

We also find a large number of locations with a broad distribution, $\sigma_{\text{surge}} \geq 0.03$, but small weight $w_{\text{surge}} \leq 0.3$, including SFO (San Francisco) and most city trips in Manhattan, San Francisco and Washington, D.C.. These locations typically exhibit strong but infrequent price surges, some of which may be induced artificially. In particular for city trips, frequently organizing artificial surges is more difficult due to no central location where all drivers meet. Some of these locations may also exhibit a few long duration surge peaks caused by periods of high traffic and congestion or be affected by price surges at close by airports or other points of interest (e.g. city trips in the same city).

Interestingly, all 12 observed train station trips exhibit relatively large $\sigma_{\text{surge}} \geq 0.03$. Here, it is plausible that arriving trains cause larger increases in demand, as trains can carry more passengers than planes and the time to leave the train and book a ride is likely more homogeneous than for airplane travelers. While this higher demand increases the incentive to induce a price surge (see game theoretic models below), it also offers a plausible demand-side explanation for the price surges in these locations.

Finally, locations with $\sigma_{\text{surge}} \leq 0.02$ either do not exhibit price surges at all or only show minor price changes due to surge pricing. At these locations, including BRU (Brussels), CDG (Paris) and LHR (London), no (artificial) price surges are induced. These include a larger fraction of non-US locations (relative to all observed locations), likely due to different local regulations legislating surge pricing and ride-hailing services. The value of w_{surge} is less meaningful for these locations, as in many cases $\sigma_{\text{surge}} \approx \sigma_{\text{base}}$ and both distributions in the mixture model are very similar.

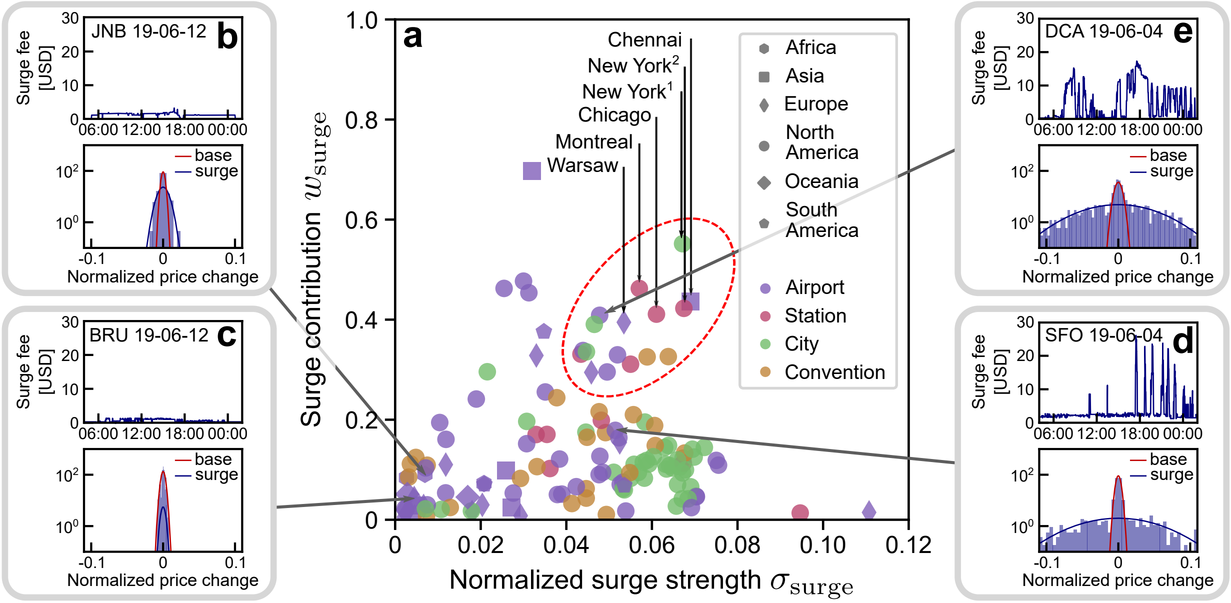

Supplementary Figure 14. **Price change distributions characterize the surge activity in different locations. a** The main panel illustrates the weight w_{surge} (surge contribution) and the width σ_{surge} (normalized surge strength) of the broader distribution corresponding to the surge fee in the Gaussian mixture model fit [Eq. (S5)] for all observed locations. No clear classification of the locations is possible. However, we can distinguish three general regions. **b** and **c**, Small values of σ_{surge} indicate no surge activity as observed for example in JNB and BRU. Here, the distribution of relative price changes is well described by a single Gaussian corresponding to the base fare changes. On the other hand, large values of σ_{surge} signify fast and large price surges. **d**, If w_{surge} is small, the surges are infrequent (compare panel for SFO). **e**, If w_{surge} is also large, these price surges occur frequently, as shown for DCA. Several location (red ellipse) share similar characteristics of the price change distribution.

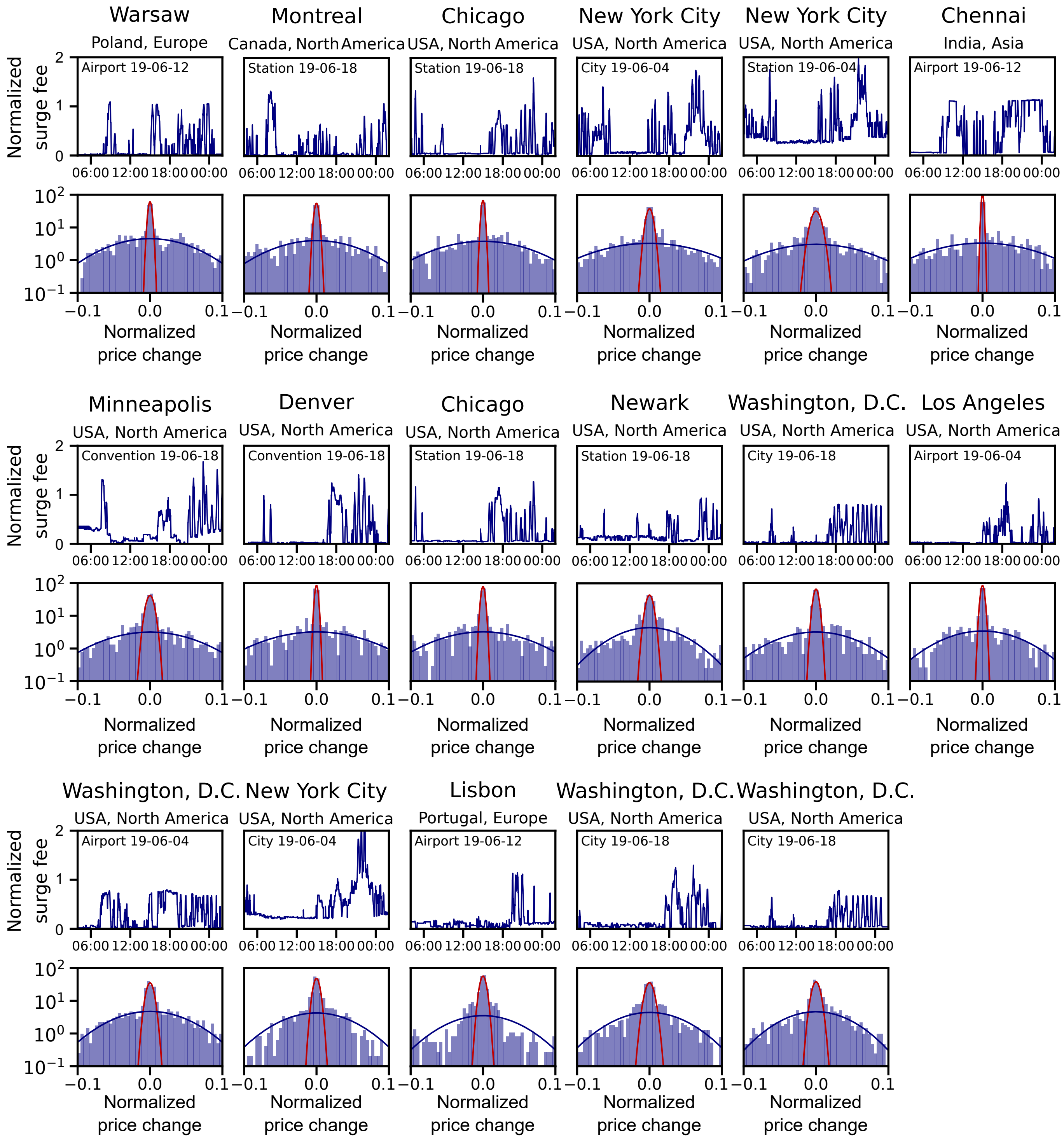

Supplementary Figure 15. **Identifying non-equilibrium surge dynamics and anomalous supply dynamics**. Surge time series and price change statistics of all locations identified in Supplementary Fig. 14 (red ellipse) that we expect to exhibit anomalous surge behaviour (compare also Fig. 4 in the main manuscript). Almost all identified locations show strong and frequent price surges. Exceptions are only Los Angeles (middle row, right) and Lisbon (bottom row, middle).

Supplementary Note 3. Incentive structure for drivers under dynamic pricing

By introducing dynamically adjustable prices, ride-hailing service providers create mobility services that, in principle, feature a self-organized equilibration of spatio-temporal demand and supply imbalances. Two groups of agents interact in such systems: customers and drivers. While customers have demand for mobility services, drivers offer such services against financial compensation. Price elasticities of demand and supply determine the equilibrium price point. Here, we illustrate in detail the fundamental incentive structure in dynamics pricing for drivers and how it may promote artificial supply shortages. First, we provide the simplest possible game-theoretical model revealing demand and supply constellations under which drivers collectively stimulate supply shortages. Second, we show that it is socially desirable for groups of drivers to coordinate in a broad regime of demand elasticities. Third, we reproduce the qualitative surge dynamics induced by collective action among drivers in a simple dynamic model with stochastic passenger arrivals, including the dynamics shown in Fig. 2 of the main manuscript.

Two-player game

Two-player game with inelastic demand

Consider a setting where the demand D for rides is inelastic and fixed. Customers are insensitive to dynamic price changes and always agree to pay the current total fare to accommodate their demand. Such an assumption may be justified for constellations where business travelers have an urgent need to complete a trip in time, e.g. not to miss a business meeting. Typically, employers reimburse their employees for the cost of travel. Hence, business travelers have no incentive to prioritize their mobility demand based on the current price structure but aim for making it on time to their business meeting irrespective of the financial cost of mobility.

Drivers may benefit from such constellations in terms of higher payoffs if they manage to stimulate dynamic price increases by creating an imbalance between demand and supply. Clearly, drivers can only affect the supply, i.e., increase the price by causing supply shortages, not the demand. Specifically, drivers can choose to switch OFF their ride-hailing mobile application to make their service temporarily unavailable. Thereby, they decrease the (observable) local supply S while demand D stays constant, making dynamic pricing algorithms increase the price per ride. If drivers switch their mobile application ON again and accept a customer's trip request before the dynamic pricing algorithm readjusts trip fares, drivers may secure higher payoffs. Note that the actually available supply of drivers (e.g. the total number of drivers idling at the airport) never changes, the dynamic pricing algorithm only reacts to an apparent supply shortage.

If $D \geq S$ all drivers benefit from this strategy. If $D < S$, drivers playing an OFF-strategy risk that other drivers remain ONline. These ON-players may exploit their first mover advantage in securing a ride and reduce the remaining demand for OFF-players. Hence, drivers play a game about cooperation and defection, where cooperation corresponds to the OFF-strategy and defection is the ON-strategy. Depending on price, supply and demand, the game undergoes a transition from stag hunt or prisoner's dilemma to a trivial game promoting the creation of artificial supply shortages.

We capture this idea in the following two-player game with strategies ON/OFF for which the drivers' payoff structure is given by

	ON	OFF
ON	$(\frac{D}{2}p_{\text{low}}, \frac{D}{2}p_{\text{low}})$	$(p_{\text{mid}}, (D-1)p_{\text{mid}})$
OFF	$((D-1)p_{\text{mid}}, p_{\text{mid}})$	$(\frac{D}{2}p_{\text{high}}, \frac{D}{2}p_{\text{high}})$

with $p_{\text{low}} \leq p_{\text{mid}} \leq p_{\text{high}}$ modelling dynamic pricing as the apparent supply changes. To avoid case distinctions (each driver can serve a minimum of zero and a maximum of one customer), we assume that the demand D is in the interval $1 \leq D \leq 2$. The expected payoffs shown in the payoff matrix assume a uniform probability to secure a ride from the currently available trip demand across currently available drivers. For example, if both drivers remain ON their respective payoff Π_1, Π_2 is

$$\Pi_1(\text{ON}|\text{ON}) = \frac{D}{2}\,p_{\text{low}}$$
$$\Pi_2(\text{ON}|\text{ON}) = \frac{D}{2}\,p_{\text{low}}\,, \tag{S6}$$

where $D/2 = D/S$ is the probability of securing a ride at the respective instance in time. However, if one of the two

switches their app OFF and the other remains ON, the probability of getting a ride shifts. The driver who remains ON has a first mover advantage because none of the demand have been served yet. The demand $D > 1$ meets a current supply of $S - 1 = 1$ and the dynamic pricing algorithm increases the price for a ride to p_{mid}. At the same time, the ON-players probability of getting a ride increases because there is no competition from the OFF-player at the moment. With $D \geq 1$, a lone ON-player will always secure a ride. The OFF-player has to pick a ride from the left-over demand $D - 1 \leq D/2$, reducing their chance to serve a customer. Consequently, the expected payoff is

$$\begin{aligned} \Pi_1(\text{ON}|\text{OFF}) &= p_{\text{mid}} \\ \Pi_2(\text{OFF}|\text{ON}) &= (D-1)\, p_{\text{mid}} \, . \end{aligned} \tag{S7}$$

If both players play OFF, they secure an even higher price $p_{\text{high}} > p_{\text{mid}}$ as the dynamic pricing algorithm reacts to the apparent supply $S - 2 = 0$. In this case, both players again have equal chance to secure a ride and achieve the expected payoff

$$\begin{aligned} \Pi_1(\text{OFF}|\text{OFF}) &= \frac{D}{2}\, p_{\text{high}} \\ \Pi_2(\text{OFF}|\text{OFF}) &= \frac{D}{2}\, p_{\text{high}} \, . \end{aligned} \tag{S8}$$

The Nash equilibria of the two-player game depend on the parameter values of $D, p_{\text{low}}, p_{\text{mid}}$ and p_{high}. In particular, we distinguish two extreme cases:

- $\mathbf{D = 1}$: In the low demand limit $D = 1$, the game is either a stag hunt or a prisoner's dilemma.

	ON	OFF
ON	$\left(\frac{1}{2}p_{\text{low}}, \frac{1}{2}p_{\text{low}}\right)$	$(p_{\text{mid}}, 0)$
OFF	$(0, p_{\text{mid}})$	$\left(\frac{1}{2}p_{\text{high}}, \frac{1}{2}p_{\text{high}}\right)$

  – Stag Hunt: For $p_{\text{mid}} < \frac{p_{\text{high}}}{2}$, the payoff matrix reduces to that of a stag hunt [11] with $0 < \frac{1}{2}p_{\text{low}} < p_{\text{mid}} < \frac{p_{\text{high}}}{2}$, where OFF is a payoff dominant, and ON a risk dominant Nash equilibrium.

  – Prisoner's Dilemma: For $p_{\text{mid}} > \frac{p_{\text{high}}}{2}$, the payoff structure changes to that of a prisoner's dilemma [12], $0 < \frac{1}{2}p_{\text{low}} < \frac{p_{\text{high}}}{2} < p_{\text{mid}}$, where ON is the unique Nash equilibrium.

- $\mathbf{D = 2}$: In the high demand limit $D = 2$, the payoff matrix is a trivial, fully symmetric game

	ON	OFF
ON	$(p_{\text{low}}, p_{\text{low}})$	$(p_{\text{mid}}, p_{\text{mid}})$
OFF	$(p_{\text{mid}}, p_{\text{mid}})$	$(p_{\text{high}}, p_{\text{high}})$

  where OFF is the strictly dominant Nash equilibrium. Drivers always have an incentive to coordinate to cause a price surge in lack of competition for rides.

Hence, the dynamic price response and the demand to supply ratio govern the drivers' incentives under which constellations to switch OFF their mobile applications or when to remain ON.

Two-player game with elastic demand

Based on the simplified game modelling the fundamental incentives for drivers in ride-hailing systems described in the previous section, we now consider in more detail under which conditions drivers induce artificial price surges. In particular, we relax the assumption of inelastic demand D, such that only some customers $D' \leq D$ actually book a ride.

For the following analysis, we assume a linear increase of the surge fee as the supply decreases such that the total fare p' is given by

$$p'(S < D) = p_{\text{base}} + p_{\text{surge}}^{\text{max}} \frac{D - S}{D} \,. \tag{S9}$$

The price increases beyond the constant base cost p_{base} as soon as the supply S falls below the total demand D (all potential customers checking the app for the cost of a ride). Without loss of generality, we assume $p_{\text{base}} = 1$ in all following calculations for the two-player game. The dependence of the total fare on the supply is illustrated in Supplementary Fig. 16a.

Rather than paying high surge fees, however, customers may choose to wait until the price for their trip decreases or they may choose alternative modes of transportation such as (fixed price) taxi cabs or public transport to accommodate their demand. We model this price elasticity of the demand by assuming a willingness of customers to pay for a ride uniformly distributed in the interval $[p_{\text{base}}, p_{\text{max}}]$. As the price increases, the number of customers D' actually booking a ride thus decreases linearly as

$$D'(p') = D_0 \left(1 - \frac{p' - p_{\text{base}}}{p_{\text{max}} - p_{\text{base}}}\right) = D_0 \left(1 - \frac{p' - p_{\text{base}}}{p_{\text{max}} - p_{\text{base}}}\right) = D_0(1 - \delta(p' - p_{\text{base}})) \tag{S10}$$

where $\delta = (p_{\text{max}} - p_{\text{base}})^{-1}$ describes the price elasticity of demand. Here, we assume that the base costs are set at the maximum possible value where all customers book a ride if there is no surge (Supplementary Fig. 16b). Additionally, we assume that $p_{\text{high}} = p_{\text{base}} + p_{\text{surge}}^{\text{max}} \leq p_{\text{max}}$ such that there are some customers willing to pay the highest possible surge fee. Otherwise, the surge fee is chosen unreasonably high as it might completely nullify the demand, even if some supply of drivers is still available.

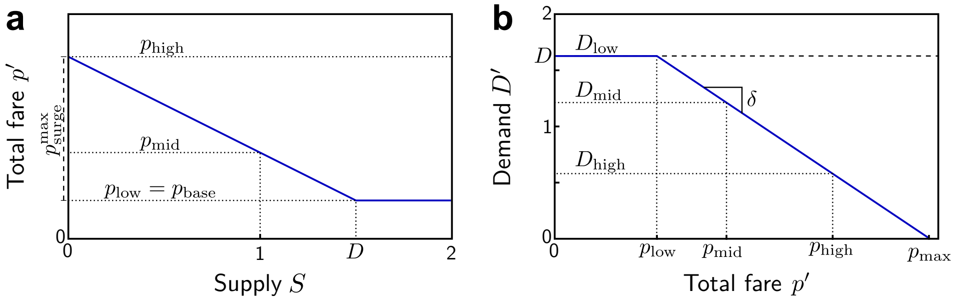

Supplementary Figure 16. **Price structure and demand response in the two-player game. a**, The total fare payed by the customers and earned by the drivers is the base cost p_{base} and an additional surge fee. When the supply decreases below the demand D, the price increases linearly to a maximum value $p_{\text{high}} = p_{\text{base}} + p_{\text{surge}}^{\text{max}}$ [compare Eq. (S9)]. **b**, The demand D' of customers actually ordering a ride decreases as soon as the price increases above p_{base}. It decreases to $D' = 0$ when the price reaches p_{max} [compare Eq. (S10)]. We assume $p_{\text{high}} \leq p_{\text{max}}$ as otherwise the demand would vanish while there are still drivers available, indicating an unreasonably large value of $p_{\text{surge}}^{\text{max}}$.

In summary, for a total supply of $S_{\text{tot}} = 2$ players, we have $S \in \{0, 1, 2\}$ depending on the drivers' choice of strategy with corresponding values $p' \in \{p_{\text{low}}, p_{\text{mid}}, p_{\text{high}}\}$ for the total fare and $D' \in \{D_{\text{low}}, D_{\text{mid}}, D_{\text{high}}\}$ for the demand (Supplementary Fig. 16). The payoff matrix is then given by

	ON	OFF
ON	$\left(\frac{D_{\text{low}}}{2}\, p_{\text{low}},\ \frac{D_{\text{low}}}{2}\, p_{\text{low}}\right)$	$\left(p_{\text{mid}},\ (D_{\text{mid}} - 1)\, p_{\text{mid}}\right)$
OFF	$\left((D_{\text{mid}} - 1)\, p_{\text{mid}},\ p_{\text{mid}}\right)$	$\left(\frac{D_{\text{high}}}{2}\, p_{\text{high}},\ \frac{D_{\text{high}}}{2}\, p_{\text{high}}\right)$

with the total fare and demand depending on the drivers' decisions

- (ON, ON) with supply $S = 2$

$$\begin{aligned} p_{\text{low}} &= p_{\text{base}} \\ D_{\text{low}} &= D \end{aligned}$$

- (ON, OFF) or (OFF, ON) with supply $S = 1$

$$\begin{aligned} p_{\text{mid}} &= p_{\text{base}} + p_{\text{surge}}^{\text{max}} \frac{D-1}{D} \\ D_{\text{mid}} &= D \left(1 - \delta\, p_{\text{surge}}^{\text{max}} \frac{D-1}{D}\right) \end{aligned}$$

- (OFF, OFF) with supply $S = 0$

$$\begin{aligned} p_{\text{high}} &= p_{\text{base}} + p_{\text{surge}}^{\text{max}} \\ D_{\text{high}} &= D \left(1 - \delta\, p_{\text{surge}}^{\text{max}}\right) \end{aligned}$$

where, for simplicity of presentation, we do not explicitly note the case distinctions to ensure that each driver serves at most one and at least zero customers and the total fare only increases if $S < D$. The equations as presented are valid for $1 \leq D \leq 2$ and $\delta\, p_{\text{surge}}^{\text{max}} < 1$.

Supplementary Figure 17 shows the resulting Nash equilibria for this two-player game with elastic demand for different demand D, maximum surge fee $p_{\mathrm{surge}}^{\mathrm{max}}$ and demand elasticities δ (compare Fig. 2b in the manuscript).

- $\delta = 0$: In the limit of inelastic demand we reproduce the findings discussed above. For demand $D \leq 2$ and $p_{\mathrm{mid}} > p_{\mathrm{high}}/2$ strong competition between players results in a Nash equilibrium in ON-strategies (green in Supplementary Fig. 17a). For sufficiently high surge fees the game changes to a stag hunt ($p_{\mathrm{mid}} > p_{\mathrm{high}}/2$) where both ON, OFF and mixed strategy Nash equilibria coexist (striped area in Supplementary Fig. 17a). For sufficiently high demand (guaranteed for $D \geq 2$), drivers are strongly incentivized to switch OFF their apps (orange in Supplementary Fig. 17a).
- $\delta = 0.15$: The drivers' incentive structure starts to shift when introducing demand elasticitiy (see Supplementary Fig. 17b). Inducing artificial price surges becomes unreliable as the increase of the total fare (profit per ride) may be compensated by the reduced number of customers, and a higher risk for drivers to miss out on potential customers when switching OFF their app. At low demand, larger δ intensifies the competition between drivers and forces them to stay ONline. The regime where remaining ONline is the Nash equilibrium extends to much higher surge fees than for inelastic demand. However, in the low demand limit $D < 1$ the coexistence of ON and OFF Nash equilibria persists for large possible surge fees as the risk becomes independent of the surge fee when there is less than one customer $D < 1$. Importantly, the regime where OFF is the pure strategy Nash equilibrium shrinks compared to the situation for $\delta = 0$.
- $\delta = 0.3$: For strongly elastic demand drivers are interacting in a highly competitive environment. For almost all $D < 2$ and very large surge fees, the Nash equilibrium is in ON-strategies as strong demand response which lead to expected profit loss for drivers that go OFFline (green in Supplementary Fig. 17c). The coexistence regime of ON and OFF Nash equilibria for low demand disappears for high elasticity $\delta = 0.3$. Only for low values $p_{\mathrm{surge}}^{\mathrm{max}} < 1.5$ and sufficiently strong demand $D \geq 2$, playing OFFline strategies remains a Nash equilibrium (orange in Supplementary Fig. 17c). In the intermediate regime, the incentive structure forces drivers into a mixed strategy equilibrium (blue-red striped in Supplementary Fig. 17c).

Hence, there exist four well pronounced regimes of Nash equilibria in ride-hailing games. While strong competition compels drivers to always remain ONline in the limit of low overall demand, the reverse applies to situations with high demand where going OFFline is the dominant strategy. Demand elasticities and the maximum possible surge fee modulate this picture. These results illustrate the basic incentives for drivers: high, inelastic demand and sufficient additional profit due to surge fees promotes drivers to exploit their control over the market and induce artificial price surges. High price elasticity (e.g. due to other competitive public transport options) increases competition and reduces these incentives.

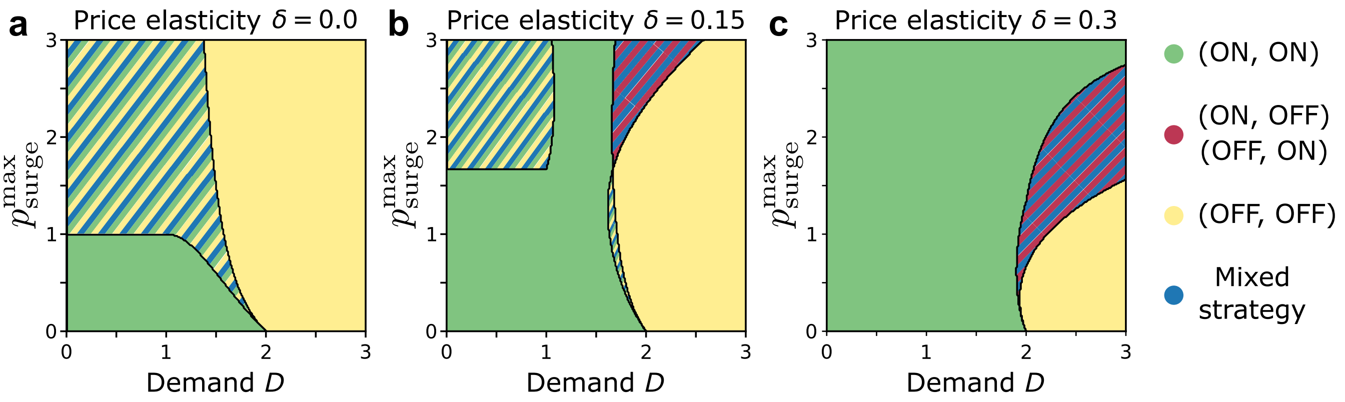

Supplementary Figure 17. **Low price elasticity and high demand promote artificial supply shortages.** **a**, Nash equilibria of the two-player game (see text) with completely inelastic demand, $\delta = 0$. As discussed above, at $D = 1$ the game transitions from a prisoner's dilemma at low $p_{\mathrm{surge}}^{\mathrm{max}} < 1$ with a single Nash equilibrium to a stag hunt with multiple Nash equilibria. For sufficiently high demand $D \geq 2$, the game becomes trivial with a single (OFF, OFF) socially optimal Nash equilibrium. **b**, Nash equilibria of the two-player game (see text) with elastic demand $\delta = 0.15$. **c**, Nash equilibria of the two-player game (see text) with elastic demand with higher elasticity $\delta = 0.30$. As the demand elasticity increases, ON becomes the only Nash equilibrium in a large part of the parameter space. Only for very high demand OFF remains a feasible strategy. Overall, these results illustrate that high demand and low price elasticity of demand incentivize drivers to create artificial supply shortages.

The above results illustrate that the incentives underlying artificial supply shortages emerge already in a fundamental setting with linear response functions. To demonstrate their robustness, we now consider a response function modeling a nonlinear demand response

$$D'(p') = D_0(1 - \delta^2(p' - p_{\text{base}})^2) \tag{S11}$$

such that the demand reacts more strongly to higher prices [see Supplementary Fig. 18, compare Eq. (S10)]. Weak demand response at low surge prices increases the incentives to create artificial supply shortages even for larger values of $\delta = (p_{\max} - p_{\text{base}})^{-1}$, encoding the maximum price until the demand is zero (same definition as above). The stronger demand response at higher prices naturally counteracts the driver's incentives to create strong price surges as the reduction of demand may cancel out the additional profit from higher fares. The phase diagrams illustrated in Supplementary Fig. 19 show qualitatively the same dynamics as for the linear response function (compare Supplementary Fig. 17).

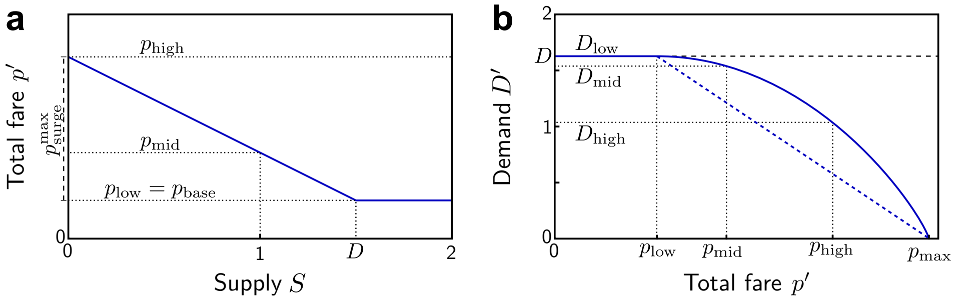

Supplementary Figure 18. **Quadratic demand response in the two-player game.** **a**, The dynamic pricing algorithm is the same as described above and reacts linearly as soon as the supply falls below the demand, increasing by up to $p_{\text{surge}}^{\max}$ without any supply. **b**, With a quadratic demand response [Eq. (S11)], the demand D' of customers reacts inelastic to small prices p' (e.g. small maximum surge fee $p_{\text{surge}}^{\max}$) but reacts stronger (steeper slope) at higher prices (e.g. large maximum surge fee $p_{\text{surge}}^{\max}$).

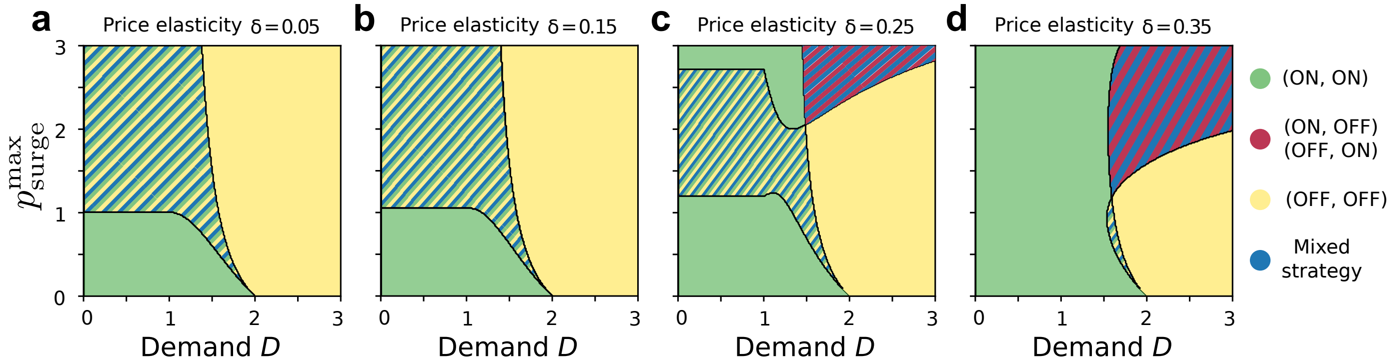

Supplementary Figure 19. **Phase diagram of Nash equilibria in the two player game with quadratic demand function.** Overall, the equilibrium states are qualitatively the same as with linear demand response. **a,b**, Nash equilibria of the two-player game (see text) with inelastic demand, $\delta = 0.05$ and $\delta = 0.15$, respectively. Due to the quadratic demand response (compare linear demand response in Supplementary Fig. 18), the demand is almost inelastic for the maximum surge fee shown in the phase diagram. The results are qualitatively identical to completely inelastic demand (Supplementary Fig. 17a). **c**, Nash equilibria of the two-player game (see text) with quadratic demand response and price elasticity $\delta = 0.25$. The transition phase between low and high price elasticity is different from the dynamics observed for linear response functions. In particular, the equilibrium states observed for low maximum surge fee $p_{\text{surge}}^{\max}$ still correspond to almost inelastic demand (zero derivative of the quadratic demand response at low prices), while the the states for high maximum surge fee $p_{\text{surge}}^{\max}$ correspond to high price elasticity (larger slope of the quadratic response at high prices). **d**, Nash equilibria of the two-player game (see text) with completely elastic demand with price elasticity $\delta = 0.35$. The dynamics are qualitatively similar to those observed in the linear model with highly elastic demand (Supplementary Fig. 17c) with the same dependence on the maximum surge fee $p_{\text{surge}}^{\max}$ explained above.

Multiplayer game with elastic demand

In a more realistic setting, a larger number $N > 2$ of drivers interact, making a decision to switch their ride-hailing application ON or OFF. In this section, we consider a version of the game described above with $N > 2$ players.

For simplicity, we consider only the case of high demand, where the total demand D is equal to the total supply N drivers. When drivers switch OFF their ride-hailing application, the dynamic pricing algorithm reacts to the apparent supply S and increases the price, as in the two-player game. We assume the same cost function Eq. (S9), that simplifies to

$$p'(\rho_{\mathrm{OFF}}) = p_{\mathrm{base}} + p_{\mathrm{surge}}^{\mathrm{max}}\, \rho_{\mathrm{OFF}} \tag{S12}$$

under the assumption of high demand, $D = N$. $\rho_{\mathrm{OFF}} = \frac{N-S}{N}$ is the fraction of drivers playing OFF and $p_{\mathrm{surge}}^{\mathrm{max}}$ is the maximal supply-driven surge fee when no drivers are available (e.g. when all drivers collectively switch to the OFF state). This dynamic pricing function implies that an individual driver turning his or her app OFF, $\rho_{\mathrm{OFF}} = 1/N$, changes the price by $p_{\mathrm{surge}}^{\mathrm{max}}/N$.

As before, customers react to the new price p'. With the above assumption, equation (S10) simplifies to

$$D'(p') = D_0 \left(1 - \frac{p' - p_{\mathrm{base}}}{p_{\mathrm{max}} - p_{\mathrm{base}}}\right) = D_0 \left(1 - \delta\, p_{\mathrm{surge}}^{\mathrm{max}}\, \rho_{\mathrm{OFF}}\right) \tag{S13}$$

where $\delta = (p_{\mathrm{max}} - p_{\mathrm{base}})^{-1}$ again describes the price elasticity of the demand.

Selfish action

We first consider this game in the case where all drivers act selfishly and try to maximize their own payoff. Since we assume a total demand $D = N$ and $p_{\mathrm{max}} \geq p_{\mathrm{base}} + p_{\mathrm{surge}}^{\mathrm{max}}$, a driver choosing to remain ONline will always be able to secure a ride. Their expected payoff is then

$$\Pi[\mathrm{ON}|\rho_{\mathrm{OFF}}] = p' = p_{\mathrm{base}} + p_{\mathrm{surge}}^{\mathrm{max}}\, \rho_{\mathrm{OFF}}. \tag{S14}$$

ON divers thus profit from the decision of other drivers to go OFFline. On the other hand, a driver playing the OFF strategy receives

$$\Pi[\mathrm{OFF}|\rho_{\mathrm{OFF}}] = \frac{D' - N\,(1-\rho_{\mathrm{OFF}})}{N\rho_{\mathrm{OFF}}}\, p' = (1 - \delta\, p_{\mathrm{surge}}^{\mathrm{max}})\, \left(p_{\mathrm{base}} + p_{\mathrm{surge}}^{\mathrm{max}}\, \rho_{\mathrm{OFF}}\right), \tag{S15}$$

where the first term corresponds to the probability of an OFF-driver to get one of the remaining $D' - N(1 - \rho_{\mathrm{OFF}})$ customers after ON drivers have already secured their rides (illustrated in Supplementary Fig. 20a).

Clearly, playing ON is always more beneficial for the individual player (see also Supplementary Fig. 20b). Still, a driver may choose to play OFF if the expected payoff is larger than the payoff $\Pi[\mathrm{ON}|\rho_{\mathrm{OFF}} = 0] = p_{\mathrm{base}}$ in the case where everyone remains ONline, that means when $(1 - \delta\, p_{\mathrm{surge}}^{\mathrm{max}})\left(p_{\mathrm{base}} + p_{\mathrm{surge}}^{\mathrm{max}}\, \rho_{\mathrm{OFF}}\right) \geq p_{\mathrm{base}}$. For large N, $\rho_{\mathrm{OFF}} = 1/N$ is small for a single driver. Therefore, the inequality is only true (selfish action beneficial) if N is sufficiently small, $N \leq N^* = \frac{1-\delta p_{\mathrm{surge}}^{\mathrm{max}}}{\delta p_{\mathrm{base}}}$.

Collective action

In contrast to selfish action, drivers may choose to cooperate to drive up the price and potentially increase their collective payoff $p'D'$ even if this is not beneficial for an individual driver (see Supplementary Fig. 20a). The expected collective payoff per driver is (see Supplementary Fig. 20b)

$$\begin{aligned}\frac{\Pi_{\mathrm{coll}}[\rho_{\mathrm{OFF}}]}{N} = \frac{p'\,D'}{N} &= \frac{\left(p_{\mathrm{base}} + p_{\mathrm{surge}}^{\mathrm{max}}\, \rho_{\mathrm{OFF}}\right)\, D_0\left(1 - \delta\, p_{\mathrm{surge}}^{\mathrm{max}}\, \rho_{\mathrm{OFF}}\right)}{N} \\ &= p_{\mathrm{base}} + p_{\mathrm{surge}}^{\mathrm{max}}\, \rho_{\mathrm{OFF}}\left(1 - \delta\, p_{\mathrm{base}} - \delta\, p_{\mathrm{surge}}^{\mathrm{max}}\, \rho_{\mathrm{OFF}}\right).\end{aligned} \tag{S16}$$

In the limit $\rho_{\mathrm{OFF}} \to 0$, this reduces to the average payoff $\frac{\Pi_{\mathrm{coll}}[\rho_{\mathrm{OFF}}=0]}{N} = \frac{D_0\, p_{\mathrm{base}}}{N} = p_{\mathrm{base}}$ when everyone remains ONline. Maximizing this collective payoff gives the socially optimal fraction of OFF players

$$\rho_{\mathrm{OFF}}^{*} = \frac{1 - p_{\mathrm{base}}\,\delta}{2\,\delta\, p_{\mathrm{surge}}^{\mathrm{max}}}, \tag{S17}$$

assuming $0 < \rho^*_{\mathrm{OFF}} < 1$, otherwise ρ^*_{OFF} takes the limiting value 0 or 1. In this socially optimal configuration, drivers only serve a fraction $1 - \frac{1-p_{\mathrm{base}}\delta}{2}$ of potential customers (illustrated in Supplementary Fig. 20) while all other customers choose a different mode of transport or wait for the price to drop.

Overall, cooperation between drivers allows them to achieve a higher collective payoff. In a repeated game, different drivers may rotate playing OFF for the benefit of the group, resulting in a game of cooperation (coordinated OFF) and defection (always ON) among the drivers. Note that the collective payoff depends only on the product $p^{\mathrm{max}}_{\mathrm{surge}}\,\rho_{\mathrm{OFF}}$. The qualitative behavior is thus independent of $p^{\mathrm{max}}_{\mathrm{surge}}$ and a general consequence of the dynamic pricing mechanism in this simplified model. If $p^{\mathrm{max}}_{\mathrm{surge}}$ is changed, the fraction of offline drivers ρ_{OFF} will change to compensate (as far as possible) and the overall price p', payoff and fraction of riders served remains unchanged. As for the two-player game, lower demand $D_0 < N$ or higher price elasticity of the demand increases the payoff gap between ON and OFF players, increasing the incentive to defect and making it more difficult to organize cooperation.

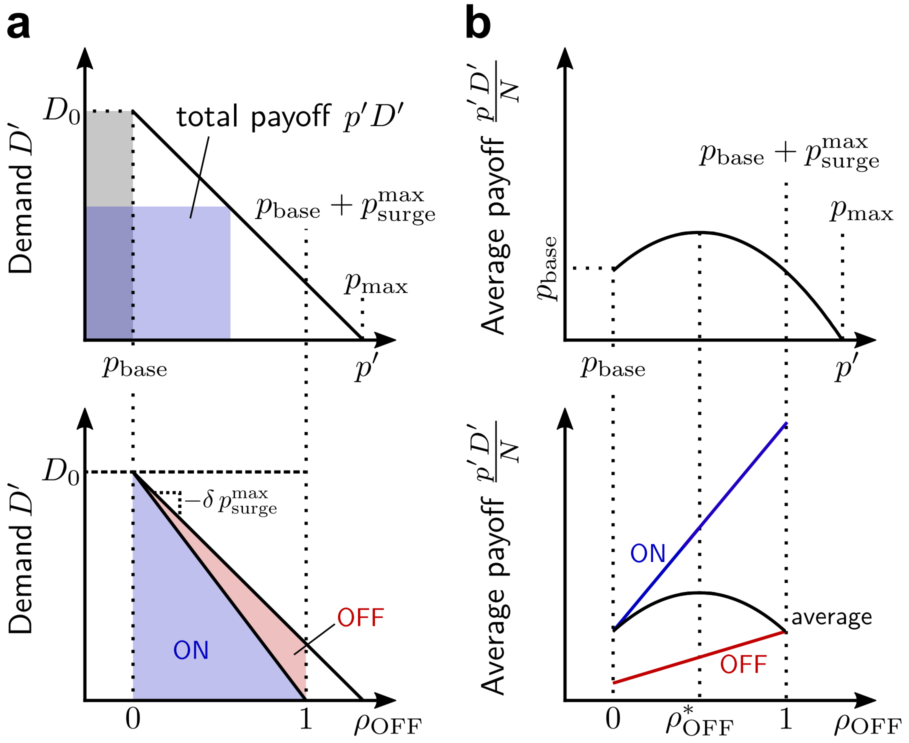

Supplementary Figure 20. **Price elasticity of demand and optimal payoff with collective action.** **a** The demand decreases as the price increases with price elasticity δ [top panel, compare Eq. (S13)]. The increase in price is linearly related to the fraction ρ_{OFF} of OFF drivers [Eq. (S12)] such that the demand also decreases linearly with ρ_{OFF} (bottom panel). For $p_{\mathrm{max}} > p_{\mathrm{base}} + p^{\mathrm{max}}_{\mathrm{surge}}$, the demand decreases such that every ON driver always gets a customer (bottom panel, shaded blue) and OFF drivers have to compete for the left-over customers (shaded red). **b** The average payoff per driver has a maximum at an intermediate price (top panel). This price corresponds to the optimal fraction ρ^*_{OFF} of OFF drivers Eq. (S17). As a group, all drivers achieve a maximal collective (socially optimal) payoff. However, for each driver individually, ON is the better strategy (bottom panel).

Dynamic multiplayer game under elastic demand

Based on the previous two games, we now consider a repeated dynamic game, where drivers interact with the demand not only once. Instead they do so in a time-continuous system, making decisions whether to switch their app ON or OFF all the time and being busy for some time when they serve a customer.

We consider a system where N drivers serve customers that arrive following a stochastic process. For simplicity, we model the arrivals of customers as a Poisson process with a constant rate λ. In particular, we assume a uniform request rate over time (a valid approximation during most of the day as suggested by our demand analysis in section Supplementary Note 1 and do not model, for example, the discrete arrivals of individual planes. This describes the limiting case where the time passengers need to cross the airport, claim baggage etc. is sufficiently heterogeneous to smooth out the discrete arrival events.

When a customer arrives, they check the current price of transportation and, depending on their willingness to pay, either book a ride, wait for some time or leave the system (e.g. choosing a different mode of transportation or a different ride-hailing service provider). As above, the maximum price a customer is willing to pay is distributed uniformly in the interval $[p_{\text{base}}, p_{\max}]$. When the customer requests a ride, the oldest (longest waiting) ONline driver at the airport is selected to serve the ride. Here, we include the time the driver may have spent OFFline in this waiting time. The driver then serves the customer and returns to the airport after a round-trip time t_s uniformly distributed in $[2t_d - \Delta, 2t_d + \Delta]$.

Drivers waiting idly at the airport may decide to switch OFF their app to induce a surge. We calculate the current price $p'(t)$ at time t based on the current number $N_{\text{idle}}(t)$ of ONline drivers at the airport similar to the prevoulsy discussed games as

$$p'(t) = \begin{cases} p_{\text{base}} & \mid \quad N_{\text{idle}}(t) \geq N_{\text{thresh}} \\ p_{\text{base}} + p_{\text{surge}}^{\max}\left(1 - \frac{N_{\text{idle}}(t)}{N_{\text{thresh}}}\right) & \mid \quad N_{\text{idle}}(t) < N_{\text{thresh}}\,. \end{cases} \tag{S18}$$

Since there is not absolute demand in this case, we encode the demand in the number of drivers N_{thresh} below which the surge fee begins. We take $N_{\text{thresh}} = \lambda\,(2t_d)$ as the number of drivers required to serve all $\lambda\,(2t_d)$ expected incoming requests before one of these drivers returns to the airport.

Note that we assume instantaneous updates of the price as a function of the current state of the system. There is no delay or dependence on the history of the system. As such, individual drivers can never profit themselves from the surge they induce (in contrast to the static games). When they go ONline, the price immediately decreases. Instead, other drivers may get higher payoffs and the drivers may profit as a group (as in the multiplayer game).

In the following we describe the parameters used in the model, give a mean-field calculation of the socially optimal strategy and describe the detailed dynamics of the time-continuous simulation here and in the main manuscript. Finally, we illustrate the robustness of the induced price surges across a range of model parameters.

Parameters

Parameters used to illustrate the dynamic model here and in the main manuscript are loosely based on the observed price estimates from Washington D.C. (DCA). For the base cost and possible surge fee, we take $p_{\text{base}} = 16$ and $p_{\text{surge}}^{\max} = 20$. We take $p_{\max} = 54$ as the maximum observed total fare for an Uber Black ride.

Other parameters were chosen to (i) be in line with realistic values and (ii) be in the correct range to exhibit price surges in the simulations. In particular, we choose the round-trip time $\langle t_s \rangle = 2t_d = 30\,\text{min}$, corresponding to the expected time of taxi rides from the airport of approximately $15\,\text{min}$, distributed in the interval $[2t_d - \Delta, 2t_d + \Delta]$ with $\Delta = 5$ minutes. We choose the request rate $\lambda = 2\,\text{min}^{-1}$, approximately in line with the average number of taxi trips recorded at DCA. Together with the number of drivers $N = 160$, these parameters corresponds to drivers spending $50\,\text{min}$ between each of their $30\,\text{min}$ rides waiting at the airport and to an average number of 100 drivers waiting at the airport at any given time.

With the above parameters we require at least $N_{\text{thresh}} = \lambda\,(2t_d) = 60$ drivers at the origin to avoid surge fees. When all drivers are always ONline, we have on average 100 drivers idle at the airport. In this setting, we thus expect a constant price p_{base} with only very rare fluctuations.

All simulations start with all drivers ONline and currently waiting for a request.

Mean field

In order to determine the optimal strategy for the drivers, we first consider a mean field description of the system described above. For this calculation, we are only interested in the average steady state values of the price p', the request rate λ' and the number of ONline, OFFline and idle drivers N_{ON} , N_{OFF} and N_{idle}.

On the demand side, of all potential customers only a fraction $\frac{p_{\max}-p'}{p_{\max}-p_{\mathrm{base}}}$ will actually request transport after checking the price. This effectively reduces the request rate to

$$\lambda' = \frac{p_{\max} - p'}{p_{\max} - p_{\mathrm{base}}}\lambda\,. \tag{S19}$$

Note that, as before, we assume that the demand relevant for calculating the surge fee does not change, i.e. there is no demand dependence in the price function Eq. (S18). The total rate λ of customers checking the price (though not necessarily requesting a ride) and N_{thresh} remain constant.

On the supply side, we are interested in the number N_{idle} of idle drivers at the airport to calculate the total fare. As in the static game, we assume a constant fraction ρ_{OFF} of drivers to be OFFline. On average, the remaining $N_{\mathrm{ON}} = N - N_{\mathrm{OFF}} = N\,(1-\rho_{\mathrm{OFF}})$ drivers spend $\langle t_s\rangle = 2t_d$ driving and $\langle t_w\rangle = N_{\mathrm{idle}}/\lambda'$ waiting at the airport for their next customer. Weighting the distribution of drivers with these times gives the number of drivers waiting idly as the airport as the fraction

$$N_{\mathrm{idle}} = \frac{\langle t_w\rangle}{\langle t_s\rangle + \langle t_w\rangle}\,N_{\mathrm{ON}}$$

which results in

$$\begin{aligned} N_{\mathrm{idle}} &= N_{\mathrm{ON}} - \lambda'\,(2\,t_d) \\ &= N_{\mathrm{ON}} - 2\,\lambda\,t_d\,\frac{p_{\max} - p'}{p_{\max} - p_{\mathrm{base}}} \end{aligned} \tag{S20}$$

Together with the dynamic price function Eq. (S18), we now have a self-consitency condition for relating the price p' and the number of idle drivers N_{idle} [Eq. (S18) and (S20)]. The solution of this self-consisstency condition gives the equilibrium total fare p' and the optimal number of ONline drivers N_{ON}. Due to the number of case distinctions arising from to the piece-wise definition of the price and to ensure, for example, $0 \le N_{\mathrm{idle}} \le N_{\mathrm{ON}}$ for all possible parameter choices, we do not give the full solution here. Instead, the solution is illustrated in Supplementary Fig. 21.

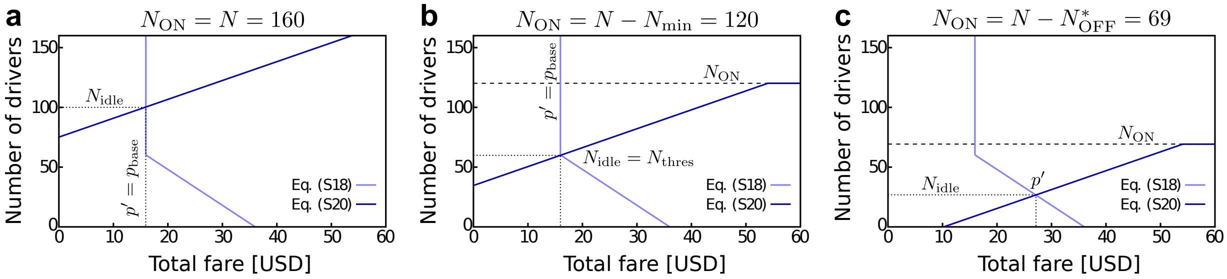

Supplementary Figure 21. **Self-consistency condition for the price and the number of idle drivers.** The plots show the functions $p'(N_{\mathrm{idle}})$ [Eq. (S18), light blue] and $N_{\mathrm{idle}}(p')$ [Eq. (S20), dark blue] when **a** all drivers are ONline, **b** $N_{\min} = 40$ drivers are OFFline at the boundary to price surges and **c** in the mean-field optimal case when $N^{*}_{\mathrm{OFF}} = 91$ drivers are OFFline. The price increases only when sufficiently many drivers $N_{\mathrm{OFF}} > N_{\min}$ go OFFline.

With the price and the average request rate in equilibrium, the average earning rate of a single ONline driver follows as the profit from a ride divided by the total time for one ride (round-trip time and waiting for the next ride) as

$$\Pi_{\mathrm{ON}} = \frac{p'}{\langle t_s\rangle + \langle t_w\rangle} = \frac{p'\lambda'}{2t_d\,\lambda' + N_{\mathrm{idle}}} \tag{S21}$$

and the collective earning rate as the weighted average with the OFFline drivers, who earning nothing,

$$\Pi_{\mathrm{coll}} = (1-\rho_{\mathrm{OFF}})\,\Pi_{\mathrm{ON}} = \frac{p'\lambda'\,(1-\rho_{\mathrm{OFF}})}{2t_d\,\lambda' + N_{\mathrm{idle}}}\,. \tag{S22}$$

For the parameters described above ($\langle 2\,t_d \rangle = 30\,\text{min}$, $N = 160$), we obtain the following results: If all drivers are always ONline, they earn an average of 12 USD per hour or $p = p_{\text{base}} = 16$ USD per ride with $30 + 50$ minutes between rides. The drivers can earn more if they reduce the number of idle ONline drivers to at most $N_{\text{thresh}} = 60$ drivers by switching OFF their app. They maximize their profit when only $N^*_{\text{idle}} = 26$ drivers are available at the airport ($N^*_{\text{ON}} = 69$), collectively earning 14.39 USD per hour. Here, the ON-drivers earn $p^* = 27.14$ USD per ride with $30+18.8$ minutes between rides but approximately 57% of all drivers are OFFline and earn nothing.

Simulation

In our simulation of the system, we make a few additional assumptions for the strategy of the drivers and the behavior of the customers. In particular, we assume that the drivers use a strategy informed by the above mean field calculations: Drivers switch OFF their app if there are enough drivers willing to participate in a surge to reduce the number of idle drivers to at least N_{thresh}. However, drivers switch OFF their app only while there are more than N^*_{idle} idle drivers at the airport (i.e. while the price is lower than their optimal price), otherwise they wait to serve requests and collect the higher total fare.

While the drivers collectively earn the most with this optimal strategy, a single driver who stays OFFline for the benefit of the group would earn very little or even nothing. To ensure an equitable distribution of payoff across drivers, we assume that the drivers are not perfectly social and are only willing to remain OFFline for a limited amount of time. Drivers turn their app back ON after at most $t^{\max}_{\text{OFF}} = 20\,\text{min}$ and only participate in one surge per ride. This means, if a driver participated in a surge, they will not participate in another surge until after they served a ride and received some payoff. This ensures that each driver earns a similar amount over the course of the simulation. In addition, such a behavior might more realistically reflect actions of drivers who cannot switch their app ON and OFF constantly, for example to avoid being detected by automated algorithms of the service provider.

On the customer side, we assume that customers do not immediately leave the system but instead wait for $t^{\text{cust}}_{\text{wait}} = 10\,\text{min}$, checking the price every $\Delta t^{\text{cust}} = 2\,\text{min}$ before finally deciding to leave the system. Note that the maximum price they are willing to pay does not change during this time.

We aggregate the earnings of all drivers over $T_{\text{sim}} = 7\,\text{days}$ of continuous requests (roughly equivalent to a month real time with 6 hours of requests per day). For perfectly social drivers that are willing to remain OFFline indefinitely (but still only participate in one surge per ride) the simulation accurately reproduces the predicted outcome of the mean field calculations, illustrated in Supplementary Fig. 22. The drivers keep the price constant and close to its optimal value. They collectively earn 14.43 USD per hour on average (standard deviation across drivers 1.12 USD per hour and minimal earning of an individual driver of 11.23 USD per hour) in the simulation, agreeing with the predicted 14.39 USD per hour from mean field calculations. Due to the fluctuations in the demand and the total number of drivers at the airport, the number of drivers required to be OFFline also fluctuates. This creates a sufficient mixing of OFFline and ONline drivers over the course of the simulation to distribute the payoff relatively equally.

For only partially social drivers, the dynamics changes (Supplementary Fig. 23). Drivers switch OFF their app but return ONline after $t^{\max}_{\text{off}} = 20$ minutes, ending the surge. After the surge, there are not sufficiently many drivers willing to participate in another surge and the price relaxes to its base value p_{base} as all drivers are ONline. Only after these drivers have served a customer and returned to the airport, a new surge starts when sufficiently many drivers are willing to participate again. These dynamics determine both the timescale for the duration of the surges (explicitly as $t^{\max}_{\text{off}} = 20$) and the time between the surges (implicitly via the driver turn-over rate depending on the request rate λ). The induced surge peaks typically reach the driver-optimal price for a short time, resulting in a characteristic pattern of repeated short price spikes with (almost) fixed amplitude. In reality, the dynamics would be affected also by the timescale of the dynamic pricing algorithm reacting to the supply and demand changes as well as by additional demand-side incentives such as fewer alternative transport options (resulting in lower price elasticity of customers or higher willingness to pay) late at night and other external influences such as traffic conditions and variable round-trip time (see Supplementary Fig. 26 or Fig. 2c in the main manuscript). Overall, the drivers earn 13.64 USD per hour with this strategy (standard deviation 0.24 USD per hour and minimal earning 12.98 USD per hour), corresponding to lower total earnings but also much lower risk for extremely low income.

In contrast, when drivers remain always ONline, they only earn 12.07 USD per hour on average (standard deviation 0.04 USD per hour), agreeing with the 12 USD per hour expected from the mean field calculation (see above). Thus, drivers collectively increase their profits compared to the baseline always-ON strategy but they do not achieve the optimal payoff. However, in contrast to the case of perfectly social drivers who remain OFFline indefinitely, they keep

the distribution of income much more similar among all drivers without the need to explicitly share the profits at the end of the game, reducing the risk for individual drivers to participate. Overall, the optimal strategy for the drivers depends on their risk-aversion preferences and their trust in the other drivers to cooperate (during the current day as well as across longer timescales).

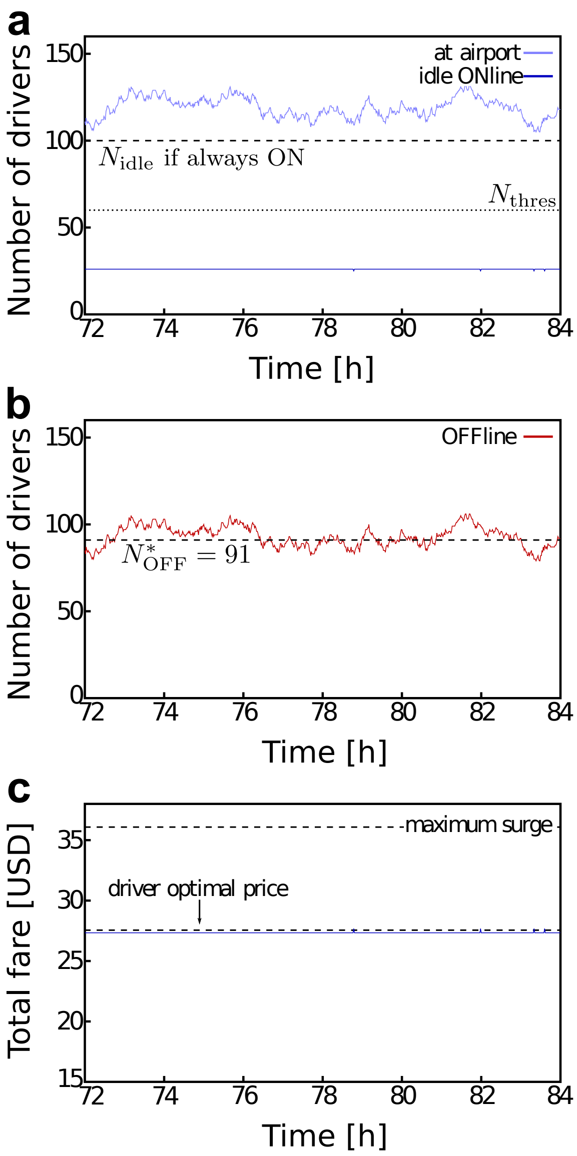

Supplementary Figure 22. **Perfectly social drivers reproduce mean-field estimates. a** Total number of drivers at the airport (light blue) and number of idle ONline drivers (dark blue) at the airport. **b** Number of OFFline drivers. Perfectly social drivers are willing to remain OFFline indefinitely, resulting in the constant (mean-field optimal) number of ONline drivers $N_{\mathrm{ON}} = N^*_{\mathrm{ON}} = 26$ in panel **a**. **c** The resulting price is constant close to the mean-field optimal price. However, only ONline drivers actually earn this payoff for rides.

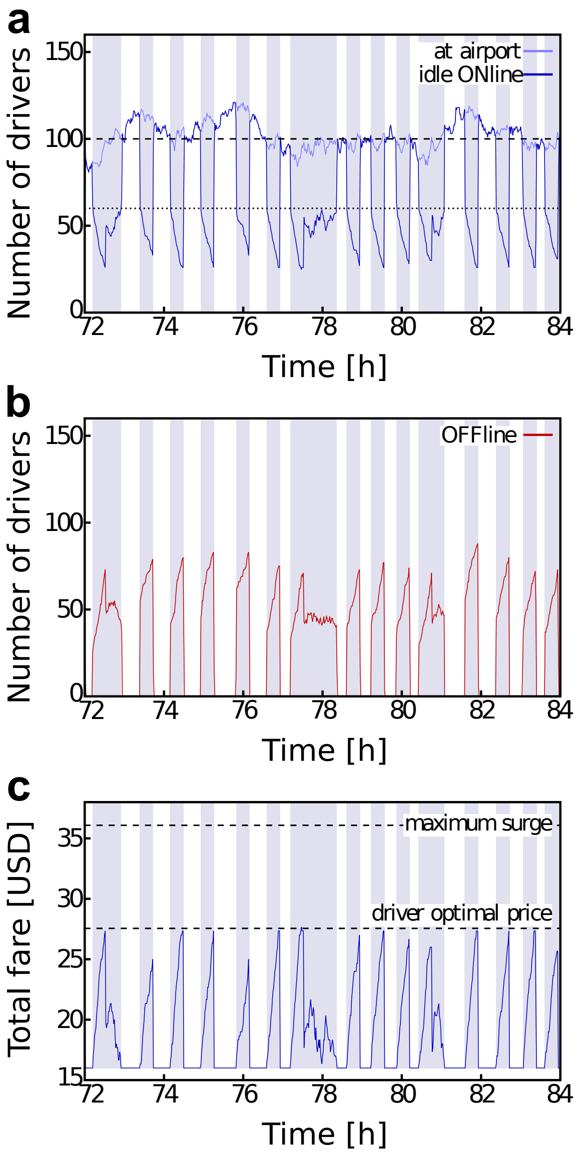

Supplementary Figure 23. **Partially social drivers induce repeated price surges. a** Total number of drivers at the airport (light) and number of idle ONline drivers (dark) at the airport. **b** Number of OFFline drivers. Partially social drivers remain OFFline for at most $t^{\max}_{\mathrm{OFF}} = 20$ minutes and only participate in one surge per ride, resulting in repeated periods where a large number of drivers are offline. **c** The resulting price reflects the anomalous supply dynamics. The drivers' strategy results in repeated price surges with a characteristic amplitude and duration when sufficiently many drivers at the airport are willing to participate in a surge. As drivers rotate being OFFline, all drivers profit equally from these price surges.

Robustness

The qualitative results of the model are robust to changes of the parameters, such as the price limits and thresholds. In particular, the qualitative picture of repeated surge peaks occurs for a range of values of $t_{\mathrm{OFF}}^{\max}$. This parameter explicitly sets the timescale of surges, describing how social or trusting the drivers are (see Supplementary Fig. 24a-c). When $t_{\mathrm{OFF}}^{\max} > 2t_d$, drivers are sufficiently social to bridge the time until another driver has serviced a customer and is willing to join the surge again. This leads to a permanently high surge fee since some drivers are OFFline at all times. As $t_{\mathrm{OFF}}^{\max}$ increases, the dynamics become more and more similar to the mean-field limit where drivers remain OFFline indefinitely (compare Supplementary Fig. 24d-f).

The second relevant timescale in the system is the driver turn over rate, influencing the duration between individual surges. This turn over rate is directly related to the request rate λ (demand). As the demand decreases, more drivers need to go OFFline to induce a surge since fewer drivers are required to serve the predicted upcoming requests. That means the parameter N_{thresh} in the price function Eq. (S18) decreases while the total number of drivers remains constant, $N = 160$. This makes surges more difficult to coordinate and less frequent, as drivers participating in one surge need longer times to serve a ride and become willing to induce a surge again. Resulting time series for different request rates are illustrated in Supplementary Fig. 25. The results reflect the qualitative intuition from the two previous discussions: a lower demand makes it more difficult to organize surges as the incentive to defect is higher and more trust is required between the players.

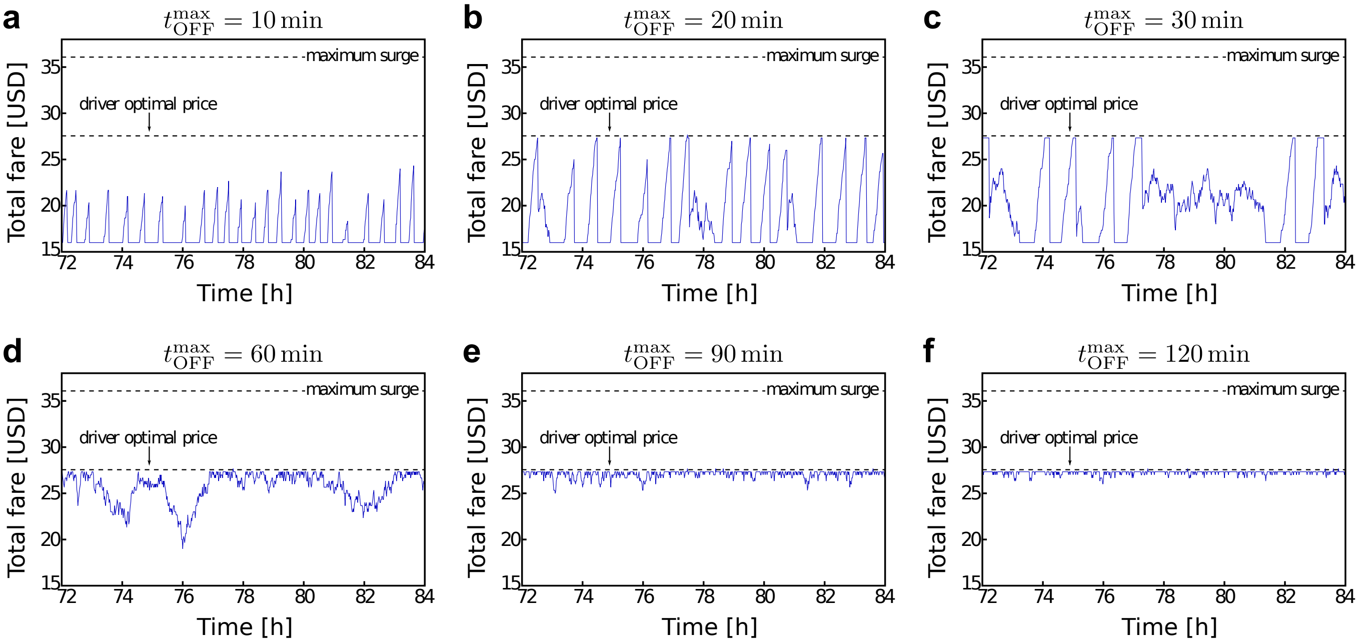

Supplementary Figure 24. **Repeated price surges transition into constant surge as drivers become more social. a-c** When drivers are only weakly social and want to ensure a high payoff for themselves, they remain OFFline only for a short amount of time $t_{\mathrm{OFF}}^{\max} \leq 2t_d = 30$ minutes. After a surge, most drivers are not willing to participate in another one. Only when sufficiently many drivers have served a ride they initiate the next surge. This leads to repeated short price spikes with characteristic amplitude and duration set by $t_{\mathrm{OFF}}^{\max}$. **d-f** When drivers are more trusting and social (sharing of profits, reciprocation over longer timescales) and $t_{\mathrm{OFF}}^{\max} > 2t_d$, drivers can bridge the time until a driver returns from a ride. This creates a permanently high surge fee, similar to the mean-field limit (compare Supplementary Fig. 22).

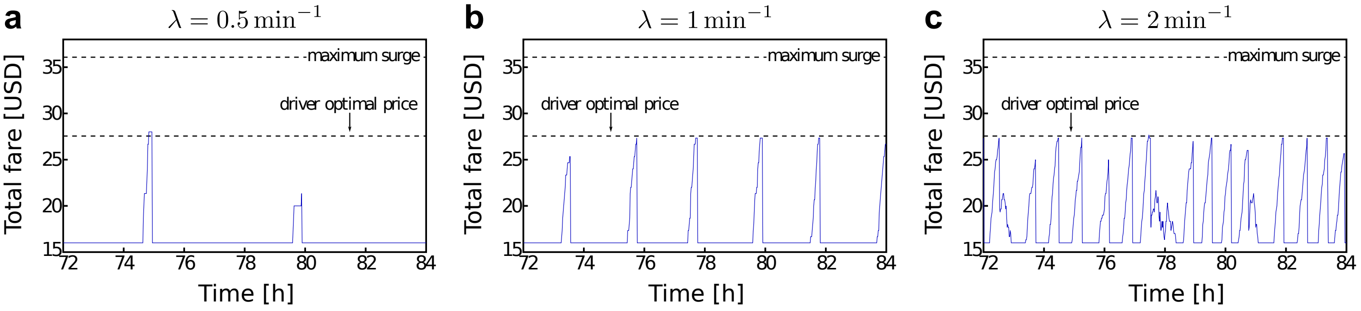

Supplementary Figure 25. **Price surges are more common with higher demand.** As the demand increases (request rate λ increases from **a** to **c**), surges become more frequent. With the request rate, the driver turn over rate increases. As a consequence, drivers are willing to participate in surges more often and the time between surges decreases.

Variable travel time

In the main manuscript, we showed simulation results with variable travel time (variable base cost) to more closely model the daily dynamics including rush hour and periods of heavy traffic. For these simulations, we modulate the round-trip time such that $\langle t_s \rangle$ increases to 60 minutes during rush hour (doubling the standard travel time from $t_d = 15$ to $t_d = 30$, approximately reflecting the average taxi trip duration during rush hour from DCA) to more closely replicate the price dynamics observed at DCA. As the round-trip time changes during the simulation, we adjust the driver strategy based on the optimal mean-field strategy assuming static conditions with the current round-trip time. We use the price function $p_{\text{base}} = 1\,\text{USD} + t_d\,\frac{\text{USD}}{\text{minute}}$ to determine the base cost (changing from 16 USD to 31 USD) depending on the expected travel time. At the same time, the threshold before surge pricing sets in also changes over time based on the current round-trip time, calculated as described above as the number of drivers required to bridge the time until the first driver returns, $N_{\text{thresh}} = \lambda\,(2t_d)$.

Supplementary Figure 26 shows the same results as Fig. 2c in the main manuscript with additional detail on the supply and demand in the last panel. The supply (number of drivers at the airport) was converted to units of trips per minute to be comparable to the demand by comparing it to the number of requests that can be served before surge sets in,

$$\text{supply} = 1 + \frac{\text{number of drivers at airport}}{\lambda\,(2t_d)} = 1 + \frac{\text{number of drivers at airport}}{N_{\text{thresh}}}\,, \tag{S23}$$

effectively assuming $\lambda\,(2t_d)$ drivers are currently busy. This means surge pricing sets in as soon as the supply is lower than the expected demand $\lambda = 2$ requests per minute. The expected equilibrium supply follows as

$$\text{equilibrium supply} = \frac{\text{total number of drivers}}{\lambda\,(2t_d)} = \frac{\text{total number of drivers}}{N_{\text{thresh}}}\,, \tag{S24}$$

Due to the stochasticity of the demand (shown as a 60 minute moving average to avaerage the comparatively low number of requests), the number of requests per minute is not constant but fluctuates around the expected value. Correspondingly, the available supply varies stochastically around the mean field equilibrium supply. Note that the sotchastic fluctuations of the demand or supply are not correlated with the induced price surges.

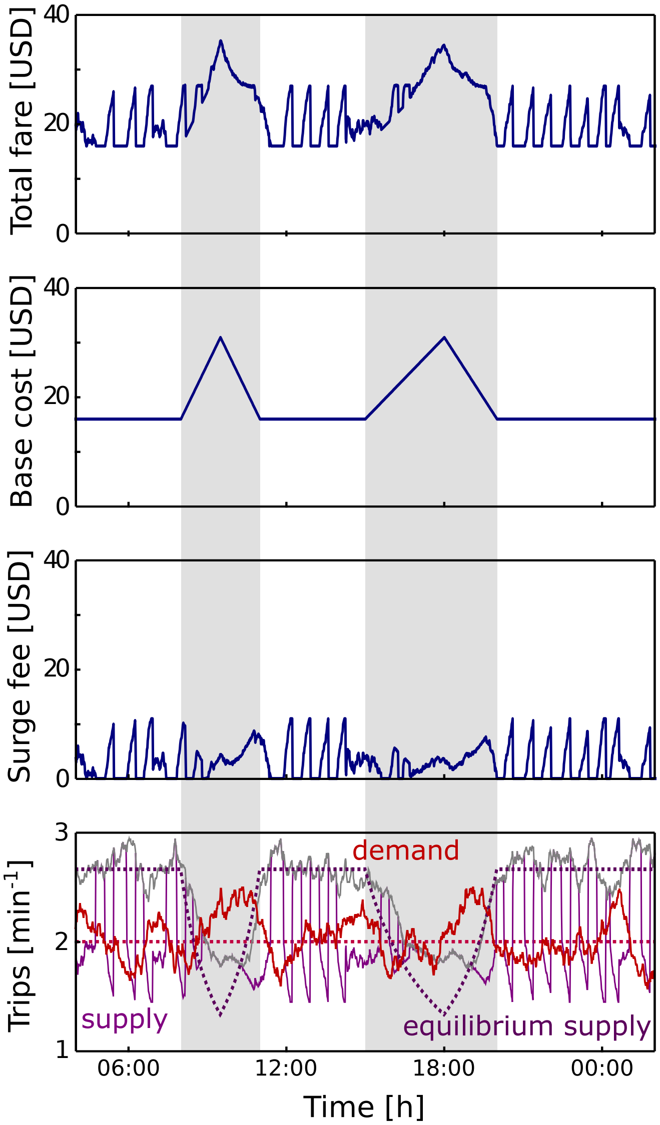

Supplementary Figure 26. **Simulation with variable travel time qualitatively reproduces surge dynamics observed at DCA.** The figure shows the total fare, base cost and surge fee of the simulation replicating typical rush hour dynamics with variable travel time during morning and evening commuting hours (compare Fig. 2c in the main manuscript). The last panel shows additional details on the supply and demand evolution. The supply (grey, number of drivers at the airport) fluctuates corresponding to the stochastic demand (60 minute moving average, solid red line). The actual supply of online drivers (solid purple line) shows repeated supply shortages creating price surges. The dashed lines indicate the average request rate (demand, red) and the mean field equilibrium number of drivers expected at the airport at the given conditions and full demand (ignoring reduced demand due to increased price).

Summary

The actual mechanism used by Uber to assign ride requests to drivers at airports is unknown. In particular not all drivers that take a trip from the airport will immediately return and not all drivers will have to wait a long time and be able or willing to coordinate. For example, a driver arriving at the airport terminal to drop off a passenger may be preferred to immediately pick up a customer due to lower waiting time since this driver is already at the terminal compared to drivers waiting in a parking lot further away. The exact dynamics depend also on the layout of the airport and local regulations (where drivers may park, how long customers take to exit the airport and book a ride etc.). Moreover, while we showed in the main manuscript and in Supplementary Note 1 of this document that the demand is approximately constant over the day and the price surges are not significantly correlated with the arrivals, fluctuations due to clustered arrivals of airplanes likely still enter in the pricing algorithm. Importantly, clustered arrivals and temporarily increased demand also change the incentive structure, as the artificially induced price surges become easier to organize and more profitable at higher demand as illustrated in all above models. Moreover, dynamic pricing algorithms typically do not react instantaneously to changes. A longer reaction timescale may allow drivers to profit from their own induced surge (as assumed in the discussion of the two-player and multiplayer static model). This may also contribute to easier organization of artificial price surges.

Overall, while the above models do not exactly replicate reality, they qualitatively illustrate the incentives inherent in dynamic pricing mechanisms for drivers to maximize their profit by collectively inducing price surges. In particular, they illustrate that these incentives exist even with the most basic form of dynamic pricing over a range of models, showing that artificial supply shortages are a general problem across dynamic pricing schemes. Specifically, the models illustrate the following:

- The two-player model illustrates that the basic incentive exists in the simplest possible setting independent of the parameters of the pricing algorithm and provides a simple model to understand the conditions that promote anomalous supply dynamics.

- The multiplayer game extends this setting and illustrates that the effect remains when drivers act in a socially optimal way. This one-time game would describe the dynamics when the demand is strongly localized in time, for example for simultaneous requests from many passengers arriving on the same plane.

- The dynamic model serves to illustrate that repeated price surges may emerge in a simple model, even without explicit planning. In particular, it illustrates that these price surges emerge for a broad range of parameters and that the incentives illustrated in the static, one-off games are sufficient to induce repeated price surges.

We note that the same arguments made here for supply-induced price surges in principle also hold for demand-induced surge fee manipulation, for example by temporarily reducing demand to lower the price. Naturally, organizing such collective action on the demand side is much harder (as all players are strangers) and the game is typically not repeated with the same group of people (more incentive to defect). Additionally, service providers have little incentive to reduce prices as the price for ride-hailing services is typically already low (due to competition with other service providers, taxi cabs and public transport), leaving not much room for price decreases. Thus, while the mechanism in principle translates to demand-induced down-surges, the conditions make these much harder to execute than supply-induced up-surges.

Supplementary Note 4. Data, methodology and details

Dynamic pricing mechanisms are increasingly adopted in on-demand mobility services. In ride-hailing, Uber Technologies Inc. operates one of the largest digital marketplaces where more than 90 million monthly active users from all around the world request rides and are matched with a driver subject to a dynamically adapted price [13]. In the main manuscript, we illustrated how specific market conditions incentivize collective action among drivers that induce non-stationary price dynamics. We based our findings on Uber price estimate time series collected for trip in different locations all around the globe. We compared the price data against time series of aircraft deplanements collected for Reagan National Airport (DCA) as well as historic taxi trip records in Washington D.C. In this section of the Supplementary Information, we provide detailed information on all sources of data, our data acquisition approach, data content and quality as well as how we analyzed the raw data to obtain our main findings.

Data sources and acquisition

In this subsection, we detail the different data sources used to acquire the data required to conduct our analyses.

Acquisition of Uber price time series

Uber price estimates

Uber's price-estimate API endpoint provides, amongst others, price estimates for the company's different ride-hailing services on a per trip basis. For a given trip characterized by origin-destination coordinates (o, d), the interface returns a current price estimate vector, whose entries $i = \{1, 2, \dots\}$ contain a lower and upper bound p^i_{min} and p^i_{max} per Uber product i operated in the area, a trip duration estimate t_d, as well as a trip distance estimate l. Note that trip duration and distance estimates are universal for the different Uber products. We attach a time stamp t per tuple $((o, d), \mathbf{p}_{\text{min}}, \mathbf{p}_{\text{max}}, l, t_d)_t$ and define the ride-hailing price dynamics as the time series $\{((o, d), \mathbf{p}_{\text{min}}, l, t_d)_t, t \in T\}$ where T is the set of all measurement times. In our price estimate time series analysis, we use the lower bound on the price estimate $\mathbf{p}_{\text{min}}$ as the reference quantity for our calculations.

Uber products

We focus on Uber's standard ride-hailing services. Depending on local availability, we use one the two economy products UberX or UberGO (India). For comparison we also consider one of the six premium products Black, Berline, Exec, Lux, Premier or Select depending on the availability at the location.

Uber's products API endpoint provides, amongst others, information on fee structure parameters for each of these products. We extract the information on booking fees, price per minute, price per mile, distance unit, minimum fees and the currency code (see first section of this document for details on Uber's price model and Supplementary Tab. I for an example of the data) for the standard and premium Uber service operating in the respective area. Together with the price estimate time series we use these parameters to compute the base cost and isolate the surge dynamics not explained by distance and duration of the ride (compare Figs. 2-3).

City and trip characteristics

We compare the price dynamics for Uber standard and premium rides originating from different points of interests in metropolitan areas around the globe. For four types of trip categories we record and analyze Uber price estimate time series. These categories are:

- Airport: trip originates from an airport and ends at a point of interest in the nearby city center (e.g. train station, business district, convention center)
- Station: trip originates from a major train station and ends at a point of interest in the corresponding city (e.g. other train station, business district, convention center)
- Convention: trip originates from a local convention center and ends at a point of interest in the corresponding city (e.g. train station, business district)
- City: trip originates and ends within the boundaries of a specific city, not specifying a distinct point of interest (e.g. residential area, business district)

Note that the choice of trip origin and the local demand and supply situation (at the origin) are the primary determinants for Uber's dynamic pricing mechanism, while the precise choice of destination within a city does not significantly affect the results. Hence, the chosen trip categories classify the ride-hailing price dynamics at points of interest around the globe where demand for rides is typically highly localized and expected to change periodically during a day.

Supplementary Tab. II shows a full list of all 137 trips that Uber price estimate time series have been recorded for, defined in terms of city, region, as well as specifics on origin-destination attributes. It contains 63 airport (see Supplementary Fig. 1), 12 station, 23 convention and 39 city trip items. Per item, we provide detailed information on origin-destination coordinates, API request rates, time intervals of data recording, standard and premium Uber products used for the analysis, as well as aggregated measures of the surge dynamics in the spreadsheet DataRideHailingPrices.xlsx available in the data section of the online Supplementary Information.

We selected the trip sample based on three criteria (in order of priority):

- Uber activity: Uber operates in the local area.
- Global representation: Balanced mix of regions across the globe.
- Mixed points of interests: Balanced mix of airport, station, convention and city trips to investigate effects of the qualitatively different demand structure expected at these locations.

Supplementary Table II: **Uber price estimates have been recorded for airport, station, city and convention trips around the world.** The table provides the location (city and region) as well as trip classification characteristics (origin and destination types). Labels are included for reference to the figures in the first two sections of this document. Further trip details can be found in the spreadsheet DataRideHailingPrices.xlsx available in the online Supplementary Information.

**Label**	**City**	**Region**	**Origin type**	**Destination type**
ARN	Stockholm	Europe	Airport	Station
ATL	Atlanta	North America	Airport	City
BMA	Stockholm	Europe	Airport	Station
BOG	Bogota	South America	Airport	City
BOM	Mumbai	Asia	Airport	Station
BRU	Brussels	Europe	Airport	Station
BWI	Baltimore	North America	Airport	Station
BWI Residential Area Trip	Baltimore	North America	Airport	City
BWI Urban Area Trip	Baltimore	North America	Airport	City
CAI	Cairo	Africa	Airport	Station
CDG	Paris	Europe	Airport	Station
CPT	Cape Town	Africa	Airport	City
DCA	Washington D.C.	North America	Airport	Station
DCA Residential Area Trip	Washington D.C.	North America	Airport	City
DCA Urban Area Trip	Washington D.C.	North America	Airport	City
DEL	Dheli	Asia	Airport	Station
EWR	New York City	North America	Airport	Station
EWR Residential Area Trip	New York City	North America	Airport	City
EWR Urban Area Trip	New York City	North America	Airport	City
EZE	Buenos Aires	South America	Airport	City
GIG	Rio de Janeiro	South America	Airport	Station
GLA	Glasgow	Europe	Airport	Station
GVA	Geneva	Europe	Airport	Station
HEL	Helsinki	Europe	Airport	Station
HKG	Hong Kong	Asia	Airport	Station
IAD Residential Area Trip	Washington D.C.	North America	Airport	City
IAD Urban Area Trip	Washington D.C.	North America	Airport	City
IAH	Houston	North America	Airport	City
ICN	Seoul	Asia	Airport	Station
JFK	New York City	North America	Airport	Station
JFK Residential Area Trip	New York City	North America	Airport	City
JFK Urban Area Trip	New York City	North America	Airport	City
JNB	Johannesburg	Africa	Airport	City
LAX	Los Angeles	North America	Airport	Station

Continued on next page...

Supplementary Table II – continued from previous page

Label	City	Region	Origin type	Destination type
LGA	New York City	North America	Airport	Station
LGA Residential Area Trip	New York City	North America	Airport	City
LGA Urban Area Trip	New York City	North America	Airport	City
LHR	London	Europe	Airport	City
LIS	Lisbon	Europe	Airport	Station
MAA	Chennai	Asia	Airport	City
MAN	Manchester	Europe	Airport	Station
MEL	Melbourne	Oceania	Airport	City
MIA	Miami	North America	Airport	Station
MRS	Marseille	Europe	Airport	Station
MSY	New Orleans	North America	Airport	Station
MUC	Munich	Europe	Airport	Station
OAK Residential Area Trip	Oakland	North America	Airport	City
OAK Urban Area Trip	Oakland	North America	Airport	City
ORD	Chicago	North America	Airport	Station
OTP	Bucharest	Europe	Airport	Station
PER	Perth	Oceania	Airport	City
PHL	Philadelphia	North America	Airport	City
SEA	Seattle	North America	Airport	Station
SFO	San Francisco	North America	Airport	Station
SFO Residential Area Trip	San Francisco	North America	Airport	City
SFO Urban Area Trip	San Francisco	North America	Airport	City
SJC Residential Area Trip	San Francisco	North America	Airport	City
SJC Urban Area Trip	San Francisco	North America	Airport	City
TLL	Tallinn	Europe	Airport	Station
VNO	Vilnius	Europe	Airport	Station
WAW	Warsaw	Europe	Airport	Station
YUL	Montreal	North America	Airport	Station
YYZ	Toronto	North America	Airport	Station
Hoboken	Hoboken	North America	City	City
Meatpacking District (NYC)	New York City	North America	City	City
New York City Trip 1	New York City	North America	City	City
New York City Trip 2	New York City	North America	City	City
New York City Trip 3	New York City	North America	City	City
New York City Trip 4	New York City	North America	City	City
New York City Trip 5	New York City	North America	City	City
New York City Trip 6	New York City	North America	City	City
New York City Trip 7	New York City	North America	City	City
New York City Trip 8	New York City	North America	City	City
New York City Trip 9	New York City	North America	City	City
New York City Trip 10	New York City	North America	City	City
New York City Trip 11	New York City	North America	City	City
New York City Trip 12	New York City	North America	City	City
New York City Trip 13	New York City	North America	City	City
New York City Trip 14	New York City	North America	City	City
New York City Trip 15	New York City	North America	City	City
New York City Trip 16	New York City	North America	City	City
New York City Trip 17	New York City	North America	City	City
New York City Trip 18	New York City	North America	City	City
New York City Trip 19	New York City	North America	City	City
San Francisco City Trip 1	San Francisco	North America	City	City
San Francisco City Trip 2	San Francisco	North America	City	City
San Francisco City Trip 3	San Francisco	North America	City	City
San Francisco City Trip 4	San Francisco	North America	City	City
San Francisco City Trip 5	San Francisco	North America	City	City
San Francisco City Trip 6	San Francisco	North America	City	City
San Francisco City Trip 7	San Francisco	North America	City	City
San Francisco City Trip 8	San Francisco	North America	City	City
San Francisco City Trip 9	San Francisco	North America	City	City
Trump Tower (NYC)	New York City	North America	City	City
Washington, D.C. City Trip 1	Washington D.C.	North America	City	City

Continued on next page...

Supplementary Table II – continued from previous page

Label	City	Region	Origin type	Destination type
Washington, D.C. City Trip 2	Washington D.C.	North America	City	City
Washington, D.C. City Trip 3	Washington D.C.	North America	City	City
Washington, D.C. City Trip 4	Washington D.C.	North America	City	City
Washington, D.C. City Trip 6	Washington D.C.	North America	City	City
Washington, D.C. City Trip 7	Washington D.C.	North America	City	City
Washington, D.C. City Trip 8	Washington D.C.	North America	City	City
Washington, D.C. City Trip 9	Washington D.C.	North America	City	City
Americas Center Convention Complex	St. Louis	North America	Convention	Airport
Austin Convention Center	Austin	North America	Convention	Airport
Boston Hynes Convention Center	Boston	North America	Convention	Airport
Colorado Convention Center	Denver	North America	Convention	Airport
Georgia World Congress Center	Atlanta	North America	Convention	Airport
Henry B. Gonzalez Convention Center	San Antonio	North America	Convention	Airport
Houston Convention Center	Houston	North America	Convention	Airport
Hutchinson Convention Center	Dallas	North America	Convention	Airport
Jacob K. Javits Convention Center	New York City	North America	Convention	Airport
Los Angeles Convention Center	Los Angeles	North America	Convention	Airport
Minneapolis Convention Center	Minneapolis	North America	Convention	Airport
Moscone Center	San Francisco	North America	Convention	Airport
Nashville Music City Center	Nashville	North America	Convention	Airport
New Orleans Morial Center	New Orleans	North America	Convention	Airport
Orange County Convention Center	Orange County	North America	Convention	Station
Pennsylvania Convention Center	Philadelphia	North America	Convention	Airport
Phoenix Convention Center	Phoenix	North America	Convention	Airport
Riverside Convention Center	Riverside	North America	Convention	Airport
San Diego Convention Center	San Diego	North America	Convention	Airport
Seattle Convention Center	Seattle	North America	Convention	Airport
Tampa Convention Center	Tampa	North America	Convention	Airport
Walter E. Washington Convention Center	Washington D.C.	North America	Convention	Airport
Washington, D.C. City Trip 5	Washington D.C.	North America	Convention	City
30th Street Station (Philadelphia)	Philadelphia	North America	Station	Airport
Grand Central Station (NYC)	New York City	North America	Station	City
Jamaica Station (Queens)	New York City	North America	Station	Airport
Millenium Station (Chicago)	Chicago	North America	Station	Airport
Montreal Central Station	Montreal	North America	Station	Airport
Pennsylvania Station (Newark)	Newark	North America	Station	Airport
Pennsylvania Station (NYC)	New York City	North America	Station	City
Union Station (Chicago)	Chicago	North America	Station	Airport
Union Station (LA)	Los Angeles	North America	Station	Airport
Union Station (Toronto)	Toronto	North America	Station	City
Union Station (Washington, D.C.)	Washington D.C.	North America	Station	Airport
World Trade Center (NYC)	New York City	North America	Station	City

Acquisition of aircraft deplanement time series

Flightradar24 is an online platform that provides real-time flight information on commercial aircraft activity around the world. The service includes flight tracks, origins and destinations, aircraft call signs and types, positions, altitudes, headings and speeds. We use the platform's open API to compile landing time statistics about deplanements at the airports specified in Supplementary Tab. II.

Per deplanement at airport x, we create a landing event at time t provided as real landing time t_{real} and attach it with information on the aircraft's official call sign s and its current seat configuration c_s. We retrieve the information on current aircraft seat configuration via flightera.net, an online platform that provides detailed aircraft information per call sign. The list of tuples $(x, s, c_s)_{t_{\text{real}}}$ recorded over the time span T defines the aircraft deplanement time series that we use as a proxy to model the time-dependent demand for rides originating from the airports.

Acquisition of taxi trip records

The Department of For-Hire Vehicles and the D.C. Office of the Chief Technology Officer provide taxicab trip records for the Washington D.C. region. In our analysis, we focus on the data recorded between 17-01-02 and 17-08-27, including 34 full weeks of data. From this data set, we use a subset of approximately 370000 taxi trip records originating from Reagan National Airport (DCA) to compile spatio-temporarily resolved trip statistics for the average demand for taxi-like mobility services. We use taxi trips' origin-destination timestamps, their zip codes as well as longitudes and latitudes to determine a statistical demand model per day of week.

Acquisition of foreign currency exchange rates

Ratesapi.io is a publicly accessible API that provides current and historical foreign exchange rates for different currencies based on the data made available through the European Central Bank. We use the API to convert Uber price estimates in non-USD countries into USD with the help of the exchange rate for the respective day.

Acquisition of timezone information

We gathered the time zones for each airport using a JSON database from `https://github.com/mwgg`, (accessed on 2019-07-12). For city, convention and station trips we entered the time zone information manually. The conversion between the measured time in CEST and the desired timezones was done using python's pytz module, which takes care of daylight saving times automatically.

Data content and quality

In this subsection, we detail the data quality per source and the data cleansing methodology applied to obtain the data serving as inputs for our analyses.

Uber price estimate time series

Uber price estimates provide possible fare ranges for the requested ride, upper and lower bounded by the price vectors $\mathbf{p}_{\max}$ and $\mathbf{p}_{\min}$. $\mathbf{p}_{\max}$ and $\mathbf{p}_{\min}$ are provided as integer values in the local currency by the Uber API. Typically, the difference between $\mathbf{p}_{\max}$ and $\mathbf{p}_{\min}$ is fixed for most of the time (e.g. 2 USD). For our analyses, we consider the lower bound of the price estimate only.

Price estimates do not update continuously, but only approximately every two minutes (compare Supplementary Fig. 2). Hence, price estimate time series $\{((o,d), \mathbf{p}_{\min}, l, t_d)_t, t \in T\}$ contain identical elements if sampling at higher frequency than two minute intervals. We provide detailed information on the sampling frequency per recorded trip request in the spreadsheet DataRideHailingPrices.xlsx available in the online Supplementary Information.

We do not clean the price estimate time series and use the raw data as inputs for our analyses as described in more detail in the following subsection.

Aircraft deplanement time series

In rare occasions, flightradar24's API does not return a real landing time, or information on the airplane's call sign. In those cases, we assume the flight to be cancelled and exclude the event from the time series that we use for our analyses.

Furthermore, in few cases there is no information on the current seat configuration available on flightera.net under the provided aircraft call sign. In those cases, we use the aircraft model to estimate the number of seats from data containing call signs. First we match all aircraft arrivals with known call signs with the number of seats obtained from flightera.net. We then compute the average number of seats for every aircraft model. Arrivals without a call sign entry, or where flightera.net does not provide the seat configuration, are then assigned the average of the number of

seats according to the aircraft model. In cases where there is no number of seats information available for an aircraft model, we assume it to be the average over all aircraft models.

Comparing seat configurations across the aircraft in the deplanement time series reveals a maximum variation of 25 seats for aircraft of the same model (Boeing 757-232, equipped with 180-205 seats). This type of aircraft has landed seven times at DCA within the time frame considered in our time series. For smaller aircraft in the order of 50 seats, the maximum deviation in seat configuration was at most 20%.

Taxi trip records

Selected taxi trip records contain entries where geographical information are not properly decoded, longitude or latitude information indicate locations outside the US, or zip codes contain placeholders. Similarly, a small number of time stamps are not properly specified.

We filter these entries from the data recorded between 17-01-02-17-08-27, including 34 full weeks of records. Furthermore, our data cleansing procedure excludes data records less than 0.25 miles or more than 1000 miles.

Data processing

In this final subsection, we detail how we integrated and analyzed the cleaned data to obtain the results presented in the main manuscript and the first three sections of this Supplementary Information.

Isolation and characterization of Uber surge dynamics

For illustrations of the price time series we use the raw data recorded. To study the surge fee time series we subtract the trip fee and pickup fee of the respective product calculated from the recorded trip duration and distance estimates together with the trip fee parameters. This leaves only the surge fee and surcharges. Since data on some surcharges is not available from Uber's API, we assume that surcharges are constant in time.[3] We estimate these surcharges as the minimum value of the remaining surcharge plus surge fee. Subtracting this constant value results in an estimate of the absolute surge fee time series that attains a minimum value zero (no surge) at least once. Note that due to the rounding to integer values, the price estimate may not reflect all changes of the trip fare, leading to small fluctuations in the isolated surge fee that do not correspond to actual surge activity.

For better comparison between the different trips, we normalize the absolute surge fee time series by the base cost (sum of the pickup fee, trip fee and estimated surcharges) at that time, resulting in a relative surge factor time series for each trip and Uber product. In general, we observe that premium Uber services have less frequent surge fee contributions and we assume that these are typically caused by global changes such as strong demand-side price surges or network problems influencing the price estimates. The resulting data is, for example, shown in Supplementary Fig. 2 above.

To analyze the changes in the surge factor time series, we first create a uniform representation of the surge factor time series in one minute intervals. Each point in the new time series is calculated as the average in the five minute time window centered around t for the respective combination of trip origin o, destination d. Each component of the price vector corresponds to an Uber service. Data points include information about approximately three updates of the price estimate. Based on this data, we calculate the relative price changes as the difference between consecutive time steps and discard any changes smaller than $\Delta p^2 < 10^{-7}$. The remaining changes form the basis for the analysis and are illustrated in the histograms in Supplementary Fig. 12.

Computation of cross-correlations between aircraft deplanement and Uber price estimate time series

The aircraft arrival data has a maximum resolution of one minute, with some minutes without arrivals. Hence, we first aggregate the number of seats for each minute, and set minutes without arrivals to zero. Then we compute the

[3] This is not accurate in individual cases where airport pickup fees may change over time or tolls may be in effect during the day but not at night.

moving average with a window of five minutes, to reduce noise and to remain consistent over the treatment of our time series.

We compute the surge fee component from the Uber price estimate according to Eq. (S1). Because we have a much larger but irregular resolution for the Uber price estimates, we compute the moving average of the surge fee and compute the averages for every minute. This leaves us with the same granularity of the data as the deplanement data.

In the next step, we select a time window from the surge fee time series. We normalize this fragment of the series by subtracting its mean and dividing by the standard deviation.

We then iterate over various lags Δt. For each lag, we shift the deplanement data by the corresponding amount and select the overlap with the surge fee window. We compute the Pearson correlation coefficient ρ for these two time series according to Eq. (S2).

We repeat this procedure for different time windows (8:00 to 14:00, 14:00 to 20:00, 20:00 to 02:00, 08:00 to 20:00, 14:00 to 02:00, 08:00 to 02:00) and data from different days (19-06-04, 19-06-05 and 19-06-06), see Supplementary Fig. 5.

Computation of taxi trip statistics

We organize the taxi trip data from the period of 17-01-02-17-08-27 by weekdays (Monday, Tuesday, etc.). For each weekday we determine the average number of taxi trips starting at Reagan National Airport for every minute of the day.

Because of irregularities in the timestamps of the data we are not able to obtain data for some minutes. Therefore, we set missing values to zero, and correct them by computing a moving average with a window of five minutes.

Supplementary References

[1] Sweeney, S. Uber, Lyft drivers manipulate fares at Reagan National causing artificial price surges. https://wjla.com/news/local/uber-and-lyft-drivers-fares-at-reagan-national (2019).
[2] Mehta, N., Detroja, P. & Agashe, A. How and why Amazon changes its prices so often. https://www.businessinsider.de/amazon-price-changes-2018-8?r=US&IR=T (2018).
[3] Uber Technologies, Inc. How surge pricing works. https://www.uber.com/de/drive/partner-app/how-surge-works/ (2018).
[4] Lyft, Inc. How to estimate a Lyft ride's cost. https://help.lyft.com/hc/en-us/articles/115013080308-How-to-estimate-a-Lyft-ride-s-cost (2019).
[5] Uber Technologies, Inc. Uber cities across the globe. https://www.uber.com/en/cities/ (2019).
[6] Garg, N. & Nazerzadeh, H. Driver surge pricing. Preprint at http://arxiv.org/abs/1905.07544 (2019).
[7] Uber Technologies, Inc. Uber estimate - get a price estimate in your city. https://www.uber.com/de/en/price-estimate/ (2019).
[8] Uber Technologies, Inc. Your questions about the new surge, answered. https://www.uber.com/blog/your-questions-about-the-new-surge-answered/ (2018).
[9] Github repository 'verge-uber-launch-dates' maintained by Frank Bi. Contains a public dataset of launch dates for Uber ride-hailing services in U.S. cities and abroad, last updated 2016-01-27. https://github.com/voxmedia/data-projects/tree/master/verge-uber-launch-dates (2019).
[10] Department of For-Hire Vehicles. Surcharge generated by taxicabs, private sedans and black cars. https://dfhv.dc.gov/page/dfhv-dashboard-and-statistical-data-sets (2019).
[11] Skyrms, B. *The Stag Hunt And The Evolution of Social Structure* (Cambridge University Press, Cambridge, 2003).
[12] Kuhn, S. *Prisoner's Dilemma* (The Stanford Encyclopedia of Philosophy. Metaphysics Research Lab, Stanford University, 2019).
[13] Uber Technologies, Inc. Facts & figures as of December 2018. https://www.uber.com/en-PK/newsroom/company-info/ (2019).
